# A Recyclable Inorganic Lanthanide Cluster Catalyst for Chemoselective Aerobic Oxidation of Thiols

**DOI:** 10.3390/molecules29143361

**Published:** 2024-07-17

**Authors:** Lijun Wang, Zixuan Qin, Lingxia Chen, Xinshu Qin, Jiaman Hou, Chao Wang, Xuan Li, Hongxia Duan, Bing Fang, Minlong Wang, Jie An

**Affiliations:** 1 Department of Nutrition and Health, China Agricultural University, Beijing 100193, China; lijunwang@cau.edu.cn (L.W.); shxzq1@nottingham.edu.cn (Z.Q.); lxchen915@163.com (L.C.); qinxs98@163.com (X.Q.); y20223313563@cau.edu.cn (J.H.); b20233311213@cau.edu.cn (C.W.); sy20233313716@cau.edu.cn (X.L.); jie_an@cau.edu.cn (J.A.); 2 Department of Chemistry, College of Science, China Agricultural University, Beijing 100193, China; hxduan@cau.edu.cn

**Keywords:** lanthanide cluster, aerobic oxidation, disulfides

## Abstract

Optimizing lanthanide catalyst performance with organic ligands often encounters significant challenges, including susceptibility to water or oxygen and complex synthesis pathways. To address these issues, our research focuses on developing inorganic lanthanide clusters with enhanced stability and functionality. In this study, we introduce the [Sm_6_O(OH)_8_(H_2_O)_24_]I_8_(H_2_O)_8_ cluster (Sm-OC) as a sustainable and efficient catalyst for the aerobic oxidation of thiols under heating conditions. The Sm-OC catalyst demonstrated remarkable stability, outstanding recyclability, and excellent chemoselectivity across a diverse range of functional groups in 38 different tests. Notably, it enables efficient unsymmetrical disulfide synthesis and prevents the formation of over-oxidized by-products, highlighting its superior performance. This Sm-OC catalyst provides a practical and robust tool for the precise construction of versatile disulfides, thus establishing a template for the broader use of lanthanide clusters in organic synthesis.

## 1. Introduction

In the field of organic synthesis, lanthanide coordination compounds have found widespread use as mild, stable, and selective homogeneous catalysts, sparking extensive interest among researchers [1,2,3,4,5]. Organic ligands are always required to achieve the best performance of lanthanide catalysts, such as the chiral-cyclopentadienyl ligand, which was used for the asymmetric hydroamination of cyclopropenes and the enantioselective C–H bond addition of pyridines to alkenes [6,7,8,9,10]. However, the use of organic ligands often introduces challenges. Lanthanide organic complexes are susceptible to environmental influences, like water and oxygen, leading to a decrease in reactivity [9,10,11,12,13]. In addition, the synthesis route using organic ligands becomes more intricate and not environmentally friendly [12,13,14,15,16]. This has limited their practical application in certain contexts. To address these limitations, our work focuses on the development of inorganic lanthanide clusters as a catalyst with excellent stability for organic synthesis [17,18,19].

**Figure 1:**
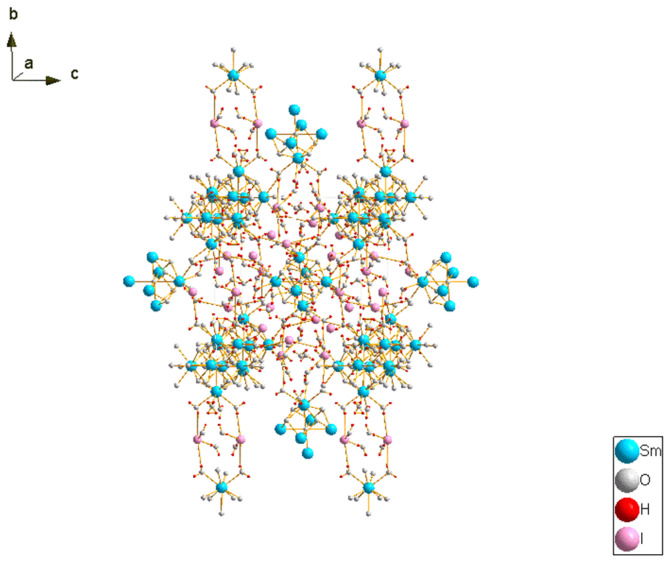
The structure of Sm-OC.

In our previous study, we developed a unique class of a well-defined polyhedral lanthanide-oxo/hydroxo cluster [Sm_6_O(OH)_8_(H_2_O)_24_]I_8_(H_2_O)_8_ (Sm-OC) with the cationic moiety [Sm_6_O(OH)_8_(H_2_O)_24_]^8+^ and the anionic moiety I^−^ shown in Figure 1 and applied Sm-OC as a photocatalyst in a catalytic aerobic oxidation reaction. By using a distinctive auxiliary ligand-free oxidative hydrolysis method, Sm-OC can be easily prepared in the multigram scale. And that study presented the first application of the lanthanide-oxo/hydroxo cluster as a photocatalyst [20]. To further explore the protentional application of Sm-OC in the field of catalysis, this study investigated the application of Sm-OC as a catalyst under heating conditions in the synthesis of disulfides via the aerobic oxidation of thiols, accompanied by a cycling experiment. Notably, Sm-OC as a catalyst was first employed for the synthesis of disulfides under heating conditions. Organic disulfides constitute a common class of compounds in organic chemistry and biology, holding significant importance. Their applications range from antioxidants, pharmaceuticals, and pesticides to rubber vulcanizing agents [21,22,23,24,25,26,27,28,29]. The easy interconversion between thiols and disulfides, coupled with the latter’s higher stability, often leads to disulfides being used as a source for thiols [28,30]. Most methods for synthesizing disulfides involve the oxidation of thiols. However, traditional methods for thiol oxidation often lead to the formation of over-oxidized by-products and pose challenges in oxidizing tertiary thiols [31]. The current strategies for disulfide bond synthesis face certain challenges, necessitating the development of more efficient and sustainable methods.

This study exhibits good tolerance and excellent chemselectivity, showcasing the capability to oxidize primary, secondary, and tertiary thiols without over-oxidized by-products, particularly in cyclization reactions. The use of Sm-OC as a catalyst represents the first application for synthesizing disulfides under heating conditions, marking the pioneering utilization of lanthanide-oxo/hydroxy clusters under heating condition.

## 2. Results and Discussion

Our investigation commenced with reaction condition optimization of the Sm-OC catalyzed aerobic oxidation of thiols, selecting dodecane-1-thiol **1a** as the model substrate (Table 1). Employing ethyl acetate (EtOAc) as the solvent under aerobic conditions with a catalyst loading of 1 mol%, we achieved excellent yields without the formation of over-oxidized by-products (entry 4, Table 1). Subsequently, a significant improvement from **1a** to **2a** in yield was observed as the catalyst loading decreased from 10.0% to 1 mol%, while reducing the catalyst loading to 0.2 mol% resulted in incomplete oxidation of **1a** (entries 1–5, Table 1). The influence of various solvents on the reaction was further explored, revealing high sensitivity to solvent selection (entries 6–10, Table 1). Despite the solubility of Sm-OC in polar solvents like EtOAc, the yield of **2a** in EtOAc and tetrahydrofuran (THF) surpassed that in methanol (MeOH), ethanol (EtOH), and acetonitrile (CH_3_CN). This observation underscores the significant impact of solvent selection on catalytic reactions. Considering the solvent toxicity factor, EtOAc was chosen as the reaction solvent. After determining the optimal catalyst load and reaction time, we assessed the reaction temperature. The results demonstrated that the reaction at room temperature resulted in a lower yield of 42% (entry 10, Table 1), whereas the reaction at 70 °C exhibited a notably higher yield, exceeding >98%. Therefore, the optimal temperature for this reaction was established as 70 °C. Finally, the influence of reaction time was also investigated. The findings revealed that decreasing the reaction time from 16 h to 1 h led to a decrease in yield from 98% to 13%, indicating that prolonging the reaction time to 16 h can ensure the complete conversion of **1a** to **2a** (entries 11–12, Table 1). After determining the optimal conditions, we conducted catalyst recycling experiments. For homogeneous catalysts, easily recoverable and reusable catalysts are crucial as this directly impacts their catalytic efficiency [32,33,34]. Through two cycles, Sm-OC demonstrated high catalytic activity, yielding a 94% conversion rate for **2o** in the oxidation process. 

**Figure 2:**
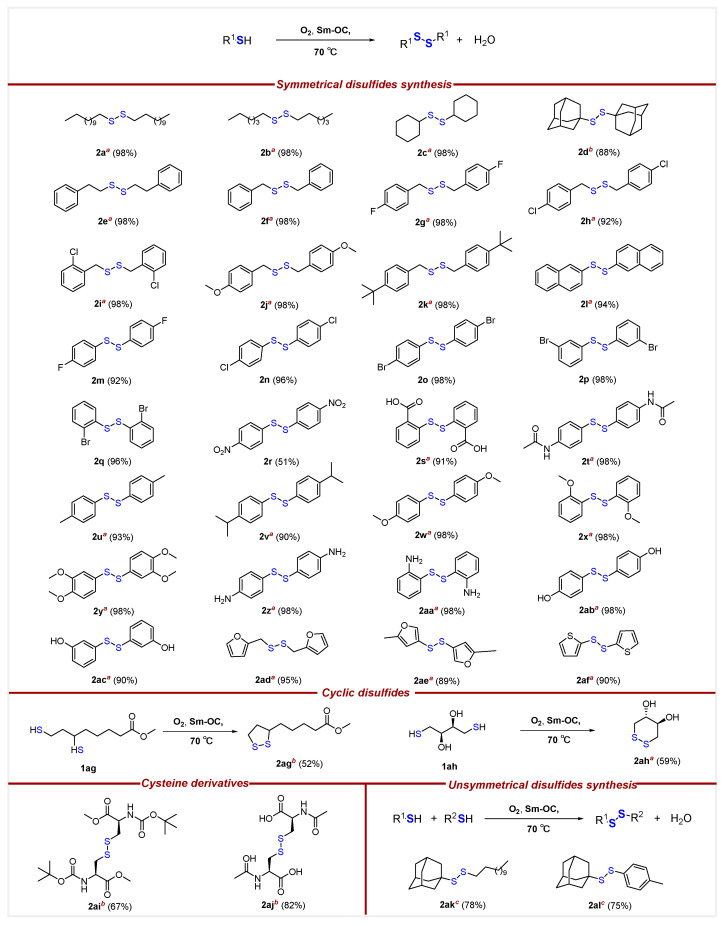
Substrate scope for the aerobic oxidation of thiols. *^a^* Conditions: Sm-OC (1.15 mol%) were added to a solution of thiols (0.300 mmol, 1.00 equiv.) in AcOEt (8.00 mL) under O_2_ balloon at 70 °C and stirred for a duration of 16 h. *^b^* Sm-OC (2.30 mol%) were added to a solution of thiols (0.300 mmol, 1.00 equiv.) in AcOEt (8.00 mL) under O_2_ balloon at 70 °C and stirred for a duration of 16 h. *^c^* Sm-OC (2.30 mol%) were added to a solution of thiols (0.300 mmol, 1.00 equiv.) and (3*s*,5*s*,7*s*)-adamantane-1-thiol (0.900 mmol, 3.00 equiv.) in AcOEt (8.00 mL) under O_2_ balloon at 70 °C and stirred for a duration of 16 h.

Based on a comprehensive examination of reaction conditions, we concluded that 1 mol% Sm-OC in EtOAc at 70 °C for 16 h is suitable for investigating the substrate scope (Figure 2). The substrate scope studies indicated that the aerobic oxidation catalyzed by Sm-OC can transform all 38 tested thiols into disulfides with high yields, and there is no evidence for the formation of sulfonic acid. First, a series of thiols were tested for the synthesis of symmetrical disulfides, showing that aromatic, aliphatic, and poly-aromatic thiols transformed into disulfides in good to excellent yields. Specifically, primary, secondary, and tertiary thiols exhibited high reactivity. Notably, the aerobic oxidation process displayed a significant ability to overcome the inherent challenges commonly associated with tertiary thiol, exemplified by the observed high efficacy in the conversion of **1x** to **2x**. The reaction conditions show good tolerance towards various functional groups. Specifically, aryl thiols bearing both electron-donating functional groups, like methoxyl (**2j**, **2w**, **2x**, **2y**), tert-butyl (**2k**), methyl (**2u**), isopropyl (**2v**), amino (**2z**, **2aa**), hydroxyl (**2ab**, **2ac**), as well as electron-with drawing groups, including fluoro (**2g**, **2m**), chloro (**2h**, **2n**), bromo (**2o**, **2p**, **2q**), nitro (**2r**), carboxyl (**2s**), and acetylamino (**2t**), afforded the corresponding disulfides in good to excellent yields. Additionally, excellent yields were achieved for aryl thiols with substitutions at the para-, meta-, or ortho-positions, as well as those with multiple substituents on the benzene ring. Expanding beyond the mentioned substrates, this method effectively promotes the conversion of polyaromatic thiol (**2l**) and heterocyclic thiol (**2ad**, **2ae**, **2af**) into their respective disulfides. Among the tested substrates, there is no sign of over-oxidation of the disulfides or adverse oxidation of non-target functional groups, which highlighted the exceptional chemoselectivity of this oxidation reaction. 

To assess the practicality of the reaction conditions, intramolecular disulfide bond forming reactions were conducted. Cyclic disulfides are known for their complexity and difficulty to synthesize using conventional methods. Our protocol can transform the reduced forms into the derivative of α-lipoic acid known as α-lipoic acid methyl ester **2ag**, with a yield of 52%. This substance exhibits certain biological activities [35]. Our protocol was also applied to the oxidation of dithiothreitol, and the conversion of **1ah** to **2ah** was achieved with a yield of 59%. It is noteworthy that trans-4,5-dihydroxy-1,2-dithiane formed in the process acts as an inducer of endoplasmic reticulum stress proteins, offering protection to the kidneys from chemical stress in vivo [36]. Additionally, the versatility of the reaction was demonstrated in the oxidative construction of disulfide bonds between cysteine derivatives used for acute paracetamol toxicity and peptide synthesis [37,38], namely *N*-(tert-butoxycarbonyl)-*L*-cysteine methyl ester **1ai** and *N*-acetyl-*L*-cystine **1aj**, yielding 67% and 82% in the formation of the corresponding disulfides **2ai** and **2aj**, respectively. These results not only underscore the practicality and effectiveness of this method but also highlight its potential in synthesizing new materials and biologically active disulfides.

Given the importance of unsymmetrical disulfides in many fields, our method was successfully employed for the synthesis of asymmetric disulfides. Different from the symmetrical disulfides, the unsymmetrical disulfides present an inherent challenge in synthesis with high chemoselectivity [31,39]. Nevertheless, the present protocol could overcome this limitation, enabling the synthesis of unsymmetrical disulfides with a good isolated yield using three equivalents of tertiary thiol (**2ak**, **2al**). 

Following the establishment of optimized conditions and confirmation of the applicability of this protocol, a series of controlled reactions were conducted to understand this oxidation process. Initially, it was established that the reaction could not proceed without Sm-OC (entry 1, Table 2), emphasizing the indispensability of the catalyst. This prompts us to carry out cycle tests to access the regeneration capability of Sm-OC. After two consecutive cycles, we observed a remarkable 94% yield in the conversion to the corresponding disulfide, underscoring its high catalytic activity and renewable capacity of Sm-OC. Considering that [Sm_6_O(OH)_8_(H_2_O)_24_]^8+^ carries a positive charge and coexists with iodide ions, control experiments of iodide ions dissolved in EtOAc were performed. The result showed that no disulfides appeared under this condition (entries 2–3, Table 2). This effectively excluded the possibility of iodide ions serving as catalysts, emphasizing the crucial role of [Sm_6_O(OH)_8_(H_2_O)_24_]^8+^. Although Sm(III) is involved in the formation of [Sm_6_O(OH)_8_(H_2_O)_24_]^8+^, generating disulfides in control reactions using Sm_2_O_3_ or SmCl_3_ was unsuccessful. This confirmed that the unique structure of [Sm_6_O(OH)_8_(H_2_O)_24_]^8+^ plays a crucial role in its catalytic activity under heating and aerobic conditions (entries 4–5 Table 2). 

## 3. Materials and Methods

### 3.1. General Information

Glassware was dried in an oven overnight before use. Thin-layer chromatography was carried out on SIL G/UV254 silica-glass plates provided by Tansoole (Shanghai, Bejing, China), and plates were visualized using ultra-violet light (254 nm) and KMnO_4_ solution. For flash column chromatography, silica gel 60 35–70 μm was used. NMR data were collected at 500 MHz. Data were manipulated directly from the spectrometer or via a networked PC with appropriate software. All samples were analyzed in CDCl_3_ unless otherwise stated. Reference values for residual solvent were taken as δ = 7.27 (CDCl_3_), δ = 2.50 (DMSO-*d*_6_), δ = 4.79 (D_2_O) and δ = 3.31 (CD_3_OD) for ^1^H NMR; δ = 77.1 (CDCl_3_), δ = 39.5 (DMSO-*d*_6_), δ = 49.0 (CD_3_OD) for ^13^C{^1^H} NMR. Multiplicities for coupled signals were designated using the following abbreviations (given in Hz): s = singlet, d = doublet, t = triplet, q = quartet, quin = quintet, br = broad signal. ^1^H NMR and ^13^C{^1^H} NMR data were showed in Supporting Information.

Reagents and solvents were purchased from commercial suppliers and used directly without further purification, unless otherwise noted. Sm metal (25 mesh) was purchased from Hebei Zhongyue Metal Materials Technology Co., Ltd. (Shijiazhuang, China). All other reagents were purchased from Energy Chemical (Huangshan, China) or InnoChem (Beijing, China). All water was deionized before use. Unless otherwise noted, all reactions were carried out in glassware, which was dried in an oven overnight before use. ‘Room temperature’ ranged from 20 to 25 °C. The oxygen purity used in the experiment was 99.999%.

The synthesis method of catalyst Sm-OC is reported in the literature [20]. The specific procedural steps are as follows: The reactions of excessive Sm metal (1.80 g, 12.0 mmol) and the purified 1,2-diiodoethane (1.69 g, 6.00 mmol) were weighed to a 250 mL round-bottomed flask with septum. Carefully, argon was introduced to the 250 mL round-bottomed flask for about 20 min to ensure an air-free atmosphere. Then, 60.0 mL extra-dry THF was transferred to the 250 mL round-bottomed flask containing samarium metal and 1,2-diiodoethane using multiple 50 mL syringes. And the reaction mixture was stirred under Ar atmosphere at room temperature. After 18 h, a deep-blue solution of SmI_2_ (0.100 M) was formed. The reaction mixture was allowed to settle for 30 min. Then, 24.0 mL of the SmI_2_ solution was transferred to a flask sealed by a rubber plug and filled with Ar. The solution of SmI_2_ was oxidized using a balloon of O_2_. After the color of the solution turned yellow, deionized water (0.274 g, 15.2 mmol) was added under Ar and the reaction mixture was stirred at room temperature for 5 h. The orange-red solution was concentrated using a rotary evaporator. Then, the residual trace solvent was removed by overnight exposure on the vacuum line to give yellow solid Sm-OC.

### 3.2. Calculation of the Yield by Internal Standard Using ^1^H NMR

Determination of yields by ^1^H NMR was according to the equation below:

(1) $$\mathrm{Yield}=\left(\frac{{\mathrm{Area}}_{\mathrm{product}}}{{\mathrm{Area}}_{\mathrm{internal}\;\mathrm{standard}}}\right)\left(\frac{n_{\mathrm{internal}\;\mathrm{standard}}}{n_{\mathrm{theoretical}\;\mathrm{product}}}\right)\times 100\%$$

Area_product_ means the integration of the product peak, Area_internal standard_ means the integration of the internal standard peak, n_internal standard_ means the number of moles of the internal standard, n_theoretical product_ means the theoretical number of moles of the product.

### 3.3. Optimization Studies for the Oxidative Coupling of Thiols by Sm-OC (Table 1)

To a round-bottom flask, Sm-OC (2.00–90.0 mg, 0.200–11.5 mol%), dodecane-1-thiol (60.7 mg, 0.300 mmol, 1.00 equiv.), and different extra-dry solvents (8.00 mL) were added under different atmospheres at different temperatures and stirred vigorously for a period of 1–16 h. Subsequently, the reaction mixture was diluted with EtOAc (10.0 mL) and washed with HCl solution (15.0 mL, 0.100 M, aq). The aqueous layer was extracted with EtOAc (3 × 15.0 mL). Organic layers were combined, dried over MgSO_4_, filtered, and concentrated under vacuum to give the product. The sample was then analyzed by ^1^H NMR (CDCl_3_, 500 MHz) to obtain the yield using internal standard (1,1,2,2-tetrachloroethane) and comparison with corresponding samples. 

### 3.4. General Procedure for the Oxidation of Thiols

To a round-bottom flask in oil-bath, Sm-OC (9.10 mg, 1.15 mol%), thiol (0.300 mmol, 1.00 equiv.), and extra-dry EtOAc (8.00 mL) were added. The flask was filled with oxygen balloon (0.3 MPa) and the reaction mixture was stirred at 70 °C for a duration of 16 h. Subsequently, the reaction mixture was cooled by removing from the oil-bath. The reaction mixture was diluted with EtOAc (10.0 mL) and HCl (15.0 mL, 0.100 M, aq). The aqueous layer was extracted with EtOAc (3 × 15.0 mL). Organic layers were combined, dried over MgSO_4_, filtered, and concentrated to yield the product. Then, the sample was analyzed by ^1^H NMR (CDCl_3_, 300 MHz) to obtain the yield using internal standard (1,1,2,2-tetrachlorethan) and comparison with corresponding samples.

*1,2-Didodecyldisulfane* (**2a**) [37]. According to the general procedure, the reaction of Sm-OC (9.10 mg, 1.15 mol%) and dodecane-1-thiol (60.7 mg, 0.300 mmol) afforded 59.2 mg of **2a** in 98% yield as a white solid. ^1^H NMR (500 MHz, CDCl_3_) δ 2.69 (t, *J* = 7.4 Hz, 4H), 1.68 (m, 4H), 1.39 (m, 4H), 1.35–1.21 (m, 32H), 0.89 (t, *J* = 6.9 Hz, 6H); ^13^C{^1^H} NMR (126 MHz, CDCl_3_) δ 39.3, 32.0, 29.8, 29.7 (×2), 29.6, 29.5, 29.4, 29.3, 28.6, 22.8, 14.2.

*1,2-Dihexyldisulfane* (**2b**) [40]. According to the general procedure, the reaction of Sm-OC (9.10 mg, 1.15 mol%) and hexane-1-thiol (35.5 mg, 0.300 mmol) afforded 34.5 mg of **2b** in 98% yield as a colorless oil. ^1^H NMR (500 MHz, CDCl_3_) δ 2.69 (t, *J* = 7.4 Hz, 4H), 1.68 (m, 4H), 1.39 (m, 4H), 1.36–1.24 (m, 8H), 0.90 (t, *J* = 6.9 Hz, 6H); ^13^C{^1^H} NMR (126 MHz, CDCl_3_) δ 39.3, 31.5, 29.3, 28.3, 22.6, 14.1.

*1,2-Dicyclohexyldisulfane* (**2c**) [40]. To a round-bottom flask in oil-bath, Sm-OC (9.10 mg, 1.15 mol%), cyclohexanethiol (58.1 mg, 0.500 mmol), and extra-dry EtOAc (8.00 mL) were added. The flask was filled with oxygen balloon (0.3 Mpa) and the reaction mixture was stirred at 70 °C for a duration of 16 h. Subsequently, the reaction mixture was cooled by removing from the oil-bath. The reaction mixture was diluted with EtOAc (10 mL) and HCl (15.0 mL, 0.100 M, aq). The aqueous layer was extracted with EtOAc (3 × 15.0 mL). Organic layers were combined, dried over MgSO_4_, filtered, and concentrated, affording 56.5 mg of **2c** in 98% yield as a colorless oil. ^1^H NMR (500 MHz, CDCl_3_) δ 2.69 (m, 2H), 2.05 (m, 4H), 1.79 (m, 4H), 1.61 (m, 2H), 1.39–1.17 (m, 10H); ^13^C{^1^H} NMR (126 MHz, CDCl_3_) δ 50.1, 33.0, 26.2, 25.8.

*1,2-Di((3S,5S,7S)-adamantan-1-yl)disulfane* (**2d**) [41]. To a round-bottom flask in oil-bath, Sm-OC (18.2 mg, 2.30 mol%), (3*s*,5*s*,7*s*)-adamantane-1-thiol (50.5 mg, 0.300 mmol), and extra-dry EtOAc (16.0 mL) were added. The flask was filled with oxygen balloon (0.3 MPa) and the reaction mixture was stirred at 70 °C for a duration of 16 h. Subsequently, the reaction mixture was cooled by removing from the oil-bath. The reaction mixture was diluted with EtOAc (10.0 mL) and NaOH solution (15.0 mL, 1.00 M, aq). The aqueous layer was extracted with EtOAc (3 × 15.0 mL). Organic layers were combined, dried over MgSO_4_, filtered, and concentrated, affording 44.2 mg of **2d** in 88% yield as a white solid. ^1^H NMR (500 MHz, CDCl_3_) δ 2.07 (m, 6H), 1.82 (m, 12H), 1.67 (m, 12H); ^13^C{^1^H} NMR (126 MHz, CDCl_3_) δ 47.4, 43.2, 36.2, 30.1.

*1,2-Diphenethyldisulfane* (**2e**) [42]. To a round-bottom flask in oil-bath, Sm-OC (9.10 mg, 1.15 mol%), 2-phenylethane-1-thiol (69.1 mg, 0.500 mmol), and extra-dry EtOAc (8.00 mL) were added. The flask was filled with oxygen balloon (0.3 MPa) and the reaction mixture was stirred at 70 °C for a duration of 16 h. Subsequently, the reaction mixture was cooled by removing from the oil-bath. The reaction mixture was diluted with EtOAc (10.0 mL) and HCl (15.0 mL, 0.100 M, aq). The aqueous layer was extracted with EtOAc (3 × 15.0 mL). Organic layers were combined, dried over MgSO_4_, filtered, and concentrated, affording 67.2 mg of **2e** in 98% yield as a colorless oil. ^1^H NMR (500 MHz, CDCl_3_) δ 7.30–7.27 (m, 4H), 7.23–7.16 (m, 6H), 2.98 (m, 4H), 2.92 (m, 4H); ^13^C{^1^H} NMR (126 MHz, CDCl_3_) δ 140.1, 128.7, 128.6, 126.5, 40.3, 35.8.

*1,2-Dibenzyldisulfane* (**2f**) [43]. According to the general procedure, the reaction of Sm-OC (9.10 mg, 1.15 mol%) and phenylmethanethiol (37.3 mg, 0.300 mmol) afforded 36.2 mg of **2f** in 98% yield as a white solid. ^1^H NMR (500 MHz, CDCl_3_) δ 7.25–7.11 (m, 10H), 3.49 (s, 4H); ^13^C{^1^H} NMR (126 MHz, CDCl_3_) δ 137.4, 129.4, 128.5, 127.4, 43.3.

*1,2-Bis(4-fluorobenzyl)disulfane* (**2g**) [44]. According to the general procedure, the reaction of Sm-OC (9.10 mg, 1.15 mol%) and (4-fluorophenyl)methanethiol (42.7 mg, 0.300 mmol) afforded 41.5 mg of **2g** in 98% yield as a yellow solid. ^1^H NMR (500 MHz, CDCl_3_) δ 7.21 (m, 4H), 7.02 (m, 4H), 3.60 (s, 4H); ^13^C{^1^H} NMR (126 MHz, CDCl_3_) δ 162.3 (d, *J*_C-F_ = 246.4 Hz), 133.2 (d, *J*_C-F_ = 2.8 Hz), 131.0 (d, *J*_C-F_ = 8.5 Hz), 115.5 (d, *J*_C-F_ = 21.5 Hz), 42.5.

*1,2-Bis(4-chlorobenzyl)disulfane* (**2h**) [45]. According to the general procedure, the reaction of Sm-OC (9.10 mg, 1.15 mol%) and (4-chlorophenyl)methanethiol (47.6 mg, 0.300 mmol) afforded 43.5 mg of **2h** in 92% yield as a white solid. ^1^H NMR (500 MHz, CDCl_3_) δ 7.31 (m, 4H), 7.17 (m, 4H), 3.59 (s, 4H); ^13^C{^1^H} NMR (126 MHz, CDCl_3_) δ 135.9, 133.5, 130.7, 128.8, 42.6.

*1,2-Bis(2-chlorobenzyl)disulfane* (**2i**) [46]. According to the general procedure, the reaction of Sm-OC (9.10 mg, 1.15 mol%) and (2-chlorophenyl)methanethiol (47.6 mg, 0.300 mmol) afforded 46.3 mg of **2i** in 98% yield as a white solid. ^1^H NMR (500 MHz, CDCl_3_) δ 7.29 (m, 2H), 7.18 (m, 2H), 7.16–7.11 (m, 4H), 3.70 (s, 4H); ^13^C{^1^H} NMR (126 MHz, CDCl_3_) δ 135.1, 134.2, 131.6, 129.8, 129.0, 126.8, 41.2.

*1,2-Bis(4-methoxybenzyl)disulfane* (**2j**) [36]. According to the general procedure, the reaction of Sm-OC (9.10 mg, 1.15 mol%) and (4-methoxyphenyl)methanethiol (46.3 mg, 0.300 mmol) afforded 45.0 mg of **2j** in 98% yield as a white solid. ^1^H NMR (500 MHz, CDCl_3_) δ 7.19 (m, 4H), 6.87 (m, 4H), 3.81 (s, 6H), 3.61 (s, 4H); ^13^C{^1^H} NMR (126 MHz, CDCl_3_) δ 159.1, 130.6, 129.5, 114.0, 55.4, 42.8.

*1,2-Bis(4-(tert-butyl)benzyl)disulfane* (**2k**) [44]. According to the general procedure, the reaction of Sm-OC (9.10 mg, 1.15 mol%) and (4-(*tert*-butyl)phenyl)methanethiol (54.1 mg, 0.300 mmol) afforded 52.7 mg of **2k** in 98% yield as a colorless oil. ^1^H NMR (500 MHz, CDCl_3_) δ 7.37 (m, 4H), 7.21 (m, 4H), 3.63 (s, 4H), 1.34 (s, 18H); ^13^C{^1^H} NMR (126 MHz, CDCl3) δ 150.5, 134.3, 129.2, 125.5, 43.1, 34.6, 31.4.

*1,2-Di(naphthalen-2-yl)disulfane* (**2l**) [43]. According to the general procedure, the reaction of Sm-OC (9.10 mg, 1.15 mol%) and naphthalene-2-thiol (48.1 mg, 0.300 mmol) afforded 44.9 mg of **2l** in 94% yield as a white solid. ^1^H NMR (500 MHz, CDCl_3_) δ 8.01 (m, 2H), 7.85–7.78 (m, 4H), 7.75 (m, 2H), 7.65 (m, 2H), 7.52–7.43 (m, 4H); ^13^C{^1^H} NMR (126 MHz, CDCl_3_) δ 134.3, 133.5, 132.6, 129.1, 127.8, 127.5, 126.8, 126.6, 126.3, 125.7.

*1,2-Bis(4-fluorophenyl)disulfane* (**2m**) [43]. To a round-bottom flask in oil-bath, Sm-OC (9.10 mg, 1.15 mol%), 4-fluorobenzenethiol (64.1 mg, 0.500 mmol), and extra-dry EtOAc (8.00 mL) were added. The flask was filled with oxygen balloon (0.3 MPa) and the reaction mixture was stirred at 70 °C for a duration of 16 h. Subsequently, the reaction mixture was cooled by removing from the oil-bath. The reaction mixture was diluted with EtOAc (10.0 mL) and HCl (15.0 mL, 0.100 M, aq). The aqueous layer was extracted with EtOAc (3 × 15.0 mL). Organic layers were combined, dried over MgSO_4_, filtered, and concentrated, affording 58.5 mg of **2m** in 92% yield as a white solid. ^1^H NMR (500 MHz, CDCl_3_) δ 7.46 (m, 4H), 7.02 (m, 4H); ^13^C{^1^H} NMR (126 MHz, CDCl_3_) δ 162.7 (d, *J*_C-F_ = 248.1 Hz), 132.3 (d, *J*_C-F_ = 2.8 Hz), 131.4 (d, *J*_C-F_ = 8.5 Hz), 116.4 (d, *J*_C-F_ = 22.0 Hz).

*1,2-Bis(4-chlorophenyl)disulfane* (**2n**) [43]. According to the general procedure, the reaction of Sm-OC (9.10 mg, 1.15 mol%) and 4-chlorobenzenethiol (72.3 mg, 0.500 mmol) afforded 68.9 mg of **2n** in 96% yield as a white solid: ^1^H NMR (500 MHz, CDCl_3_) δ 7.39 (m, 4H), 7.26 (m, 4H); ^13^C{^1^H} NMR (126 MHz, CDCl_3_) δ 135.2, 133.7, 129.4 (×2).

*1,2-Bis(4-bromophenyl)disulfane* (**2o**) [43]. According to the general procedure, the reaction of Sm-OC (9.10 mg, 1.15 mol%) and 4-bromobenzenethiol (56.7 mg, 0.300 mmol), after chromatography (silica, 100% Hexane) afforded 55.3 mg of **2o** in 98% yield as a white solid: ^1^H NMR (500 MHz, CDCl_3_) δ 7.44 (m, 4H), 7.35 (m, 4H); ^13^C{^1^H} NMR (126 MHz, CDCl_3_) δ 135.8, 132.3, 129.5, 121.6.

*1,2-Bis(3-bromophenyl)disulfane* (**2p**) [42]. According to the general procedure, the reaction of Sm-OC (9.10 mg, 1.15 mol%) and 3-bromobenzenethiol (56.7 mg, 0.300 mmol) afforded 55.3 mg of **2p** in 98% yield as a colorless oil. ^1^H NMR (500 MHz, CDCl_3_) δ 7.64 (m, 2H), 7.44–7.35 (m, 4H), 7.19 (m, 2H); ^13^C{^1^H} NMR (126 MHz, CDCl_3_) δ 138.7, 130.6 (×2), 130.0, 126.0, 123.2.

*1,2-Bis(2-bromophenyl)disulfane* (**2q**) [40]. According to the general procedure, the reaction of Sm-OC (9.10 mg, 1.15 mol%) and 2-bromobenzenethiol (56.7 mg, 0.300 mmol) afforded 54.2 mg of **2q** in 96% yield as a white solid. ^1^H NMR (500 MHz, CDCl_3_) δ 7.49–7.41 (m, 4H), 7.18 (m, 2H), 6.99 (m, 2H); ^13^C{^1^H} NMR (126 MHz, CDCl_3_) δ 136.2, 133.0, 128.3, 128.0, 127.0, 121.1.

*1,2-Bis(4-nitrophenyl)disulfane* (**2r**) [40]. According to the general procedure, the reaction of Sm-OC (9.10 mg, 1.15 mol%) and 4-nitrobenzenethiol (46.6 mg, 0.300 mmol) afforded 23.6mg of **2r** in 51% yield as a yellow solid. ^1^H NMR (500 MHz, DMSO-*d*_6_) δ 8.25 (m, 4H), 7.81 (m, 4H); ^13^C{^1^H} NMR (126 MHz, DMSO-*d*_6_) δ 146.6, 143.6, 126.7, 124.6.

*2,2′-Disulfanediyldibenzoic acid* (**2s**) [40]. To a round-bottom flask in oil-bath, Sm-OC (9.10 mg, 1.15 mol%), 2-mercaptobenzoic acid (46.3 mg, 0.300 mmol), and extra-dry EtOAc (8.0 mL) were added. The flask was filled with oxygen balloon (0.3 MPa) and the reaction mixture was stirred at 70 °C for a duration of 16 h. Subsequently, the reaction mixture was cooled by removing from the oil-bath. The reaction mixture was diluted with EtOAc (10.0 mL) and HCl (15.0 mL, 0.100 M, aq). The aqueous layer was extracted with EtOAc (3 × 15.0 mL), organic layers were combined, dried over MgSO_4_, filtered, and concentrated, after chromatography (0–10% MeOH/EtOAc), affording 41.8 mg of **2s** in 91% yield a white solid. ^1^H NMR (500 MHz, DMSO-*d*_6_) δ 8.02 (m, 2H), 7.61 (m, 2H), 7.54 (m, 2H), 7.32 (m, 2H); ^13^C{^1^H} NMR (126 MHz, DMSO-*d*_6_) δ 167.8, 138.9, 133.0, 131.5, 128.7, 125.9, 124.9.

*N,N’-(disulfanediylbis(4,1-phenylene))diacetamide* (**2t**) [40]. To a round-bottom flask in oil-bath, Sm-OC (9.10 mg, 1.15 mol%), *N*-(4-mercaptophenyl)acetamide (83.6 mg, 0.500 mmol), and extra-dry EtOAc (8.0 mL) were added. The flask was filled with oxygen balloon (0.3 MPa) and the reaction mixture was stirred at 70 °C for a duration of 16 h. Subsequently, the reaction mixture was cooled by removing from the oil-bath. The reaction mixture was diluted with EtOAc (10.0 mL) and HCl (15.0 mL, 0.100 M, aq). The aqueous layer was extracted with EtOAc (3 × 15.0 mL). Organic layers were combined, dried over MgSO_4_, filtered, and concentrated, affording 81.4 mg of **2t** in 98% yield as a white solid. ^1^H NMR (500 MHz, DMSO-*d*_6_) δ 10.07 (s, 2H), 7.59 (m, 4H), 7.42 (m, 4H), 2.04 (s, 6H); ^13^C{^1^H} NMR (126 MHz, DMSO-*d*_6_) δ 168.5, 139.5, 130.1, 129.4, 119.7, 24.0.

*1,2-Di-p-tolyldisulfane* (**2u**) [43]. According to the general procedure, the reaction of Sm-OC (9.10 mg, 1.15 mol%) and 4-methylbenzenethiol (37.3 mg, 0.300 mmol) afforded 34.4 mg of **2u** in 93% yield as a white solid. ^1^H NMR (500 MHz, CDCl_3_) δ 7.54 (m, 4H), 7.26 (m, 4H), 2.48 (s, 6H); ^13^C{^1^H} NMR (126 MHz, CDCl_3_) δ 137.5, 134.0, 129.9, 128.6, 21.1.

*1,2-Bis(4-isopropylphenyl)disulfane* (**2v**) [46]. To a round bottom flask in oil-bath, Sm-OC (9.10 mg, 1.15 mol%), 4-isopropylbenzenethiol (76.1 mg, 0.500 mmol), and extra-dry EtOAc (8.0 mL) were added. The flask was filled with oxygen balloon (0.3 MPa) and the reaction mixture was stirred at 70 °C for a duration of 16 h. Subsequently, the reaction mixture was cooled by removing from the oil-bath. The reaction mixture was diluted with EtOAc (10.0 mL) and HCl (15.0 mL, 0.100 M, aq). The aqueous layer was extracted with EtOAc (3 × 15.0 mL). Organic layers were combined, dried over MgSO_4_, filtered, and concentrated afforded 68.1 mg of **2v** in 90% yield as a colorless oil. ^1^H NMR (500 MHz, CDCl_3_) δ 7.47 (m, 4H), 7.20 (m, 4H), 2.91 m, 2H), 1.26 (d, *J* = 7.0 Hz, 12H); ^13^C{^1^H} NMR (126 MHz, CDCl_3_) δ 148.4, 134.4, 128.3, 127.3, 33.8, 24.0.

*1,2-Bis(4-methoxyphenyl)disulfane* (**2w**) [43]. According to the general procedure, the reaction of Sm-OC (9.10 mg, 1.15 mol%) and 4-methoxybenzenethiol (42.1 mg, 0.300 mmol) afforded 40.9 mg of **2w** in 98% yield as a yellow oil. ^1^H NMR (500 MHz, CDCl_3_) δ 7.41 (m, 4H), 6.85 (m, 4H), 3.81 (s, 6H); ^13^C{^1^H} NMR (126 MHz, CDCl_3_) δ 160.0, 132.7, 128.5, 114.7, 55.4.

*1,2-Bis(2-methoxybenzyl)disulfane* (**2x**) [43]. According to the general procedure, the reaction of Sm-OC (9.10 mg, 1.15 mol%) and (2-methoxyphenyl)methanethiol (42.1 mg, 0.300 mmol) afforded 40.9 mg of **2x** in 98% yield as a white solid: ^1^H NMR (500 MHz, CDCl_3_) δ 7.55 (m, 2H), 7.20 (m, 2H), 6.93 (m, 2H), 6.87 (m, 2H), 3.91 (s, 6H); ^13^C{^1^H} NMR (126 MHz, CDCl_3_) δ 156.6, 127.8, 127.6, 124.6, 121.4, 110.5, 55.9.

*1,2-Bis(3,4-dimethoxyphenyl)disulfane* (**2y**) [43]. According to the general procedure, the reaction of Sm-OC (9.10 mg, 1.15 mol%) and 3,4-dimethoxybenzenethiol (51.1 mg, 0.300 mmol) afforded 49.8 mg of **2y** in 98% yield as a white solid. ^1^H NMR (500 MHz, CDCl_3_) δ 7.06 (d, *J* = 2.1 Hz, 1H), 7.04 (d, *J* = 2.1 Hz, 1H), 7.01 (m, 2H), 6.79 (s, 1H), 6.78 (s, 1H), 3.87 (s, 6H), 3.83 (s, 6H); ^13^C{^1^H} NMR (126 MHz, CDCl_3_) δ 149.6, 149.2, 128.7, 123.9, 114.1, 111.3, 56.0 (×2).

*4,4′-Disulfanediyldianiline* (**2z**) [40]. To a round-bottom flask in oil-bath, Sm-OC (9.10 mg, 1.15 mol%), 4-aminobenzenethiol (37.6 mg, 0.300 mmol), and extra-dry EtOAc (8.0 mL) were added. The flask was filled with oxygen balloon (0.3 MPa) and the reaction mixture was stirred at 70 °C for a duration of 16 h. Subsequently, the reaction mixture was cooled by removing from the oil-bath. The reaction mixture was diluted with EtOAc (10.0 mL) and washed with NaOH solution (15.0 mL, 1.00 M, aq). The aqueous layer was extracted with EtOAc (3 × 15.0 mL). Organic layers were combined, dried over MgSO_4_, filtered, and concentratedd afforded 36.5 mg of **2z** in 98% yield as a yellow solid. ^1^H NMR (500 MHz, CDCl_3_) δ 7.16 (m, 4H), 6.47 (m, 4H), 3.69 (s, 4H); ^13^C{^1^H} NMR (126 MHz, CDCl_3_) δ 147.2, 133.9, 125.5, 115.4.

*2,2′-Disulfanediyldianiline* (**2aa**) [40]. To a round-bottom flask in oil-bath, Sm-OC (9.10 mg, 1.15 mol%), 2-aminobenzenethiol (37.6 mg, 0.300 mmol), and extra-dry EtOAc (8.0 mL) were added. The flask was filled with oxygen balloon (0.3 MPa) and the reaction mixture was stirred at 70 °C for a duration of 16 h. Subsequently, the reaction mixture was cooled by removing from the oil-bath. The reaction mixture was diluted with EtOAc (10.0 mL) and washed with NaOH solution (15.0 mL, 1.00 M, aq). The aqueous layer was extracted with EtOAc (3 × 15.0 mL). Organic layers were combined, dried over MgSO_4_, filtered, and concentrated, affording 36.5 mg of **2aa** in 98% yield as a yellow solid. ^1^H NMR (500 MHz, CDCl_3_) δ 7.22 –7.13 (m, 4H), 6.72 (m, 2H), 6.60 (m, 2H), 4.35 (s, 4H); ^13^C{^1^H} NMR (126 MHz, CDCl_3_) δ 148.7, 136.8, 131.6, 118.8, 118.3, 115.3.

*4,4′-Disulfanediyldiphenol* (**2ab**) [47]. According to the general procedure, the reaction of Sm-OC (9.10 mg, 1.15 mol%) and 4-mercaptophenol (37.9 mg, 0.300 mmol) afforded 36.8 mg of **2ab** in 98% yield as a yellow solid. ^1^H NMR (500 MHz, DMSO-*d*_6_) δ 9.85 (s, 2H), 7.28 (m, 4H), 6.77 (m, 4H); ^13^C{^1^H} NMR (126 MHz, DMSO-*d*_6_) δ 158.3, 133.0, 125.2, 116.3.

*3,3′-Disulfanediyldiphenol* (**2ac**) [48]. To a round-bottom flask in oil-bath, Sm-OC (9.10 mg, 1.15 mol%), 3-mercaptophenol (37.9 mg, 0.300 mmol), and extra-dry EtOAc (8.0 mL) were added. The flask was filled with oxygen balloon (0.3 MPa) and the reaction mixture was stirred at 70 °C for a duration of 16 h. Subsequently, the reaction mixture was cooled by removing from the oil-bath. The reaction mixture was diluted with EtOAc (10.0 mL) and HCl (15.0 mL, 0.100 M, aq). The aqueous layer was extracted with EtOAc (3 × 15.0 mL). Organic layers were combined, dried over MgSO_4_, filtered, and concentrated, after chromatography (25–50% EtOAc/Hexane), affording 33.8 mg of **2ac** in 90% yield as a white solid. ^1^H NMR (500 MHz, DMSO-*d*_6_) δ 9.77 (s, 2H), 7.18 (m, 2H), 6.96–6.88 (m, 4H), 6.67 (m, 2H); ^13^C{^1^H} NMR (126 MHz, DMSO-*d*_6_) δ 158.1, 136.7, 130.3, 117.2, 114.6, 113.0.

*1,2-Bis(furan-2-ylmethyl)disulfane* (**2ad**) [42]. To a round-bottom flask in oil-bath, Sm-OC (9.10 mg, 1.15 mol%), thiophene-2-thiol (57.1 mg, 0.500 mmol), and extra-dry EtOAc (8.0 mL) were added. The flask was filled with oxygen balloon (0.3 MPa) and the reaction mixture was stirred at 70 °C for a duration of 16 h. Subsequently, the reaction mixture was cooled by removing from the oil-bath. The reaction mixture was diluted with EtOAc (10.0 mL) and HCl (15.0 mL, 0.100 M, aq). The aqueous layer was extracted with EtOAc (3 × 15.0 mL). Organic layers were combined, dried over MgSO_4_, filtered, and concentrated, after chromatography (0–12.5% EtOAc/Hexane), affording 53.7 mg of **2ad** in 95% yield as a colorless oil. ^1^H NMR (500 MHz, CDCl_3_) δ 7.40 (m, 2H), 6.35 (m, 2H), 6.24 (m, 2H), 3.70 (s, 4H); ^13^C{^1^H} NMR (126 MHz, CDCl_3_) δ 150.3, 142.5, 110.8, 109.0, 35.7.

*1,2-Bis(2-methylfuran-3-yl)disulfane* (**2ae**) [49]. To a round-bottom flask in oil-bath, Sm-OC (9.10 mg, 1.15 mol%), 2-methylfuran-3-thiol (34.2 mg, 0.300 mmol), and extra-dry EtOAc (8.0 mL) were added. The flask was filled with oxygen balloon (0.3 MPa) and the reaction mixture was stirred at 70 °C for a duration of 16 h. Subsequently, the reaction mixture was cooled by removing from the oil-bath. The reaction mixture was diluted with Hexane (10.0 mL) and NaOH (15 mL, 1.00 M, aq). The aqueous layer was extracted with Hexane (3 × 15.0 mL). Organic layers were combined, dried over MgSO_4_, filtered, and concentrated, affording 30.2 mg of **2ae** in 89% yield as a colorless oil. ^1^H NMR (500 MHz, CDCl_3_) δ 7.27 (m, 2H), 6.38 (m, 2H), 2.10 (m, 6H); ^13^C{^1^H} NMR (126 MHz, CDCl_3_) δ 157.1, 140.9, 114.8, 112.8, 11.5.

*1,2-Di(thiophen-2-yl)disulfane* (**2af**) [41]. To a round-bottom flask in oil-bath, Sm-OC (9.10 mg, 1.15 mol%), thiophene-2-thiol (58.1 mg, 0.500 mmol), and extra-dry EtOAc (8.0 mL) were added. The flask was filled with oxygen balloon (0.3 MPa) and the reaction mixture was stirred at 70 °C for a duration of 16 h. Subsequently, the reaction mixture was cooled by removing from the oil-bath. The reaction mixture was diluted with EtOAc (10.0 mL) and HCl (15 mL, 0.100 M, aq). The aqueous layer was extracted with EtOAc (3 × 15.0 mL). Organic layers were combined, dried over MgSO_4_, filtered, and concentrated, after chromatography (100% Hexane), affording 51.8 mg of **2af** in 90% yield as a colorless oil. ^1^H NMR (500 MHz, CDCl_3_) δ 7.51 (dd, *J* = 5.3, 1.4 Hz, 2H), 7.17 (dd, *J* = 3.7, 1.4 Hz, 2H), 7.02 (dd, *J* = 5.3, 3.7 Hz, 2H); ^13^C{^1^H} NMR (126 MHz, CDCl_3_) δ 135.8, 135.7, 132.3, 127.8.

*Methyl5-(1,2-dithiolan-3-yl)pentanoate* (**2ag**) [50]. To a round-bottom flask in oil-bath, Sm-OC (18.2 mg, 2.30 mol%), methyl -6,8-dimercaptooctanoate (66.7 mg, 0.300 mmol), and extra-dry EtOAc (16.0 mL) were added. The flask was filled with oxygen balloon (0.3 MPa) and the reaction mixture was stirred at 70 °C for a duration of 16 h. Subsequently, the reaction mixture was cooled by removing from the oil-bath. The reaction mixture was diluted with EtOAc (10.0 mL) and HCl (15.0 mL, 0.100 M, aq). The aqueous layer was extracted with EtOAc (3 × 15.0 mL). Organic layers were combined, dried over MgSO_4_, filtered, and concentrated, after chromatography (0–12.5% EtOAc/Hexane), affording 34.4 mg of **2ag** in 52% yield as a yellow oil. ^1^H NMR (500 MHz, CDCl_3_) δ 3.68 (s, 3H), 3.57 (m, 1H), 3.19 (m, 1H), 3.12 (m, 1H), 2.47 (m, 1H), 2.33 (t, *J* = 7.5 Hz, 2H), 1.92 (m, 1H), 1.72–1.64 (m, 4H), 1.52–1.44 (m, 2H); ^13^C{^1^H} NMR (126 MHz, CDCl_3_) δ 174.0, 56.4, 51.6, 40.3, 38.6, 34.7, 33.9, 28.8, 24.7.

*(4R,5R)-1,2-dithiane-4,5-diol* (**2ah**) [51]. To a round-bottom flask in oil-bath, Sm-OC (9.10 mg, 1.15 mol%), (2*R*,3*R*)-1,4-dimercaptobutane-2,3-diol (46.3 mg, 0.300 mmol), and extra-dry EtOAc (8.0 mL) were added. The flask was filled with oxygen balloon (0.3 MPa) and the reaction mixture was stirred at 70 °C for a duration of 16 h. Subsequently, the reaction mixture was cooled by removing from the oil-bath. The reaction mixture was diluted with EtOAc (10.0 mL) and HCl (15 mL, 0.100 M, aq). The aqueous layer was extracted with EtOAc (3 × 15.0 mL). Organic layers were combined, dried over MgSO_4_, filtered, and concentrated, after chromatography (0–10% EtOAc/Hexane), affording 26.9 mg of **2ah** in 59% yield as a white solid. ^1^H NMR (500 MHz, Methanol-*d*_4_) δ 3.58–3.42 (m, 2H), 3.12–2.96 (m, 2H), 2.93–2.84 (m, 2H); ^13^C{^1^H} NMR (126 MHz, Methanol-*d*_4_) δ 75.6, 41.7.

*Dimethyl 3,3′-disulfanediyl(2R,2′R)-bis(2-((tert-butoxycarbonyl)amino)propanoate)* (**2ai**) [21]. To a round-bottom flask in oil-bath, Sm-OC (18.2 mg, 2.30 mol%), methyl (tert-butoxycarbonyl)-*L*-cysteinate (70.6 mg, 0.300 mmol), and extra-dry EtOAc (16.0 mL) were added. The flask was filled with oxygen balloon (0.3 MPa) and the reaction mixture was stirred at 70 °C for a duration of 16 h. Subsequently, the reaction mixture was cooled by removing from the oil-bath. The reaction mixture was diluted with EtOAc (10.0 mL) and HCl (15.0 mL, 0.100 M, aq). The aqueous layer was extracted with EtOAc (3 × 15.0 mL). Organic layers were combined, dried over MgSO_4_, filtered, and concentrated, after chromatography (20–30% EtOAc/Hexane), affording 47.1 mg of **2ai** in 67% yield as a white solid. ^1^H NMR (500 MHz, DMSO-*d*_6_) δ 7.38 (d, *J* = 8.2 Hz, 2H), 4.26 (m, 2H), 3.64 (s, 6H), 3.08 (m, 2H), 2.90 (m, 2H), 1.38 (s, 18H); ^13^C{^1^H} NMR (126 MHz, DMSO-*d*_6_) δ 171.4, 155.3, 78.5, 52.7, 52.1, 39.1, 28.1.

*(2R,2′R)-3,3′-disulfanediylbis(2-acetamidopropanoic acid)* (**2aj**) [52]. To a round-bottom flask in oil-bath, Sm-OC (18.2 mg, 2.30 mol%), acetyl-*L*-cysteine (49.3 mg, 0.300 mmol), and extra-dry EtOAc (16.0 mL) were added. The flask was filled with oxygen balloon (0.3 MPa) and the reaction mixture was stirred at 70 °C for a duration of 16 h. Subsequently, the reaction mixture was cooled by removing from the oil-bath. The reaction mixture was diluted with EtOAc (10.0 mL) and HCl (15.0 mL, 0.100 M, aq). The aqueous layer was extracted with EtOAc (3 × 15.0 mL). Organic layers were combined, dried over MgSO_4_, filtered, and concentrated, affording 40.0 mg of **2aj** in 82% yield as a white solid. ^1^H NMR (500 MHz, D_2_O) δ 4.68 (dd, *J* = 8.6, 4.3 Hz, 2H), 3.38 (dd, *J* = 14.1, 4.3 Hz, 2H), 3.03 (dd, *J* = 14.1, 8.6 Hz, 2H), 2.04 (s, 6H); ^13^C{^1^H} NMR (126 MHz, D_2_O) δ 177.7, 174.1, 53.2, 39.4, 21.8. HRMS (ESI-TOF) calcd. For C_10_H_16_N_2_O_6_S_2_ [M + H]^+^ 325.0522, found: 325.0521.

*1-((3s,5s,7s)-adamantan-1-yl)-2-dodecyldisulfane* (**2ak**) [20]. To a round-bottom flask in oil-bath, Sm-OC (9.10 mg, 1.15 mol%), dodecane-1-thiol (40.5 mg, 0.200 mmol), (3*s*,5*s*,7*s*)-adamantane-1-thiol (101.0 mg, 0.600 mmol), and extra-dry EtOAc (8.0 mL) were added. The flask was filled with oxygen balloon (0.3 MPa) and the reaction mixture was stirred at 70 °C for a duration of 16 h. Subsequently, the reaction mixture was cooled by removing from the oil-bath. The reaction mixture was diluted with EtOAc (10.0 mL) and HCl (15.0 mL, 0.100 M, aq). The aqueous layer was extracted with EtOAc (3 × 15.0 mL). Organic layers were combined, dried over MgSO_4_, filtered, and concentrated, after chromatography (100% Hexane), affording 57.5mg of **2ak** in 78% yield as a colorless oil. ^1^H NMR (500 MHz, CDCl_3_) δ 2.67 (t, *J* = 7.0 Hz, 2H), 2.12–2.03 (m, 3H), 1.89–1.82 (m, 6H), 1.74–1.61 (m, 8H), 1.42–1.23 (m, 18H), 0.89 (t, *J* = 7.0 Hz, 3H); ^13^C{^1^H} NMR (126 MHz, CDCl_3_) δ 49.3, 42.7, 41.6, 36.3, 32.0, 29.9, 29.7(×3), 29.6, 29.4(×2), 29.3, 28.7, 22.8, 14.2.

*1-((3s,5s,7s)-adamantan-1-yl)-2-(p-tolyl)disulfane* (**2al**) [53]. To a round-bottom flask in oil-bath, Sm-OC (9.10 mg, 1.15 mol%), 4-methylbenzenethiol (24.8 mg, 0.200 mmol), (3*s*,5*s*,7*s*)-adamantane-1-thiol (101.0 mg, 0.600 mmol), and extra-dry EtOAc (8.0 mL) were added. The flask was filled with oxygen balloon (0.3 MPa) and the reaction mixture was stirred at 70 °C for a duration of 16 h. Subsequently, the reaction mixture was cooled by removing from the oil-bath. The reaction mixture was diluted with EtOAc (10.0 mL) and HCl (15.0 mL, 0.100 M, aq). The aqueous layer was extracted with EtOAc (3 × 15.0 mL). Organic layers were combined, dried over MgSO_4_, filtered, and concentrated, after chromatography (100% Hexane), affording 43.6 mg of **2al** in 75% yield as a white solid. ^1^H NMR (500 MHz, CDCl_3_) δ 7.46 (m, 2H), 7.11 (m, 2H), 2.33 (s, 3H), 2.06–2.02 (m, 3H), 1.87–1.84 (m, 6H), 1.68–1.62 (m, 6H); ^13^C NMR{^1^H} (126 MHz, CDCl_3_) δ 136.1, 135.9, 129.5, 127.1, 50.7, 42.6, 36.2, 30.0, 21.1.

### 3.5. Procedure of Control Experiments (Table 2)

Table 2, entry 1: To a round-bottom flask in oil-bath, dodecane-1-thiol (60.7 mg, 0.300 mmol, 1.00 equiv.) and extra-dry EtOAc (8.00 mL) were added. The reaction mixture was stirred under an oxygen atmosphere at 70 °C for a period of 16 h. Subsequently, the reaction mixture was cooled by removing from the oil-bath. The reaction mixture was diluted with EtOAc (10.0 mL) and washed with HCl solution (15.0 mL, 0.100 M, aq). After phase separation, the aqueous layer was extracted with EtOAc (3 × 15.0 mL). Organic layers were combined, dried over MgSO_4_, filtered, and concentrated. The crude product was analyzed by ^1^H NMR (CDCl_3_, 500 MHz) using internal standard (1,1,2,2-tetrachloroethane).

Table 2, entry 2: To a round-bottom flask in oil-bath, *n*-Bu_4_NI (1.00 mol%), dodecane-1-thiol (60.7 mg, 0.300 mmol, 1.00 equiv.), and extra-dry EtOAc (8.00 mL) were added. The reaction mixture was stirred under an oxygen atmosphere at 70 °C for a period of 16 h. Subsequently, the reaction mixture was cooled by removing from the oil-bath. The reaction mixture was diluted with EtOAc (10.0 mL) and washed with HCl solution (15.0 mL, 0.100 M, aq). After phase separation, the aqueous layer was extracted with EtOAc (3 × 15.0 mL). Organic layers were combined, dried over MgSO_4_, filtered, and concentrated. The crude product was analyzed by ^1^H NMR (CDCl_3_, 500 MHz) using internal standard (1,1,2,2-tetrachloroethane).

Table 2, entry 3: To a round-bottom flask in oil-bath, NaI (1.00 mol%), dodecane-1-thiol (60.7 mg, 0.300 mmol, 1.00 equiv.), and extra-dry EtOAc (8.00 mL) were added. The reaction mixture was stirred under an oxygen atmosphere at 70 °C for a period of 16 h. Subsequently, the reaction mixture was cooled by removing from the oil-bath. The reaction mixture was diluted with EtOAc (10.0 mL) and washed with HCl solution (15.0 mL, 0.100 M, aq). After phase separation, the aqueous layer was extracted with EtOAc (3 × 15.0 mL). Organic layers were combined, dried over MgSO_4_, filtered, and concentrated. The crude product was analyzed by ^1^H NMR (CDCl_3_, 500 MHz) using internal standard (1,1,2,2-tetrachloroethane).

Table 2, entry 4: To a round-bottom flask in oil-bath, SmCl_3_ (300 mol%), dodecane-1-thiol (60.7 mg, 0.300 mmol, 1.00 equiv.), and extra-dry EtOAc (8.00 mL) were added. The reaction mixture was stirred under an oxygen atmosphere at 70 °C for a period of 16 h. Subsequently, the reaction mixture was cooled by removing from the oil-bath. The reaction mixture was diluted with EtOAc (10.0 mL) and washed with HCl solution (15.0 mL, 0.100 M, aq). After phase separation, the aqueous layer was extracted with EtOAc (3 × 15.0 mL). Organic layers were combined, dried over MgSO_4_, filtered, and concentrated. The crude product was analyzed by ^1^H NMR (CDCl_3_, 500 MHz) using internal standard (1,1,2,2-tetrachloroethane).

Table 2, entry 5: To a round-bottom flask in oil-bath, Sm_2_O_3_ (300 mol%), dodecane-1-thiol (60.7 mg, 0.300 mmol, 1.00 equiv.), and extra-dry EtOAc (8.00 mL) were added. The reaction mixture was stirred under an oxygen atmosphere at 70 °C for a period of 16 h. Subsequently, the reaction mixture was cooled by removing from the oil-bath. The reaction mixture was diluted with EtOAc (10.0 mL) and washed with HCl solution (15.0 mL, 0.100 M, aq). After phase separation, the aqueous layer was extracted with EtOAc (3 × 15.0 mL). Organic layers were combined, dried over MgSO_4_, filtered, and concentrated. The crude product was analyzed by ^1^H NMR (CDCl_3_, 500 MHz) using internal standard (1,1,2,2-tetrachloroethane).

### 3.6. Synthesis of Methyl 6,8-Dimercaptooctanoate [54] 


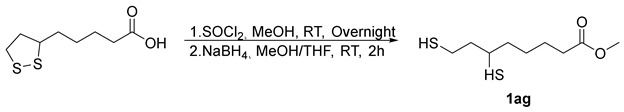


To a solution of α-lipoic acid (2.06 g) in methanol (50.0 mL), SOCl_2_ (2.98 g) was added dropwise under ice bath condition. The resultant reaction solution was stirred at room temperature overnight, and then the reaction mixture was diluted with CH_2_Cl_2_ (40.0 mL) and NaHCO_3_ (30.0 mL, 1.14 M, aq). The organic layer was extracted with NaHCO_3_ (5 × 30.0 mL); organic layer was washed with brine, dried over MgSO_4_, filtered, and concentrated, affording 0.647 g of methyl 5-(1,2-dithiolan-3-yl)pentanoate in 33% yield a yellow oil. The residue was used in the next reaction without further purification. To a solution of the crude methyl ester in THF/MeOH (9:1, 10.0 mL), NaBH_4_ (222 mg) was added under ice bath conditions. After this, the mixture was stirred for 2 h at room temperature. The mixture was diluted with CH_2_Cl_2_ (10.0 mL) and HCl (10.0 mL, 0.100 M, aq) and extracted with CH_2_Cl_2_ (3 × 15.0 mL). The organic layers were combined, dried over MgSO_4_, filtered, and concentrated. The crude product was purified by flash chromatography (silica, 0–12.5% EtOAc/Hexane), affording 50.4 mg of methyl 6,8-dimercaptooctanoate in 77% yield as a colorless oil. ^1^H NMR (500 MHz, CDCl_3_) δ 3.68 (s, 3H), 3.00–2.88 (m, 1H), 2.81–2.61 (m, 2H), 2.33 (t, *J* = 7.4 Hz, 2H), 1.91 (m, 1H), 1.82–1.39 (m, 7H), 1.35 (t, *J* = 8.0 Hz, 1H), 1.31 (d, *J* = 7.6, 1H); ^13^C{^1^H} NMR (126 MHz, CDCl_3_) δ 174.1, 51.6, 42.8, 39.4, 38.8, 34.0, 26.6, 24.6, 22.4.

### 3.7. Recycle Experiment of Sm-OC

To a round-bottom flask in oil-bath, Sm-OC (38.0 mg, 1.20 mol%), 1,2-Bis(4-bromophenyl)disulfane (1.20 mmol, 1.00 equiv.), and extra-dry EtOAc (38.0 mL) were added. The flask was filled with oxygen balloon (0.3 MPa) and the reaction mixture was stirred at 70 °C for a duration of 16 h. Subsequently, the reaction mixture was cooled by removing from the oil-bath. The mixture was concentrated to remove EtOAc. Next, the product was extracted with hexane (50.0 mL) from the sediment and then centrifuged for 10 min at 4000 rpm. The above step was repeated three times. Subsequently, the supernatant was combined, dried over MgSO_4_, filtered, and concentrated to yield the product. Then, the sample was analyzed by ^1^H NMR (CDCl_3_, 300 MHz) to obtain the yield using internal standard (1,1,2,2-tetrachlorethan) and compared with corresponding samples. Finally, the residual trace solvent in the sediment was removed by overnight exposure on the vacuum line, which was used in the next cycle experiment.

## 4. Conclusions

In summary, we established a cost-effective synthesis of disulfides through the aerobic oxidation coupling of thiols catalyzed by Sm-OC. The catalyst system demonstrates broad substrate tolerance with excellent chemoselectivity and recoverability, avoiding the generation of over-oxidized by-products. This catalytic system without an organic ligand exhibits a broad substrate scope, holding potential for the synthesis of complex new materials and pharmaceuticals. This catalytic system serves as a template for the future development of this series for organic catalysis, with promising applications anticipated across academic and industrial domains.

## Figures and Tables

**Table 1:** Aerobic oxidation of **1a** by Sm-OC.

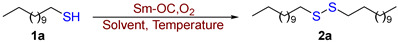					
Entry	Solvent	Catalyst (mol%)	Temperature (°C)	Time (h)	Yield *^a^* (%)
1	AcOEt	10	70	16	>98
2	AcOEt	5	70	16	>98
3	AcOEt	2	70	16	>98
4	AcOEt	1	70	16	>98
5	AcOEt	0.2	70	16	4
6	MeOH	10	rt	16	22
7	EtOH	10	rt	16	26
8	MeCN	10	rt	16	36
9	THF	10	rt	16	44
10	AcOEt	10	rt	16	42
11	AcOEt	1	70	4	28
12	AcOEt	1	70	1	13

*^a^* Determined by ^1^H NMR.

**Table 2:** Control reactions.

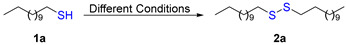			
Entry	Solvent	Catalyst	Yield *^c^* (%)
1	EtOAc	/	10
2	EtOAc	*n*-Bu_4_NI *^a^*	4
3	EtOAc	NaI *^a^*	9
4	EtOAc	SmCl_3_ *^b^*	7
5	EtOAc	Sm_2_O_3_ *^b^*	0

*^a^* Conditions: Catalyst (1.15 mol%) was added to a solution of **1a** (0.300 mmol, 1.00 equiv.) in AcOEt (8.00 mL) under O_2_ balloon at 70 °C and stirred for a duration of 16 h. *^b^* Catalysts (300 mol%) were added to a solution of **1a** (0.300 mmol, 1.00 equiv.) in AcOEt (8.00 mL) under O_2_ balloon at 70 °C and stirred for a duration of 16 h. *^c^* Determined by ^1^H NMR.

## Data Availability

Data are contained within the article and Supplementary Materials.

## Supplementary Materials

The following supporting information can be downloaded at: https://www.mdpi.com/article/10.3390/molecules29143361/s1, includes ^1^H NMR and ^13^C{^1^H} NMR data of **1ag**, **2a**–**2ag**.
