# Supplementary material for: A Recyclable Inorganic Lanthanide Cluster Catalyst for Chemoselective Aerobic Oxidation of Thiols
Source: Molecules. 2024 Jul 17;29(14):3361. doi: 10.3390/molecules29143361 (PMC11279804; doi:10.3390/molecules29143361)
Supplement: Supplementary file 1 [file molecules-29-03361-s001.zip › molecules-3084017-supplementary.pdf]

Supplementary Materials

A Recyclable Inorganic Lanthanide Cluster Catalyst for Chemoselective Aerobic Oxidation of Thiols

Lijun Wang [1,2], Zixuan Qin [1], Lingxia Chen [1], Xinshu Qin [1], Jiaman Hou [1], Chao Wang [1], Xuan Li [1], Hongxia Duan [2], Bing Fang [1,*], Minlong Wang [1,*] and Jie An [1]

[1] Department of Nutrition and Health, China Agricultural University, Beijing, 100193, China; lijunwang@cau.edu.cn (L.W.); shxzq1@nottingham.edu.cn (Z.Q.); lxchen915@163.com (L.C.); qinxs98@163.com (X.Q.); y20223313563@cau.edu.cn (J.H.); B20233311213@cau.edu.cn (C.W.); sy20233313716@cau.edu.cn (X.L.); jie_an@cau.edu.cn (J.A)

[2] Department of Chemistry, College of Science, China Agricultural University, Beijing 100193, China; hxduan@cau.edu.cn

* Correspondence: bingfang@cau.edu.cn (B.F.); mlwang@cau.edu.cn (M.W.)

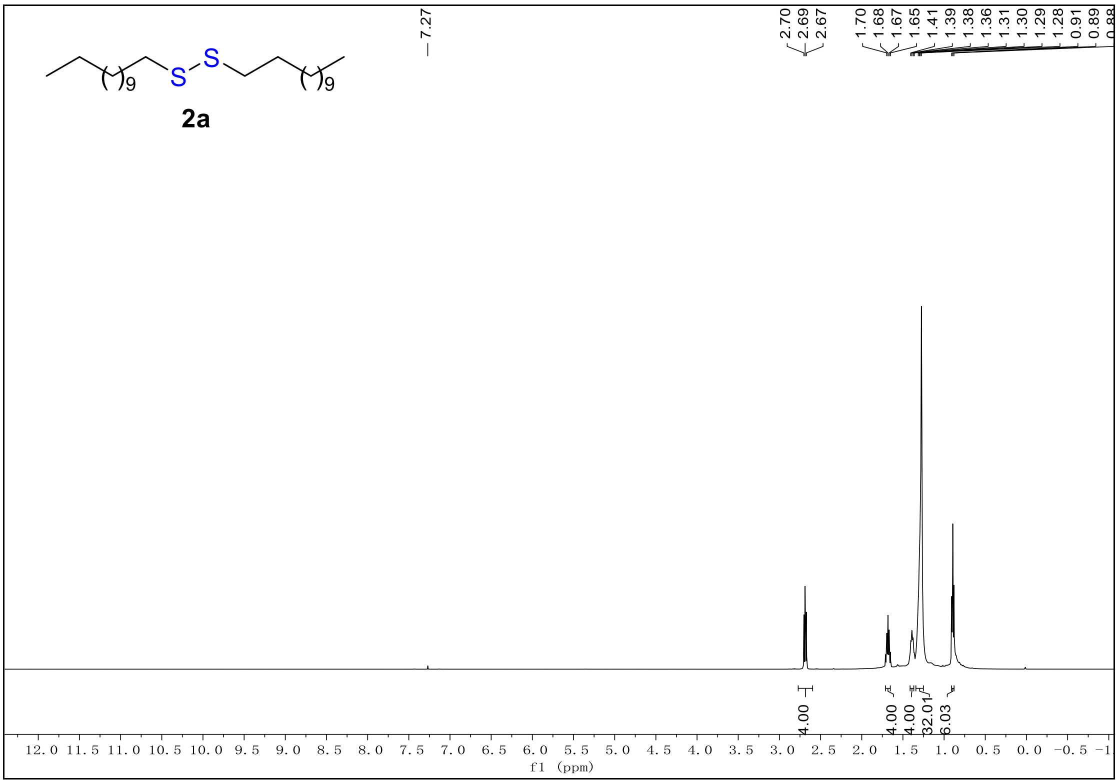
2a
7.27
2.70
2.69
2.67
1.70
1.68
1.67
1.65
1.41
1.39
1.38
1.36
1.31
1.30
1.29
1.28
0.91
0.89
0.88
4.00
4.00
4.00
32.01
6.03
f1 (ppm)

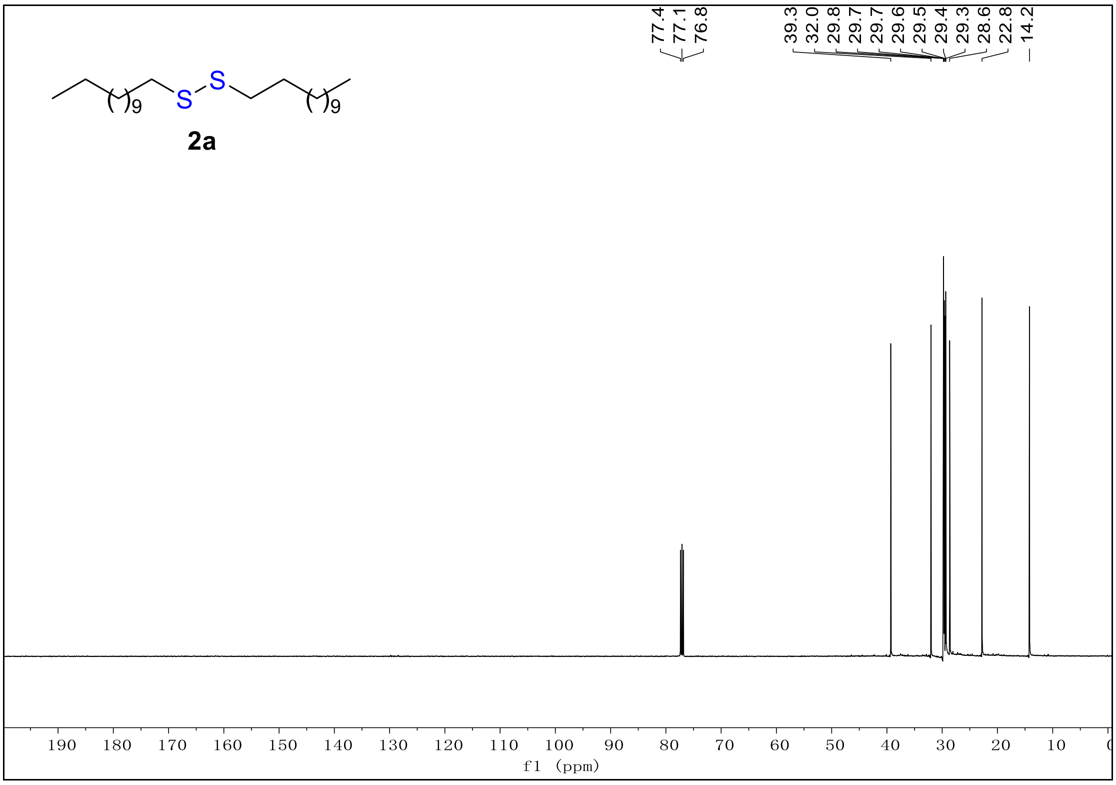
2a
77.4
77.1
76.8
39.3
32.0
29.8
29.7
29.7
29.6
29.5
29.4
29.3
28.6
22.8
14.2
f1 (ppm)

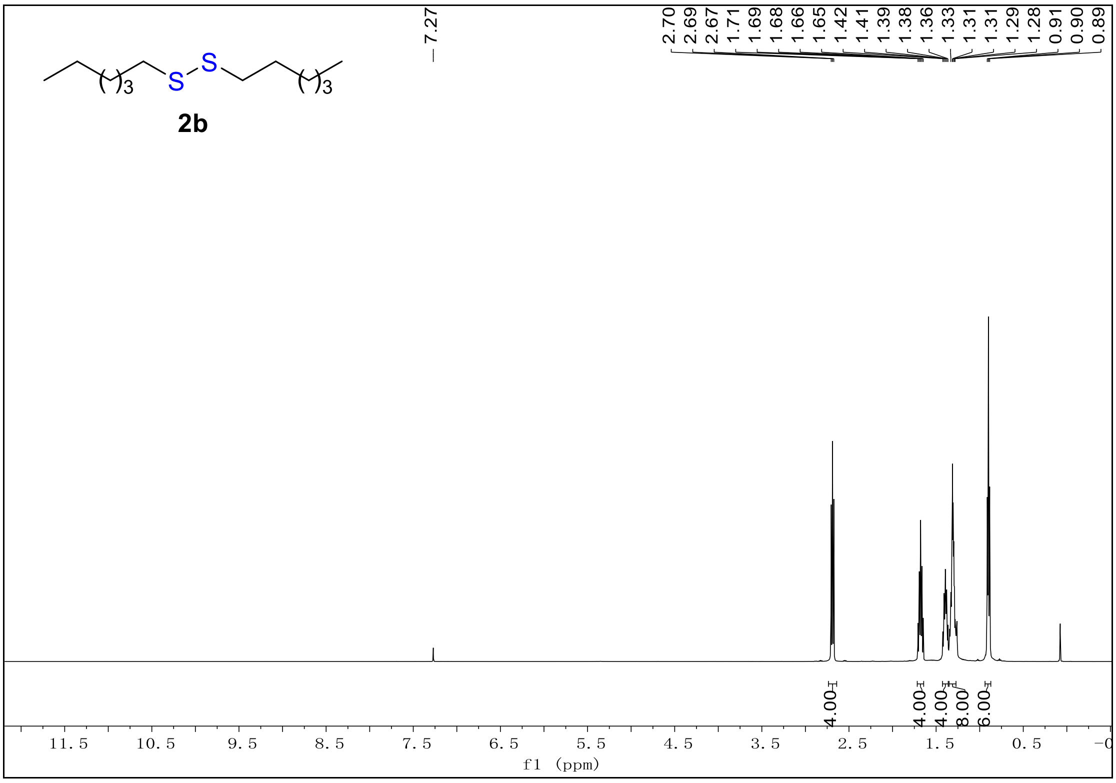
2b
7.27
2.70
2.69
2.67
1.71
1.69
1.68
1.66
1.65
1.42
1.41
1.39
1.38
1.36
1.33
1.31
1.31
1.29
1.28
0.91
0.90
0.89
4.00
4.00
4.00
8.00
6.00
f1 (ppm)

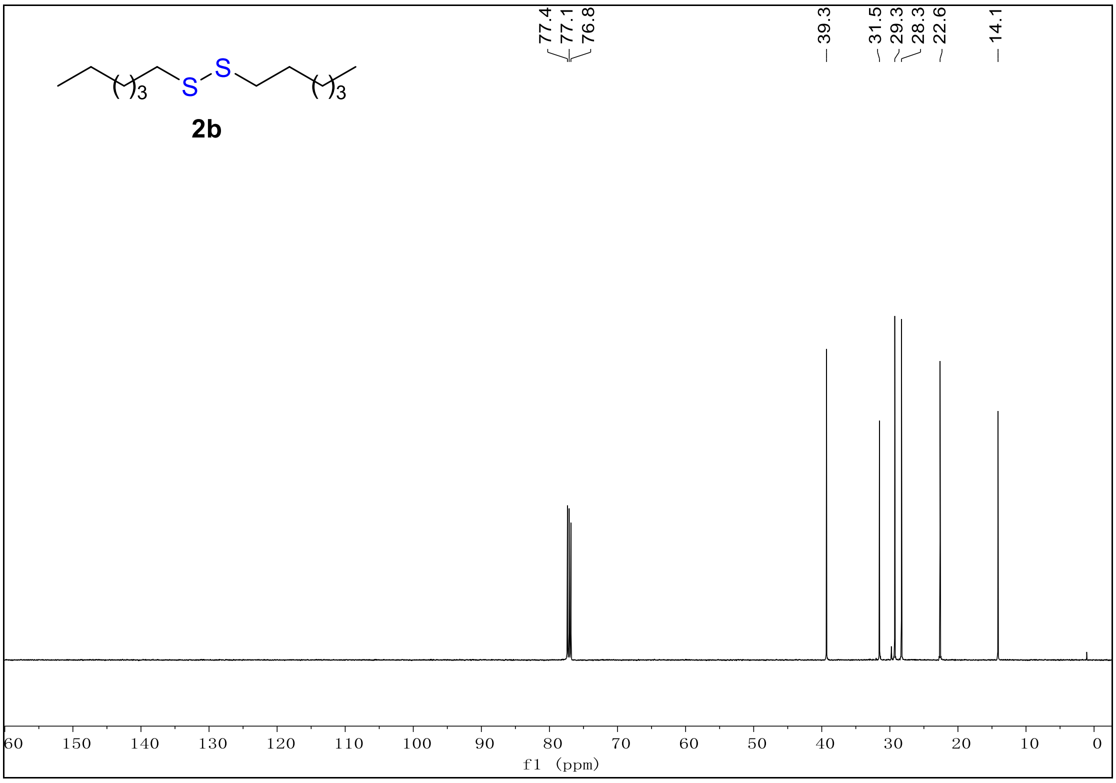
2b
77.4
77.1
76.8
39.3
31.5
29.3
28.3
22.6
14.1
f1 (ppm)

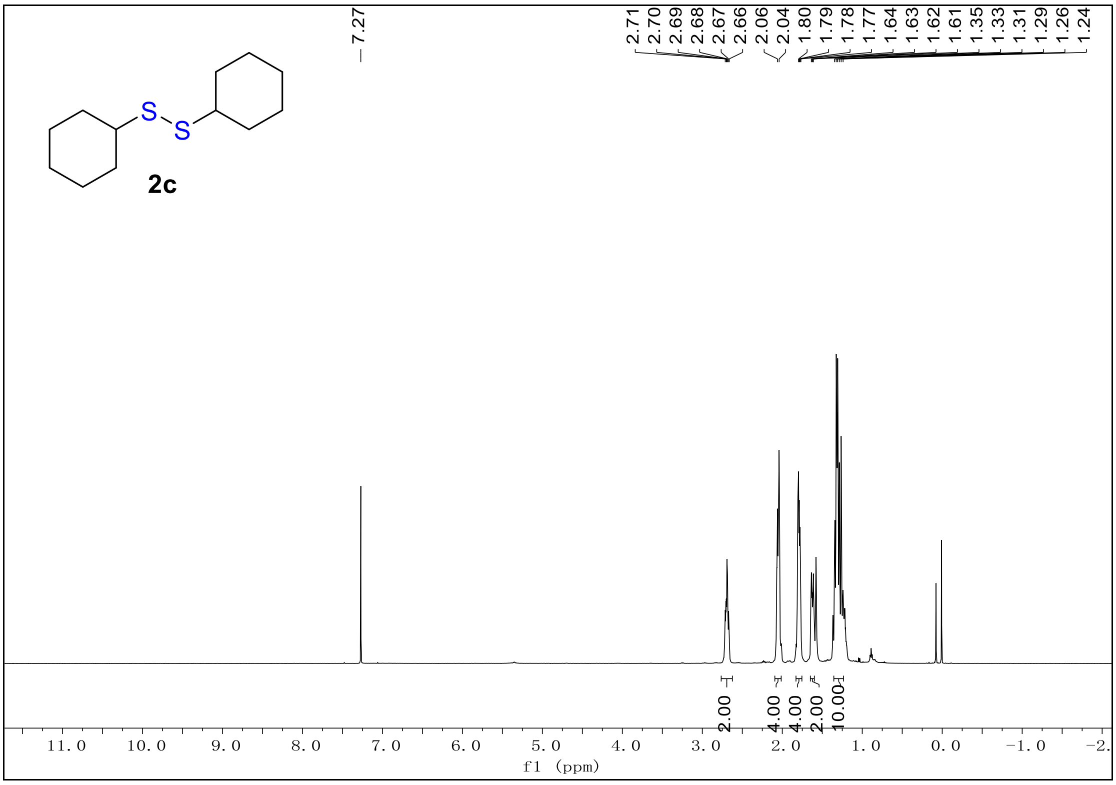
7.27
2.71
2.70
2.69
2.68
2.67
2.66
2.06
2.04
1.80
1.79
1.78
1.77
1.64
1.63
1.62
1.61
1.35
1.33
1.31
1.29
1.26
1.24
2c
2.00
4.00
4.00
2.00
10.00
11.0 10.0 9.0 8.0 7.0 6.0 5.0 4.0 3.0 2.0 1.0 0.0 -1.0
f1 (ppm)

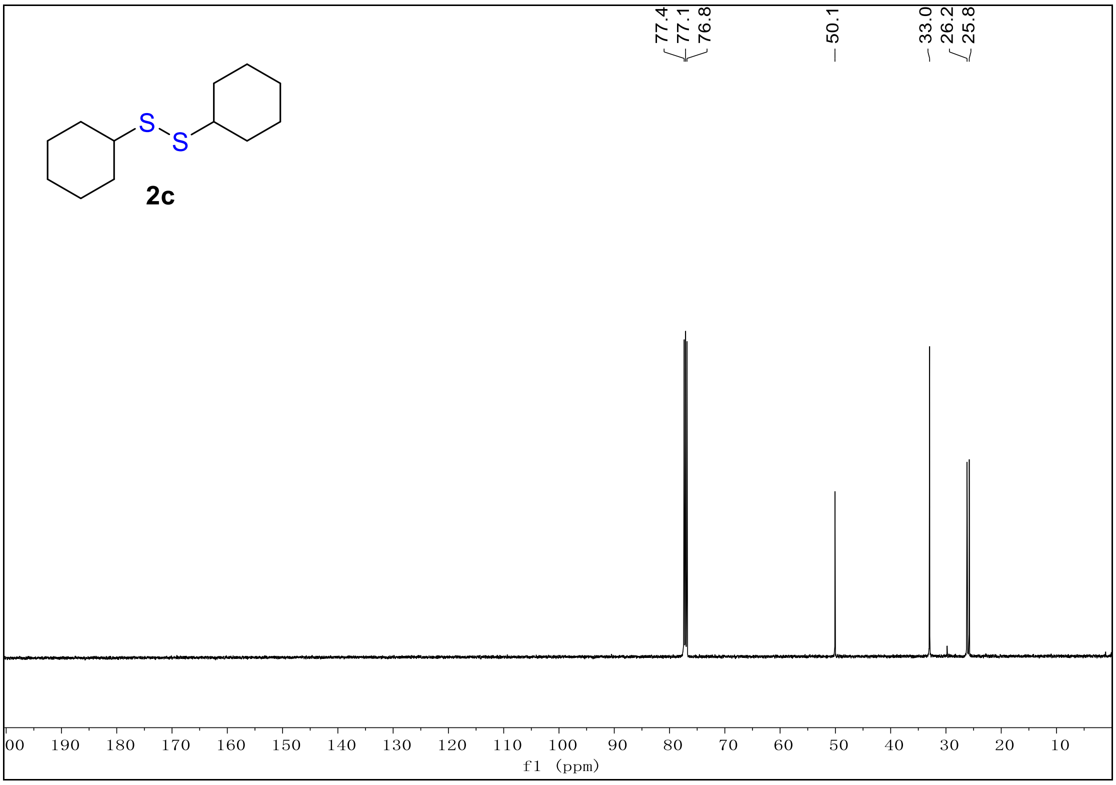
77.4
77.1
76.8
50.1
33.0
26.2
25.8
2c
190 180 170 160 150 140 130 120 110 100 90 80 70 60 50 40 30 20 10
f1 (ppm)

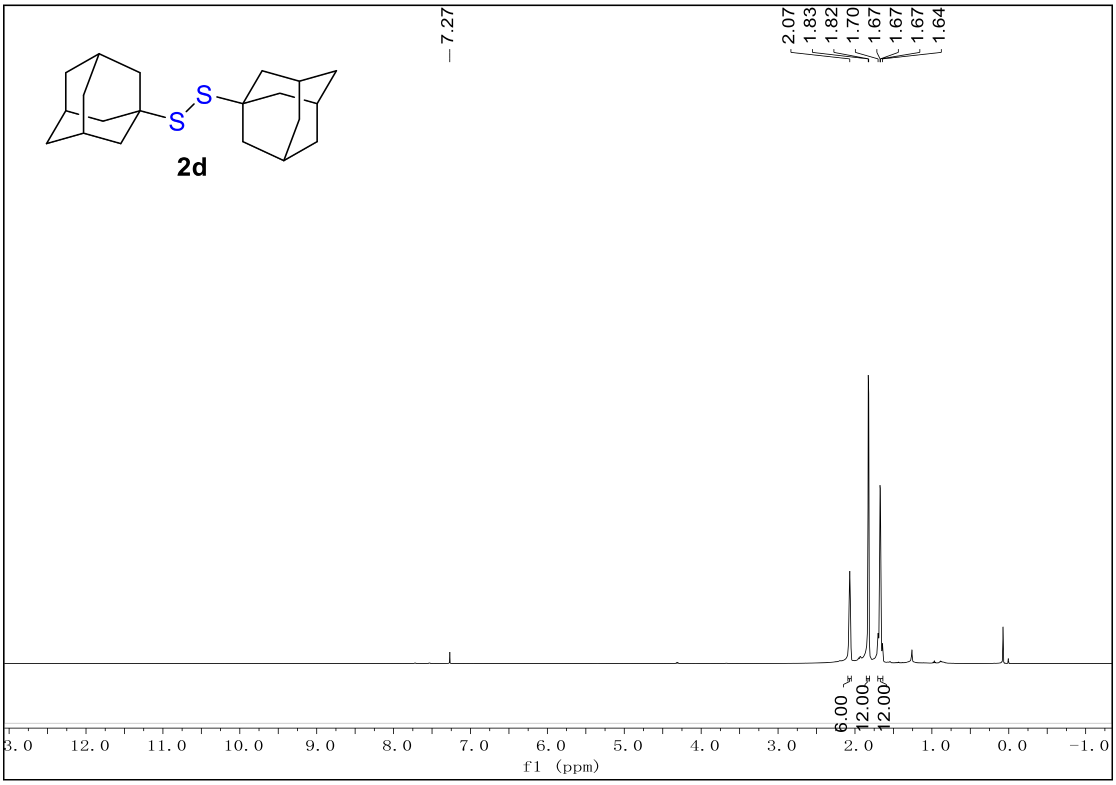
7.27
2.07
1.83
1.82
1.70
1.67
1.67
1.67
1.64
S
S
2d
6.00
12.00
12.00
f1 (ppm)

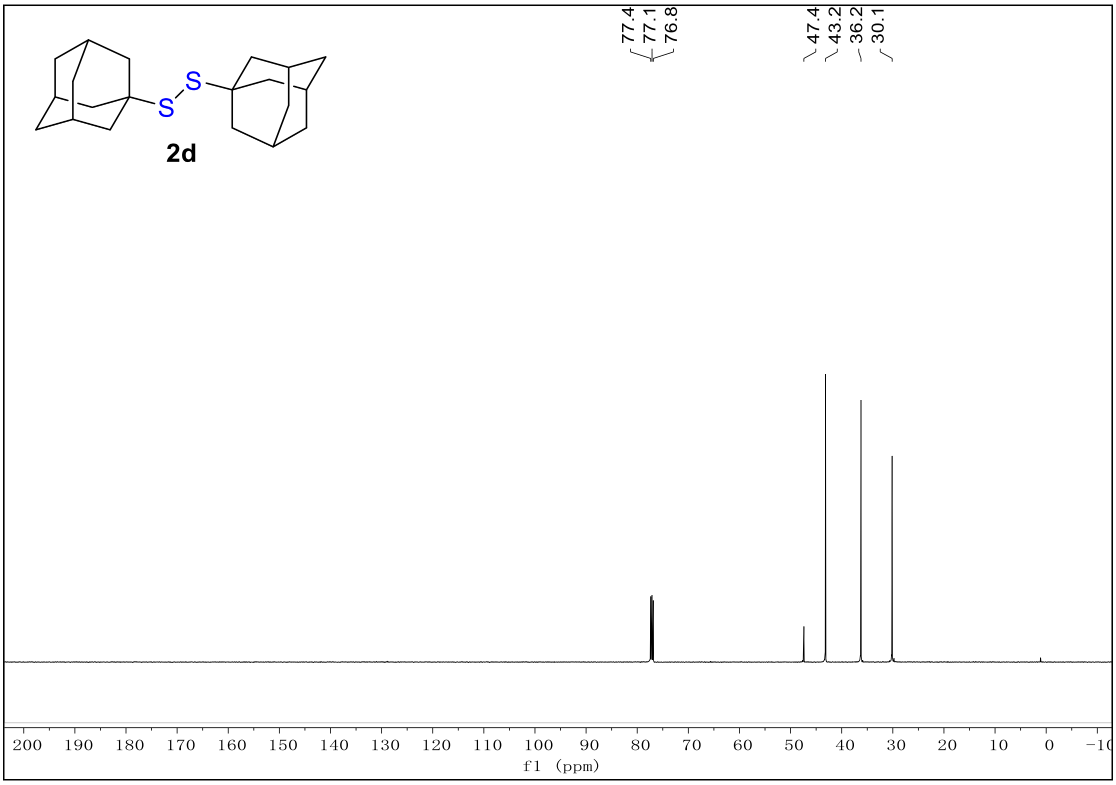
77.4
77.1
76.8
47.4
43.2
36.2
30.1
S
S
2d
f1 (ppm)

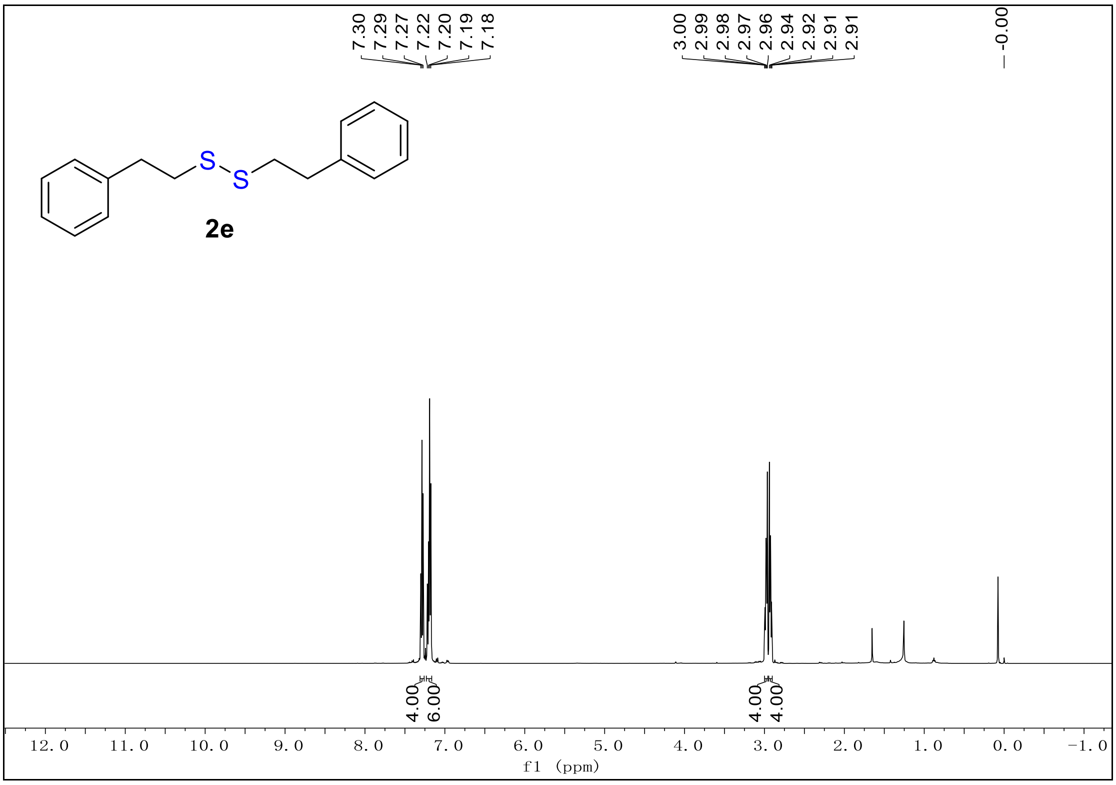
7.30
7.29
7.27
7.22
7.20
7.19
7.18
3.00
2.99
2.98
2.97
2.96
2.94
2.92
2.91
2.91
-0.00
2e
4.00
6.00
4.00
4.00
12.0
11.0
10.0
9.0
8.0
7.0
6.0
5.0
4.0
3.0
2.0
1.0
0.0
-1.0
f1 (ppm)

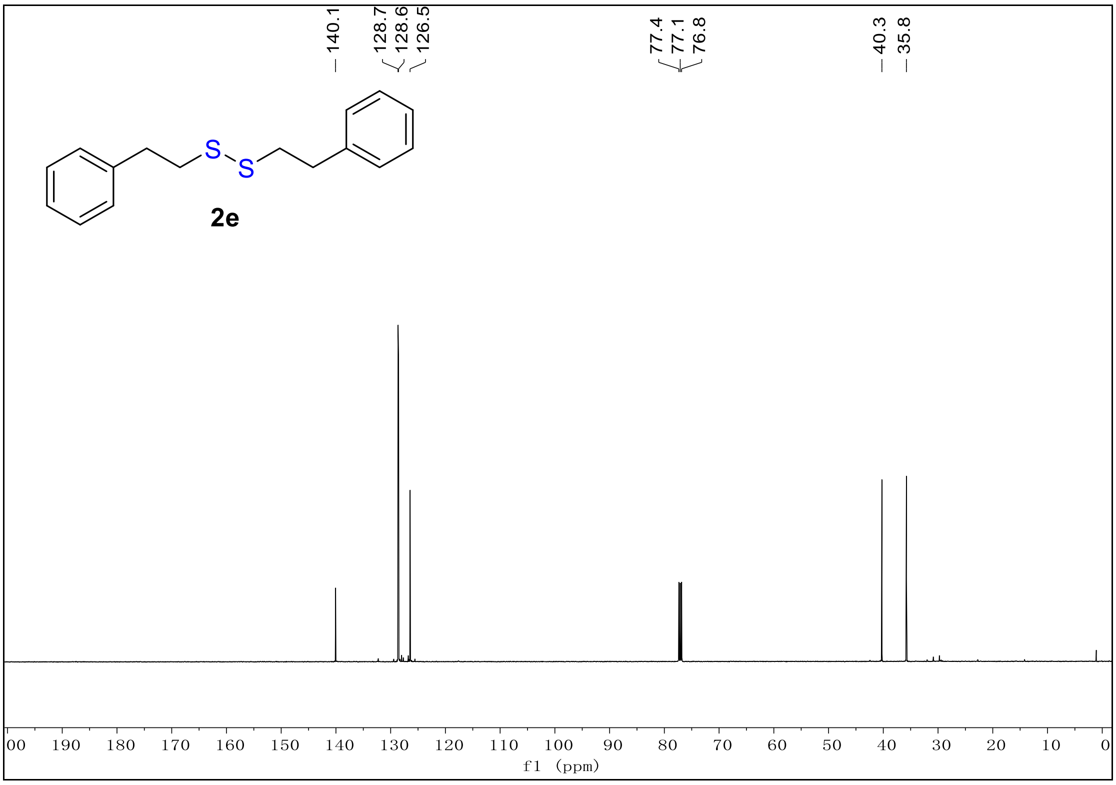
140.1
128.7
128.6
126.5
77.4
77.1
76.8
40.3
35.8
2e
00
190
180
170
160
150
140
130
120
110
100
90
80
70
60
50
40
30
20
10
0
f1 (ppm)

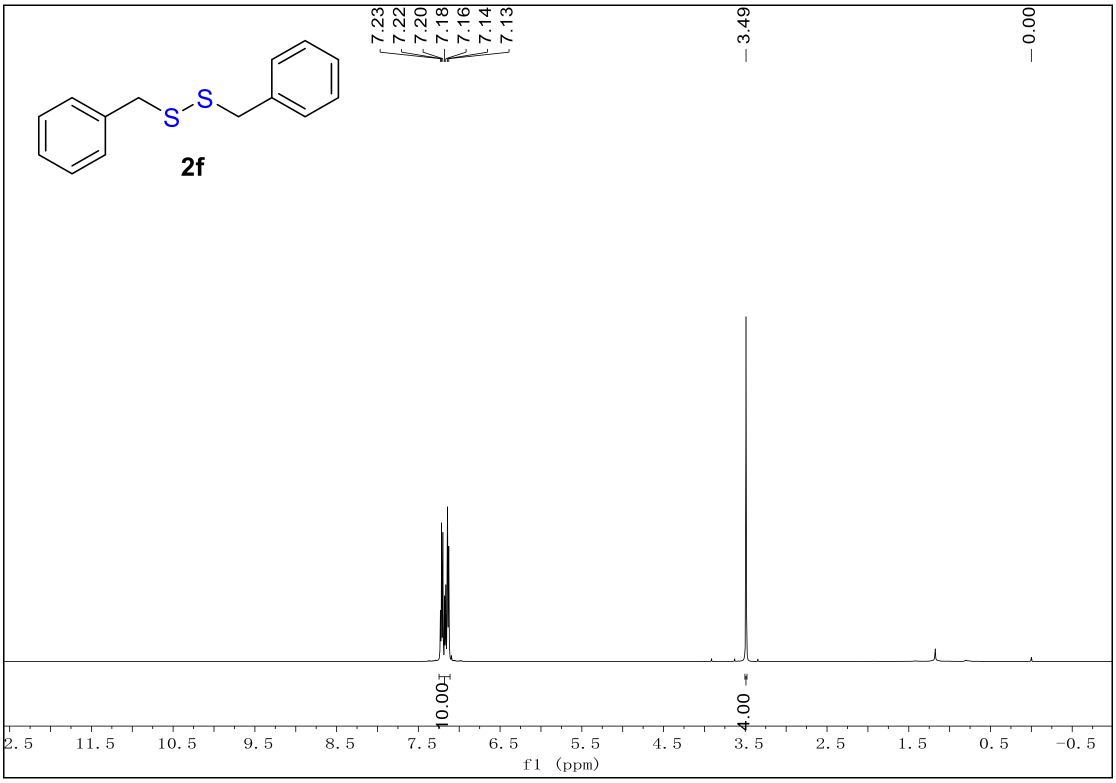
7.23
7.22
7.20
7.18
7.16
7.14
7.13
3.49
0.00
2f
10.00
4.00
f1 (ppm)

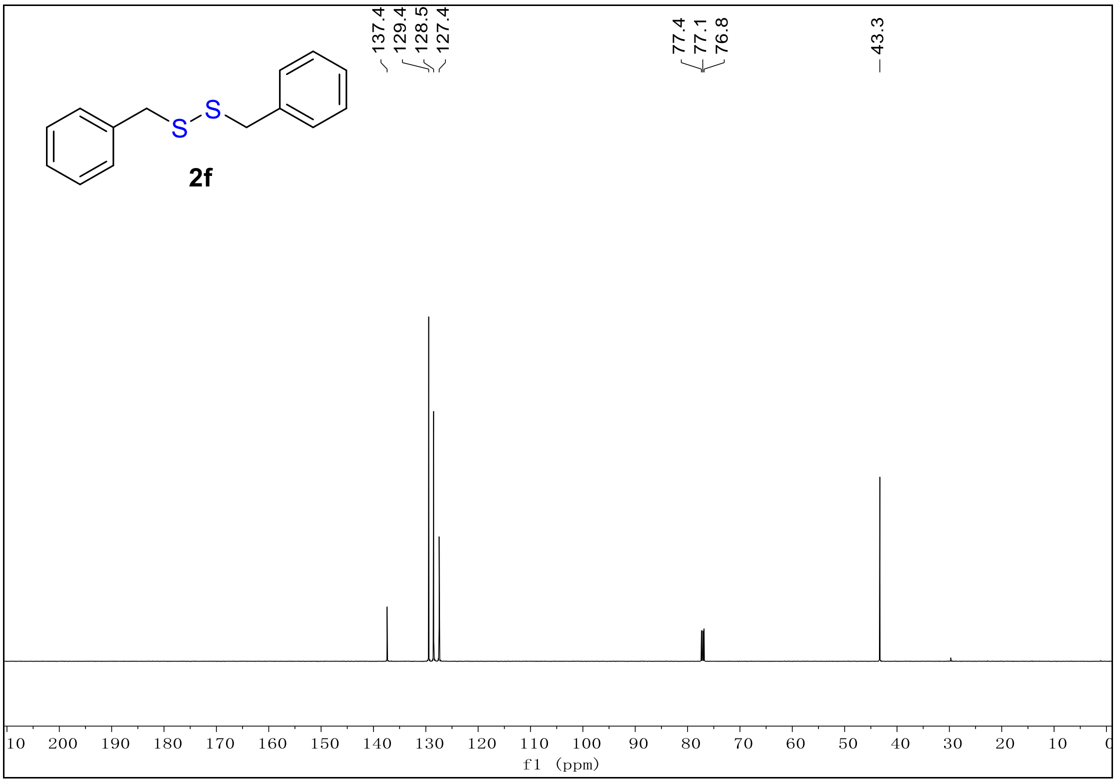
137.4
129.4
128.5
127.4
77.4
77.1
76.8
43.3
2f
f1 (ppm)

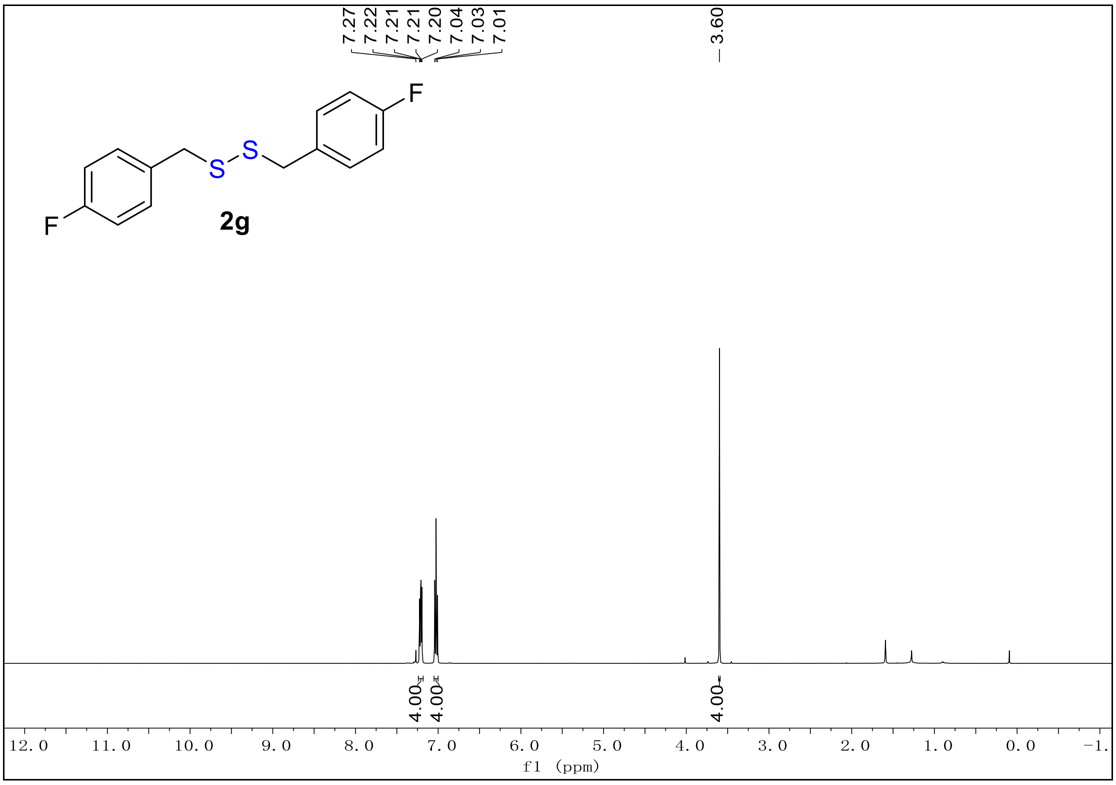
7.27
7.22
7.21
7.21
7.20
7.04
7.03
7.01
3.60
F
S
S
F
2g
4.00
4.00
4.00
12.0 11.0 10.0 9.0 8.0 7.0 6.0 5.0 4.0 3.0 2.0 1.0 0.0 -1.
f1 (ppm)

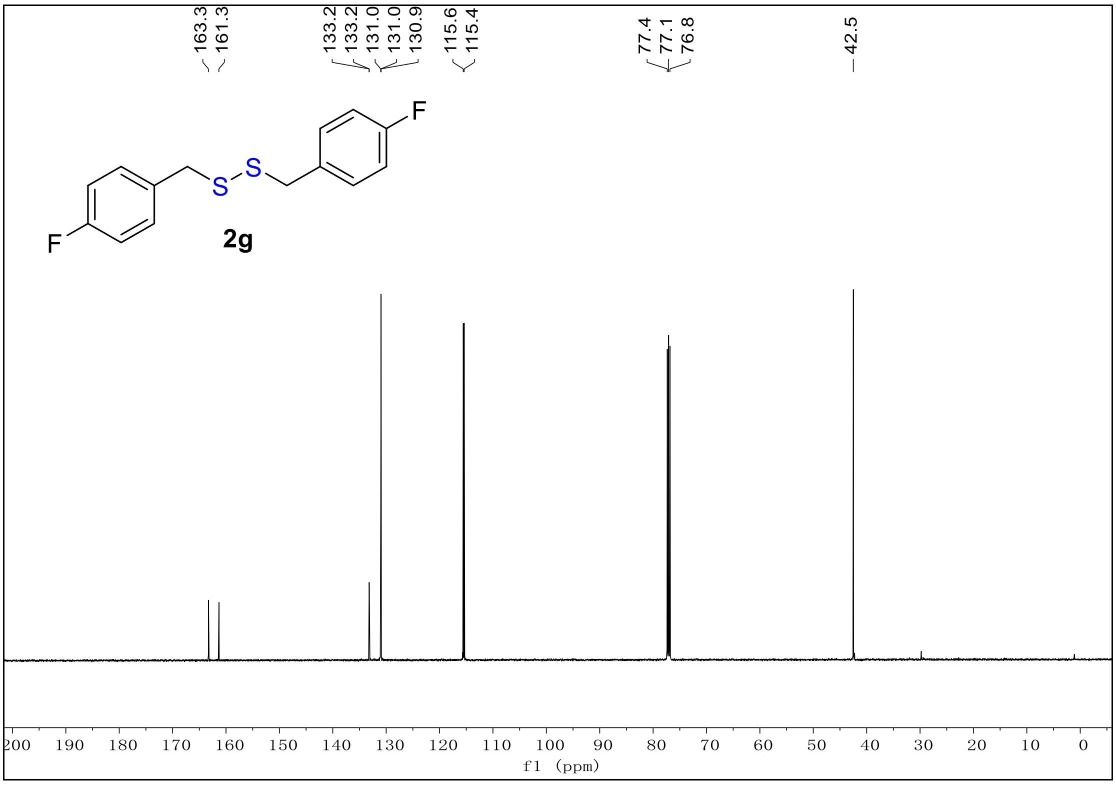
163.3
161.3
133.2
133.2
131.0
131.0
130.9
115.6
115.4
77.4
77.1
76.8
42.5
F
S
S
F
2g
200 190 180 170 160 150 140 130 120 110 100 90 80 70 60 50 40 30 20 10 0
f1 (ppm)

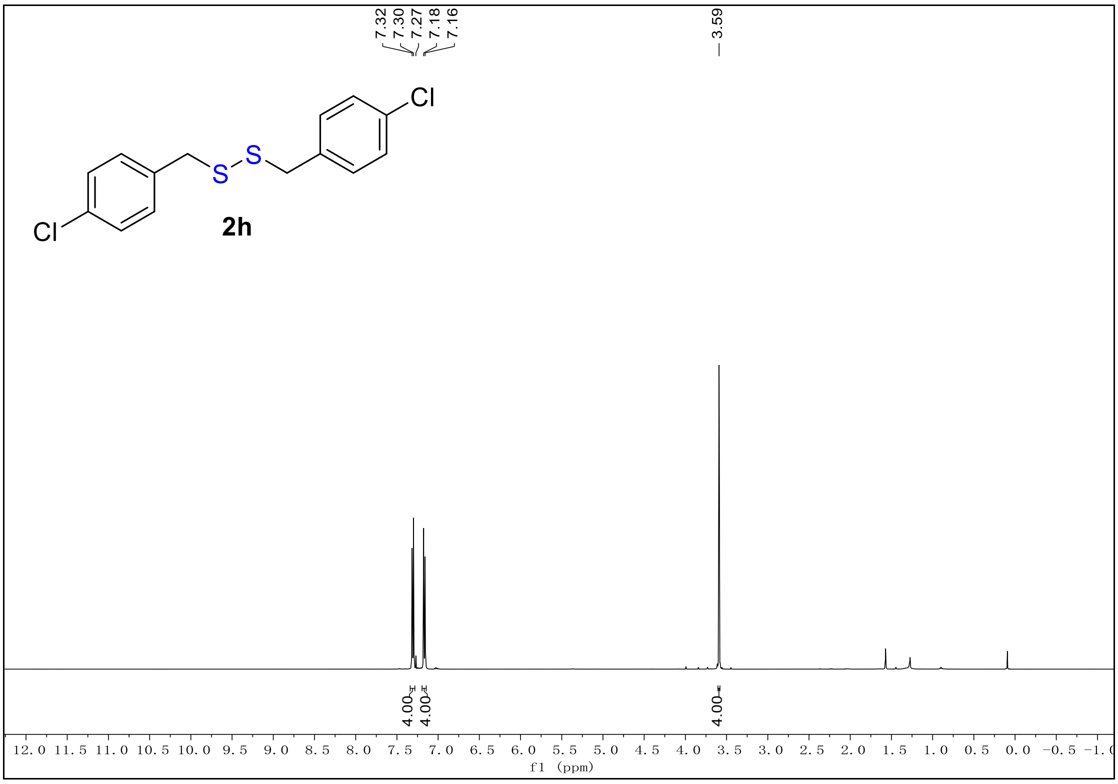
7.32
7.30
7.27
7.18
7.16
3.59
Cl
S
S
Cl
2h
4.00
4.00
4.00
12.0 11.5 11.0 10.5 10.0 9.5 9.0 8.5 8.0 7.5 7.0 6.5 6.0 5.5 5.0 4.5 4.0 3.5 3.0 2.5 2.0 1.5 1.0 0.5 0.0 -0.5
f1 (ppm)

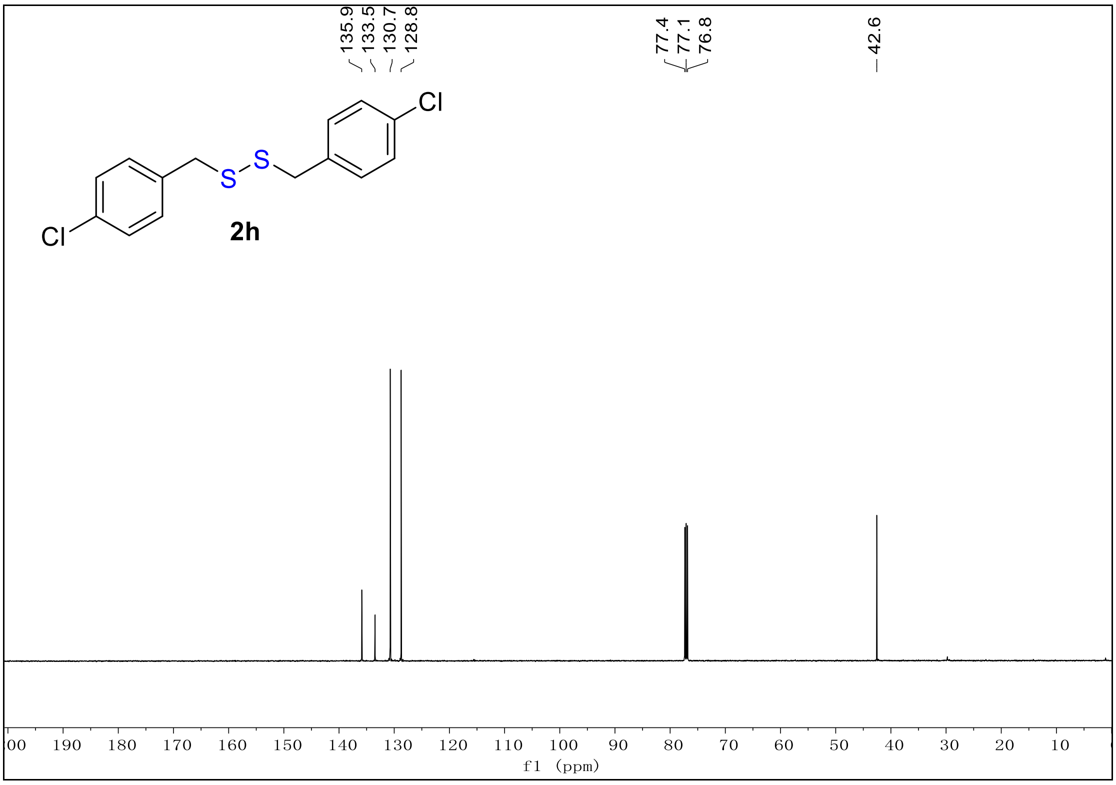
135.9
133.5
130.7
128.8
77.4
77.1
76.8
42.6
Cl
S
S
Cl
2h
190 180 170 160 150 140 130 120 110 100 90 80 70 60 50 40 30 20 10
f1 (ppm)

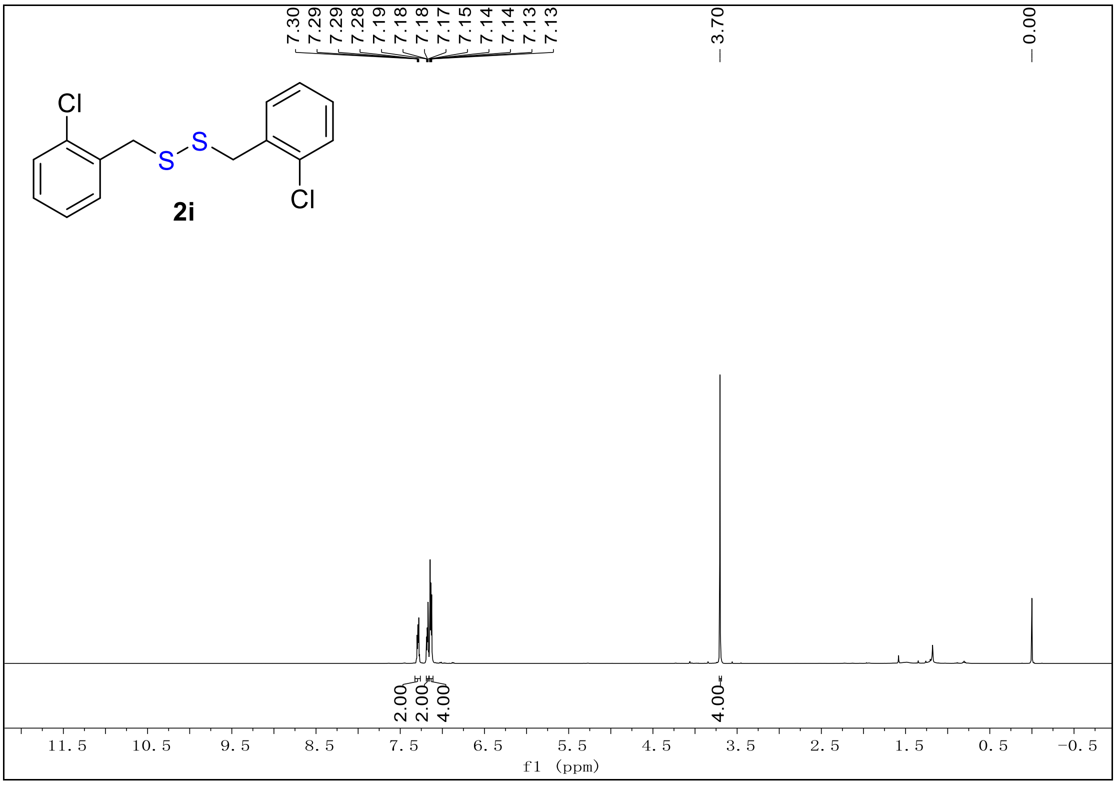

7.30
7.29
7.29
7.28
7.19
7.18
7.18
7.17
7.15
7.14
7.14
7.13
7.13
3.70
0.00
Cl
S
S
Cl
2i
2.00
2.00
4.00
4.00
11.5
10.5
9.5
8.5
7.5
6.5
5.5
4.5
3.5
2.5
1.5
0.5
-0.5
f1 (ppm)

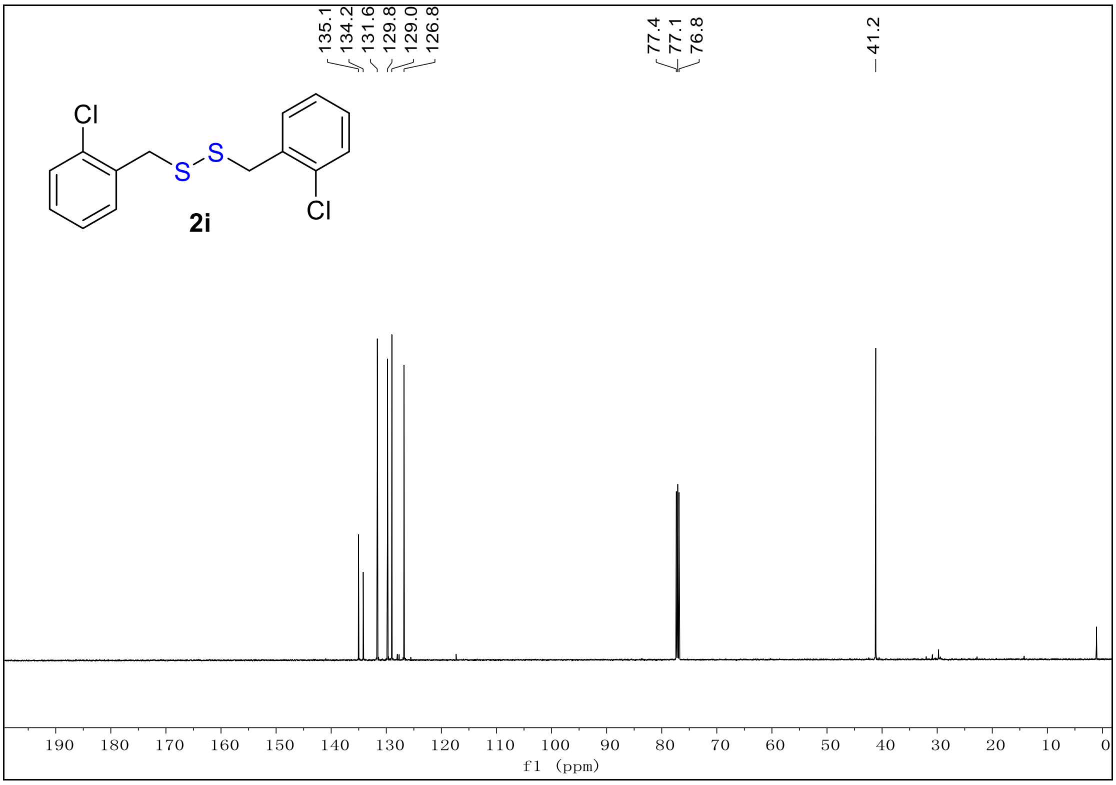

135.1
134.2
131.6
129.8
129.0
126.8
77.4
77.1
76.8
41.2
Cl
S
S
Cl
2i
190
180
170
160
150
140
130
120
110
100
90
80
70
60
50
40
30
20
10
0
f1 (ppm)

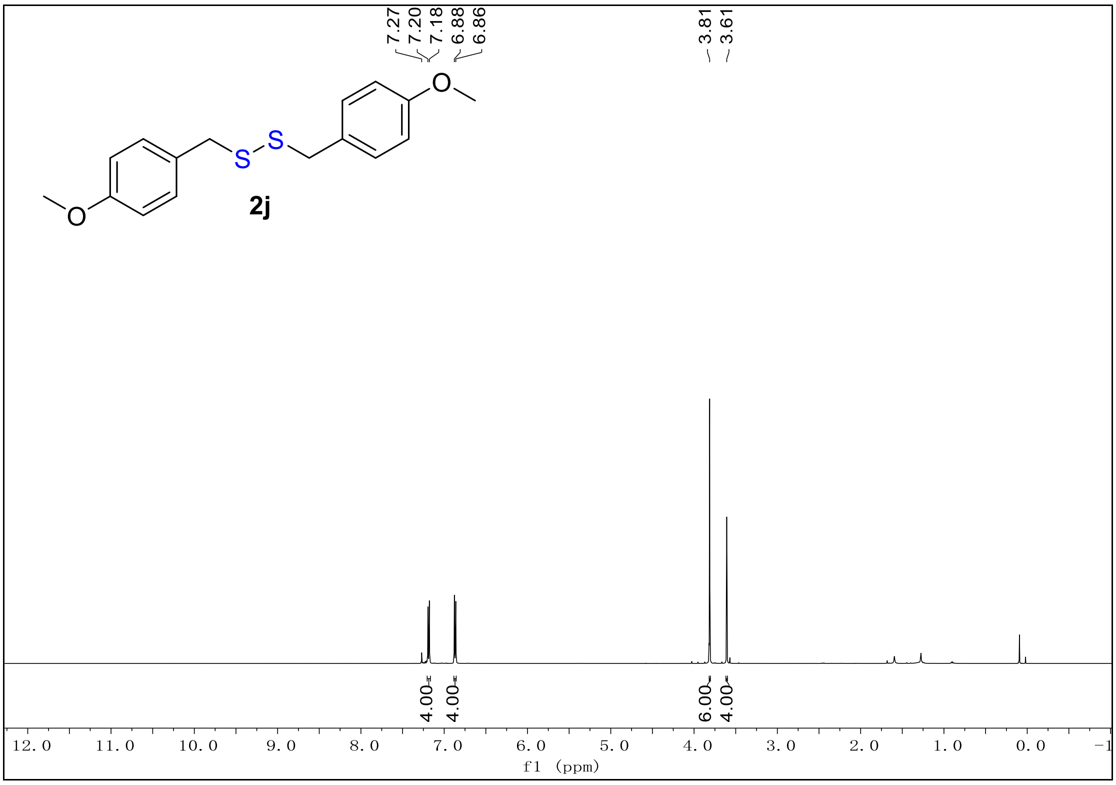
7.27
7.20
7.18
6.88
6.86
3.81
3.61
S
S
O
O
2j
4.00
4.00
6.00
4.00
12.0
11.0
10.0
9.0
8.0
7.0
6.0
5.0
4.0
3.0
2.0
1.0
0.0
f1 (ppm)

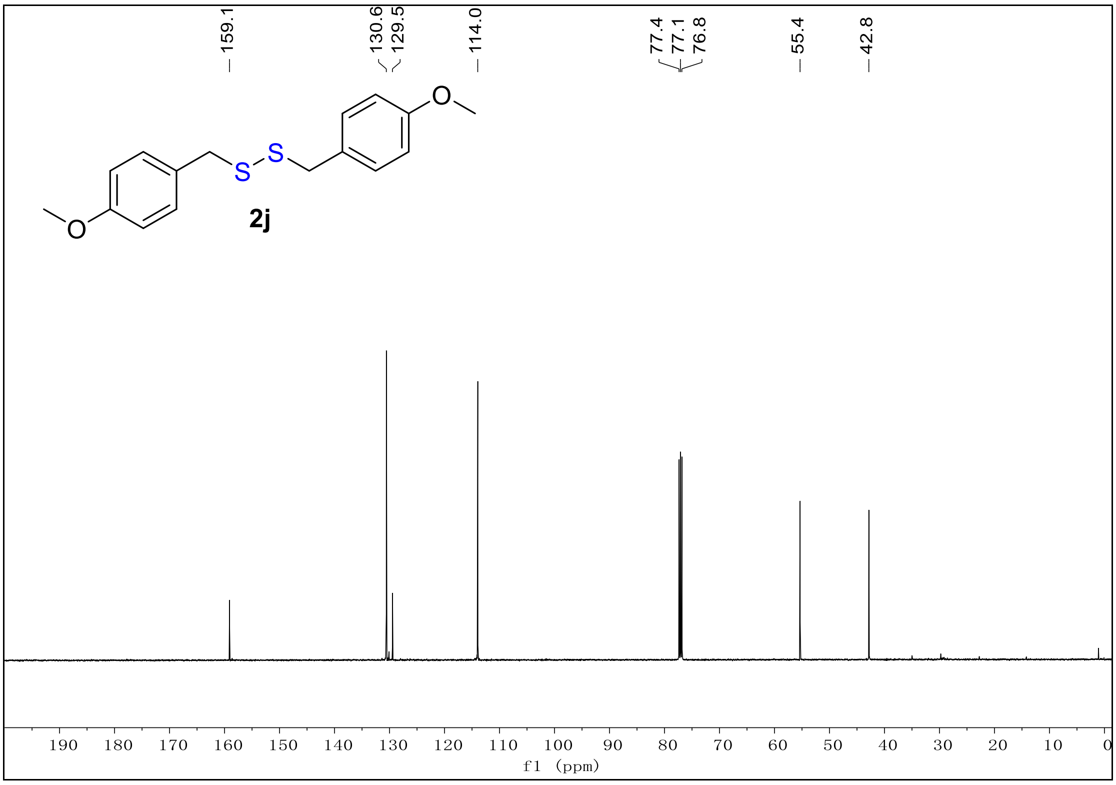
159.1
130.6
129.5
114.0
77.4
77.1
76.8
55.4
42.8
S
S
O
O
2j
190
180
170
160
150
140
130
120
110
100
90
80
70
60
50
40
30
20
10
0
f1 (ppm)

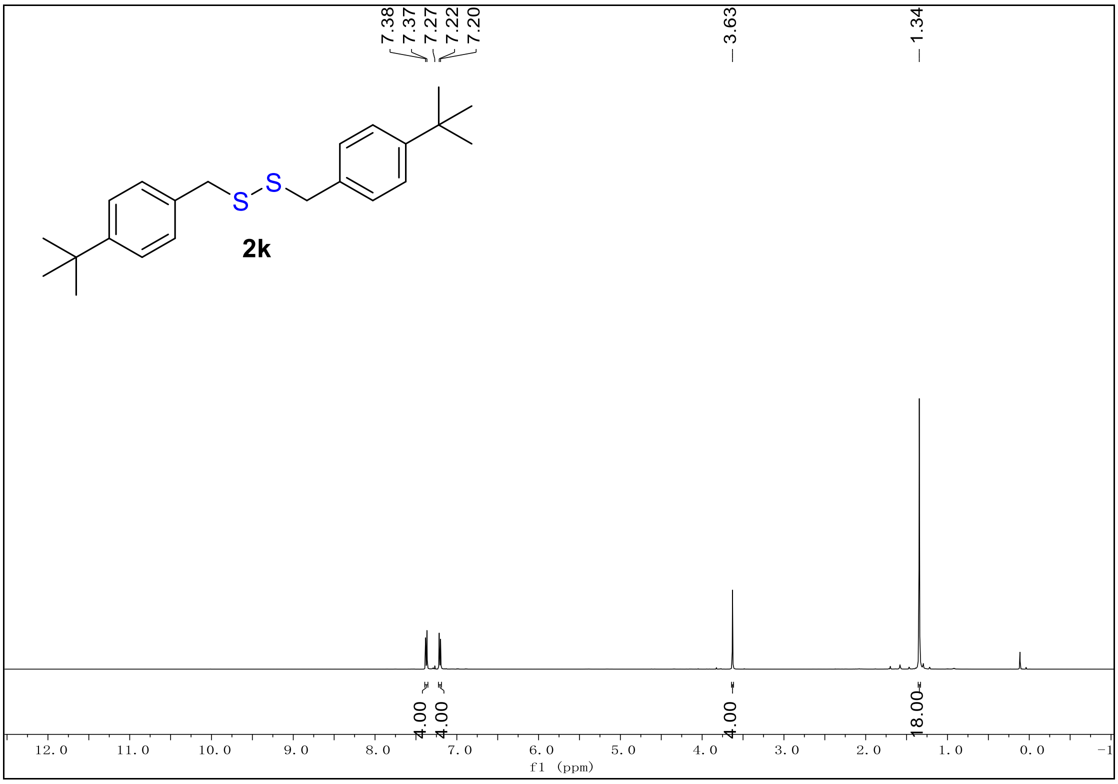
7.38
7.37
7.27
7.22
7.20
3.63
1.34
S
S
2k
4.00
4.00
4.00
18.00
12.0 11.0 10.0 9.0 8.0 7.0 6.0 5.0 4.0 3.0 2.0 1.0 0.0
f1 (ppm)

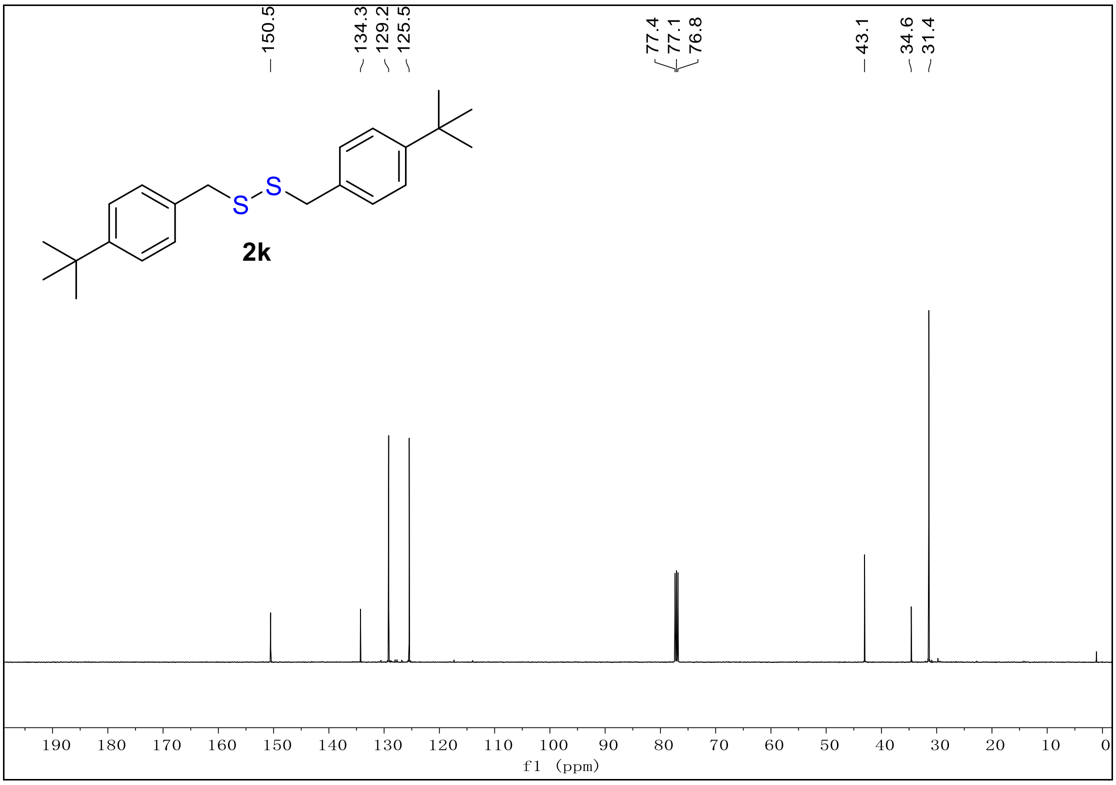
150.5
134.3
129.2
125.5
77.4
77.1
76.8
43.1
34.6
31.4
S
S
2k
190 180 170 160 150 140 130 120 110 100 90 80 70 60 50 40 30 20 10 0
f1 (ppm)

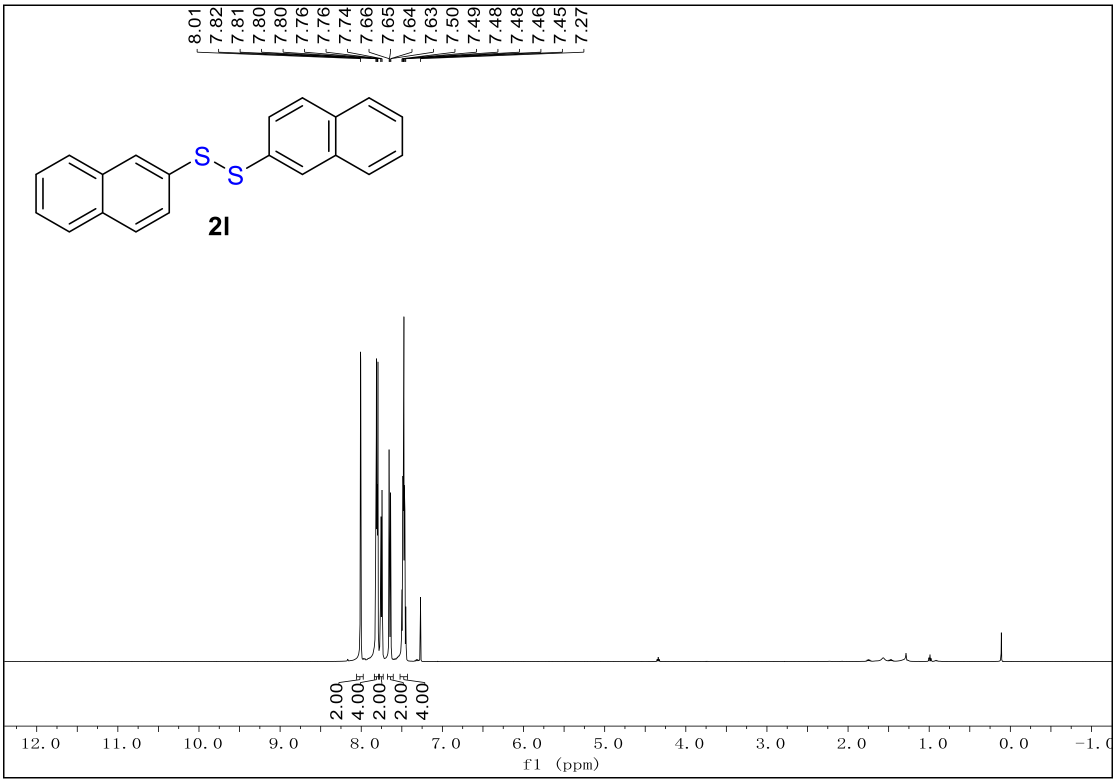
8.01
7.82
7.81
7.80
7.80
7.76
7.76
7.74
7.66
7.65
7.64
7.63
7.50
7.49
7.48
7.48
7.46
7.45
7.27
S
S
2l
2.00
4.00
2.00
2.00
4.00
12.0 11.0 10.0 9.0 8.0 7.0 6.0 5.0 4.0 3.0 2.0 1.0 0.0
f1 (ppm)

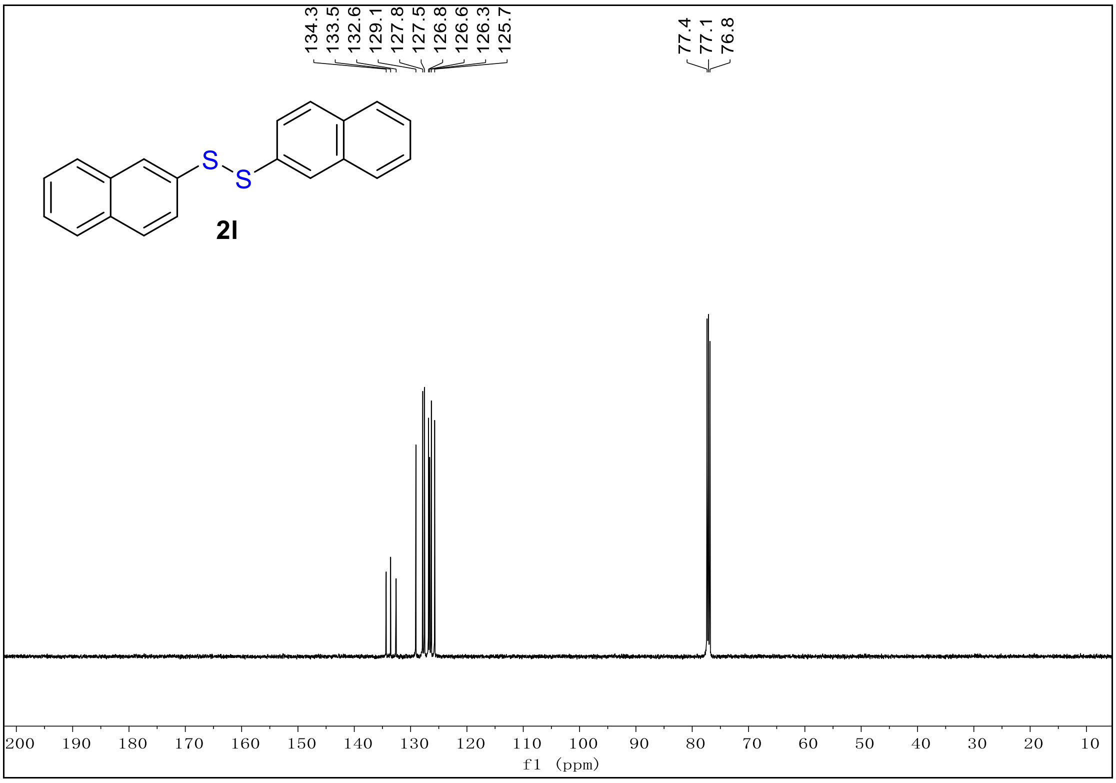
134.3
133.5
132.6
129.1
127.8
127.5
126.8
126.6
126.3
125.7
77.4
77.1
76.8
S
S
2l
200 190 180 170 160 150 140 130 120 110 100 90 80 70 60 50 40 30 20 10
f1 (ppm)

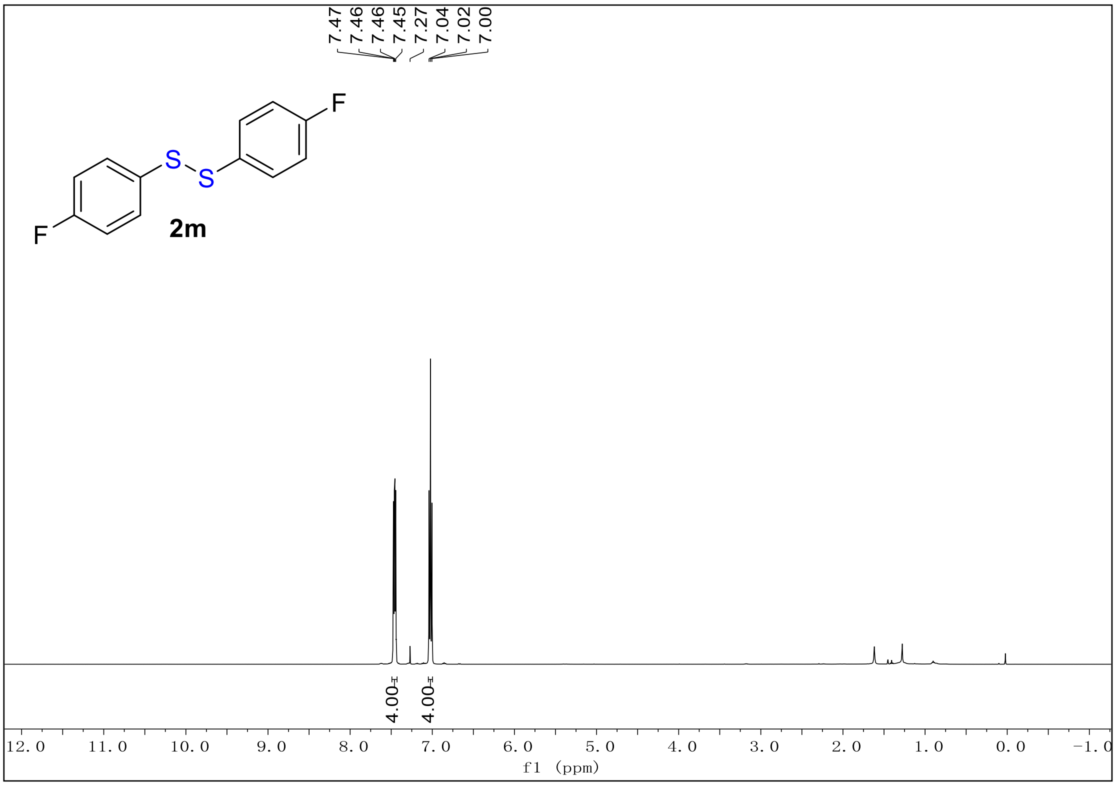

7.47
7.46
7.46
7.45
7.27
7.04
7.02
7.00
F
S
S
F
2m
4.00
4.00
12.0 11.0 10.0 9.0 8.0 7.0 6.0 5.0 4.0 3.0 2.0 1.0 0.0 -1.0
f1 (ppm)

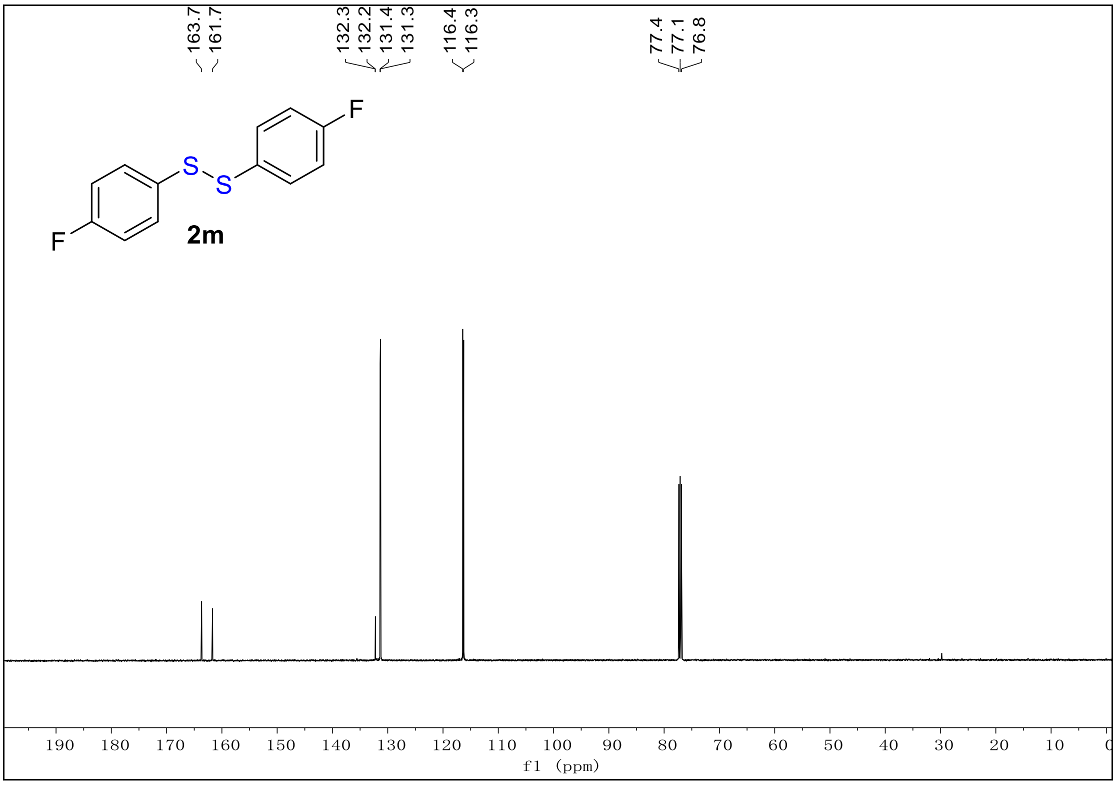

163.7
161.7
132.3
132.2
131.4
131.3
116.4
116.3
77.4
77.1
76.8
F
S
S
F
2m
190 180 170 160 150 140 130 120 110 100 90 80 70 60 50 40 30 20 10 0
f1 (ppm)

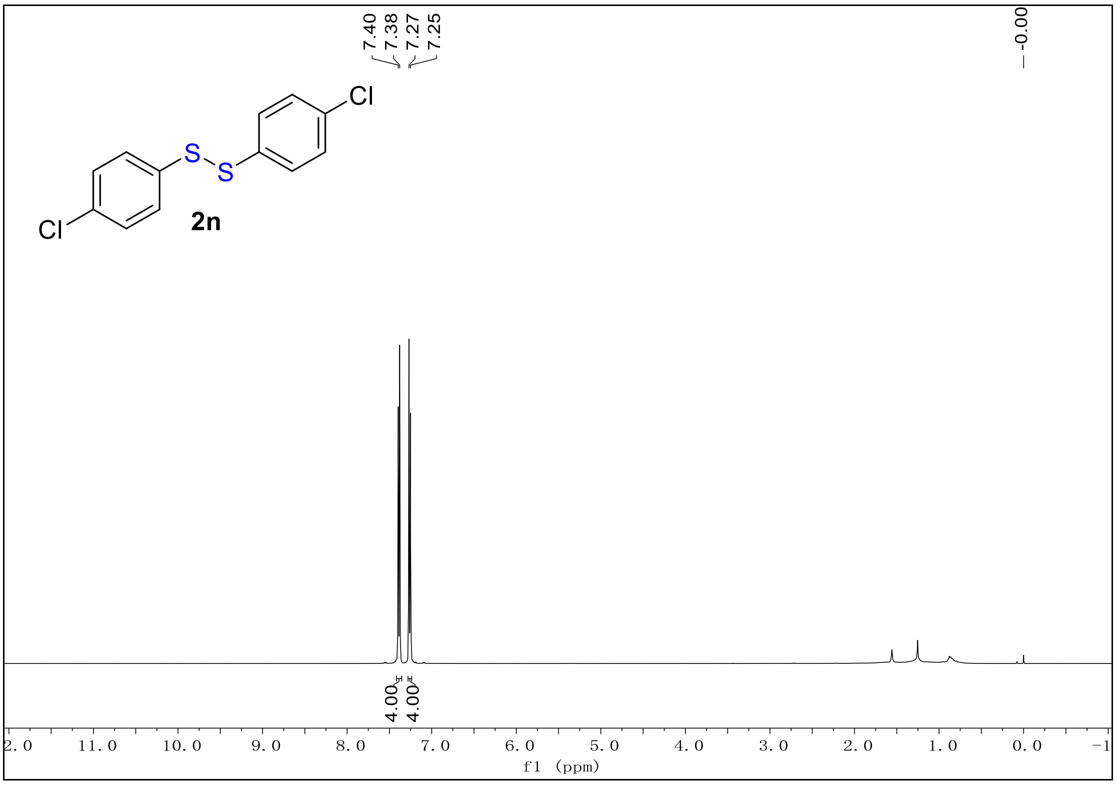
7.40
7.38
7.27
7.25
-0.00
Cl
S
S
Cl
2n
4.00
4.00
f1 (ppm)

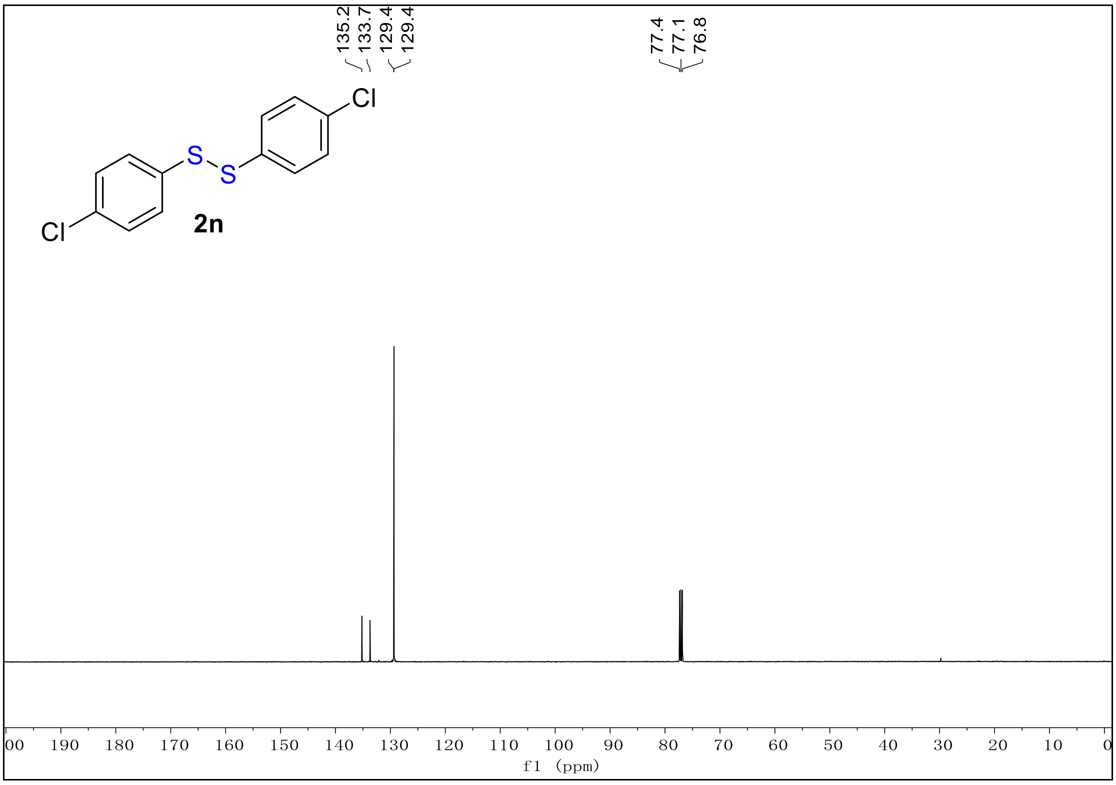
135.2
133.7
129.4
129.4
77.4
77.1
76.8
Cl
S
S
Cl
2n
f1 (ppm)

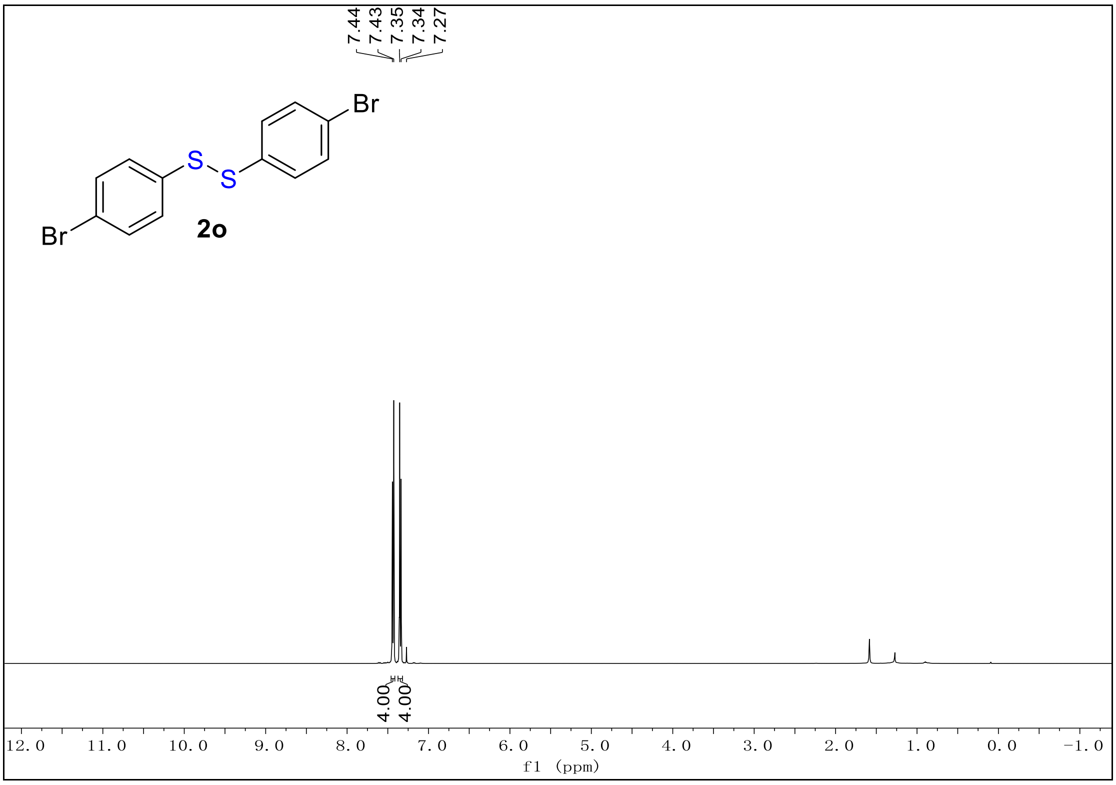
7.44
7.43
7.35
7.34
7.27
Br
S
S
Br
2o
4.00
4.00
12.0 11.0 10.0 9.0 8.0 7.0 6.0 5.0 4.0 3.0 2.0 1.0 0.0 -1.0
f1 (ppm)

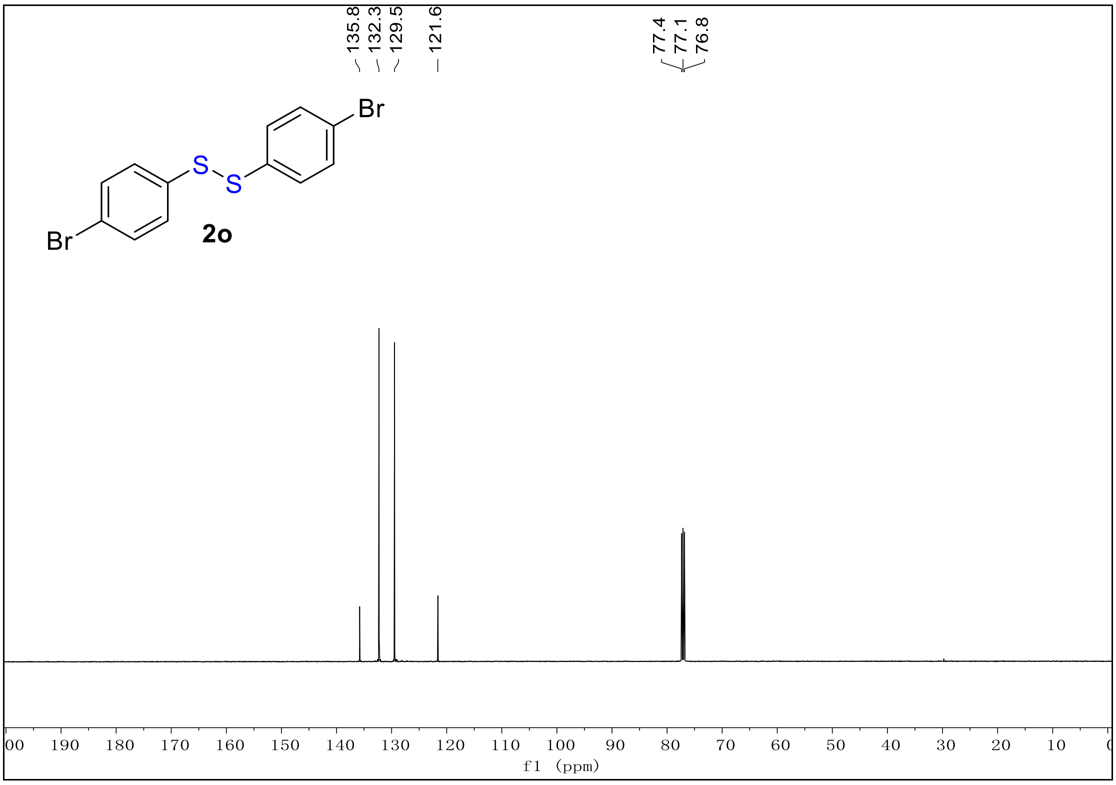
135.8
132.3
129.5
121.6
77.4
77.1
76.8
Br
S
S
Br
2o
190 180 170 160 150 140 130 120 110 100 90 80 70 60 50 40 30 20 10
f1 (ppm)

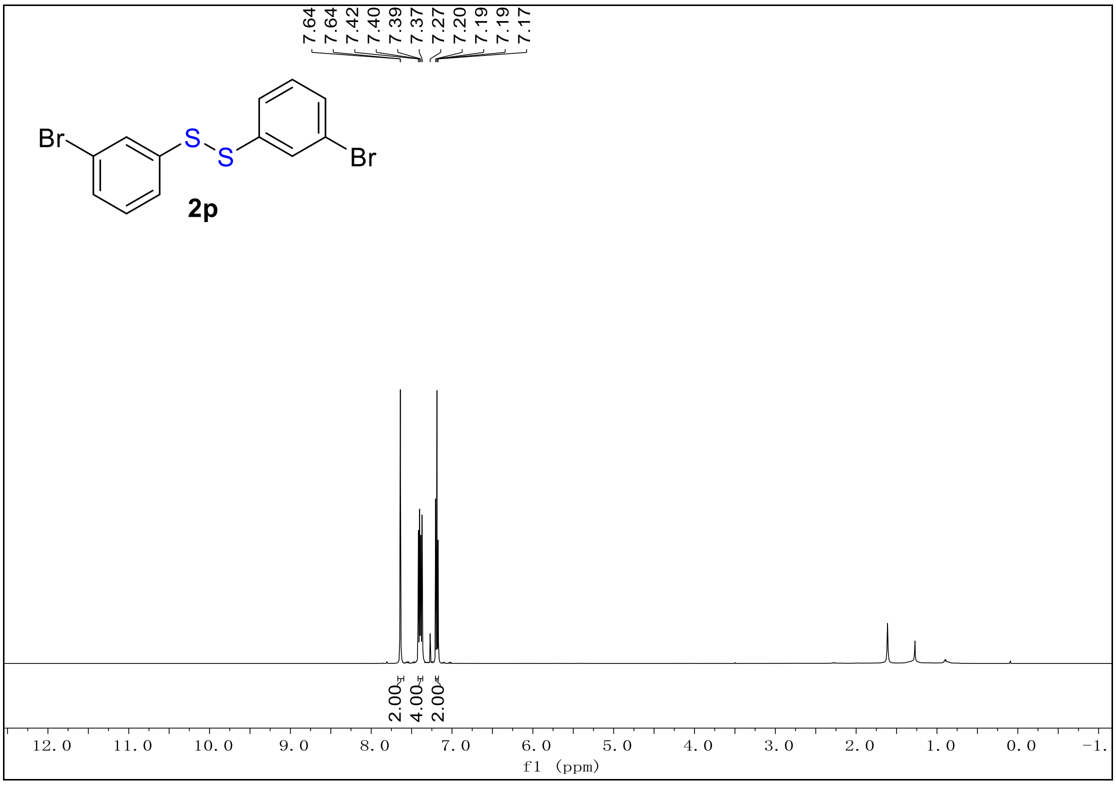
7.64
7.64
7.42
7.40
7.39
7.37
7.27
7.20
7.19
7.19
7.17
Br
S
S
Br
2p
2.00
4.00
2.00
12.0
11.0
10.0
9.0
8.0
7.0
6.0
5.0
4.0
3.0
2.0
1.0
0.0
-1.
f1 (ppm)

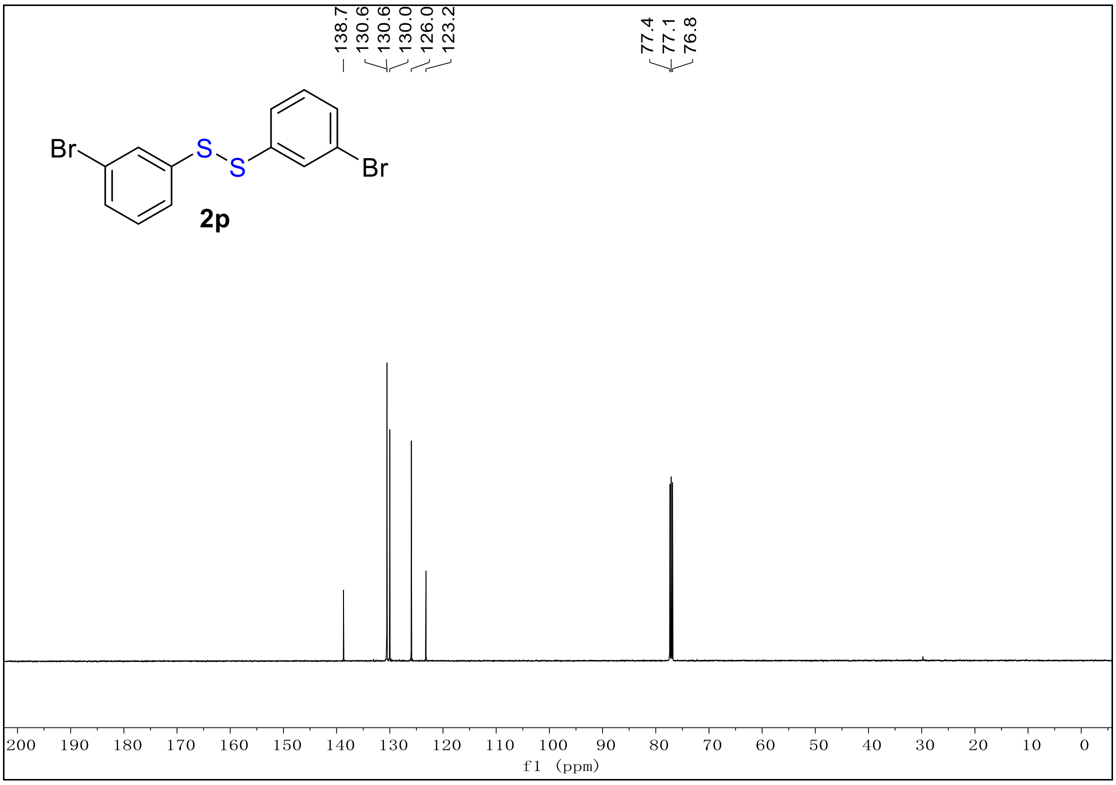
138.7
130.6
130.6
130.0
126.0
123.2
77.4
77.1
76.8
Br
S
S
Br
2p
200
190
180
170
160
150
140
130
120
110
100
90
80
70
60
50
40
30
20
10
0
f1 (ppm)

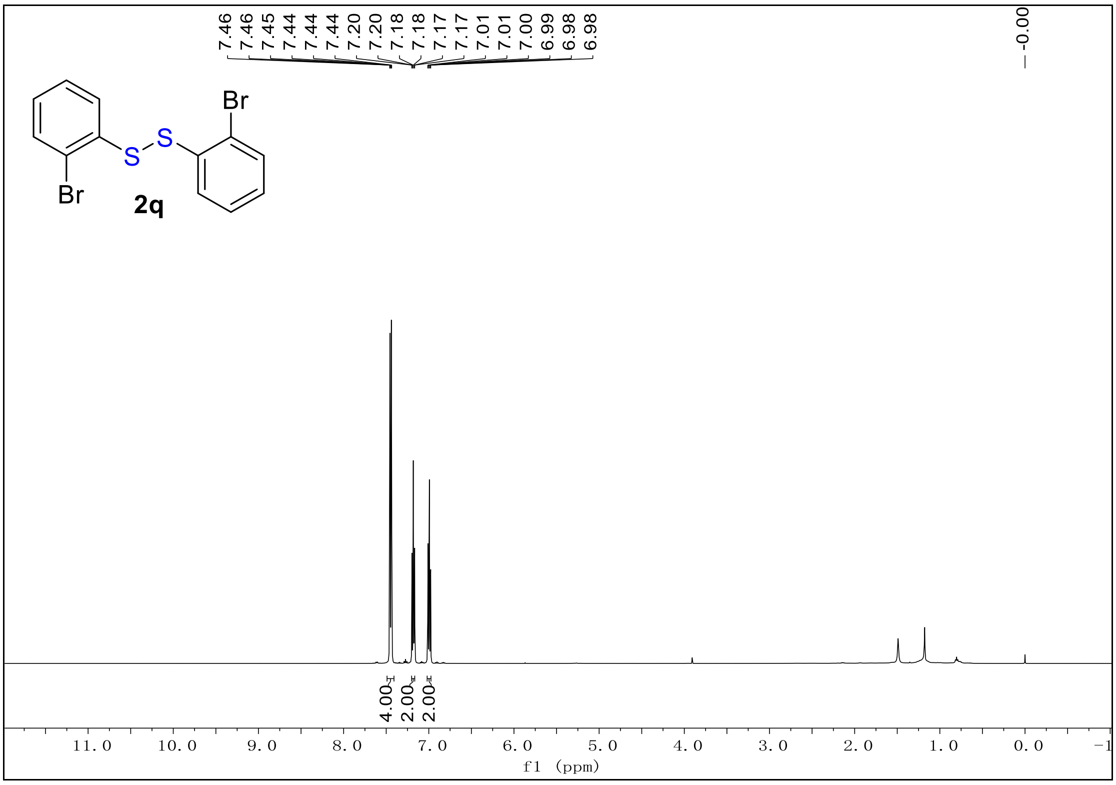
7.46
7.46
7.45
7.44
7.44
7.44
7.20
7.20
7.18
7.18
7.17
7.17
7.01
7.01
7.00
6.99
6.98
6.98
-0.00
Br
S
S
Br
2q
4.00
2.00
2.00
11.0
10.0
9.0
8.0
7.0
6.0
5.0
4.0
3.0
2.0
1.0
0.0
f1 (ppm)

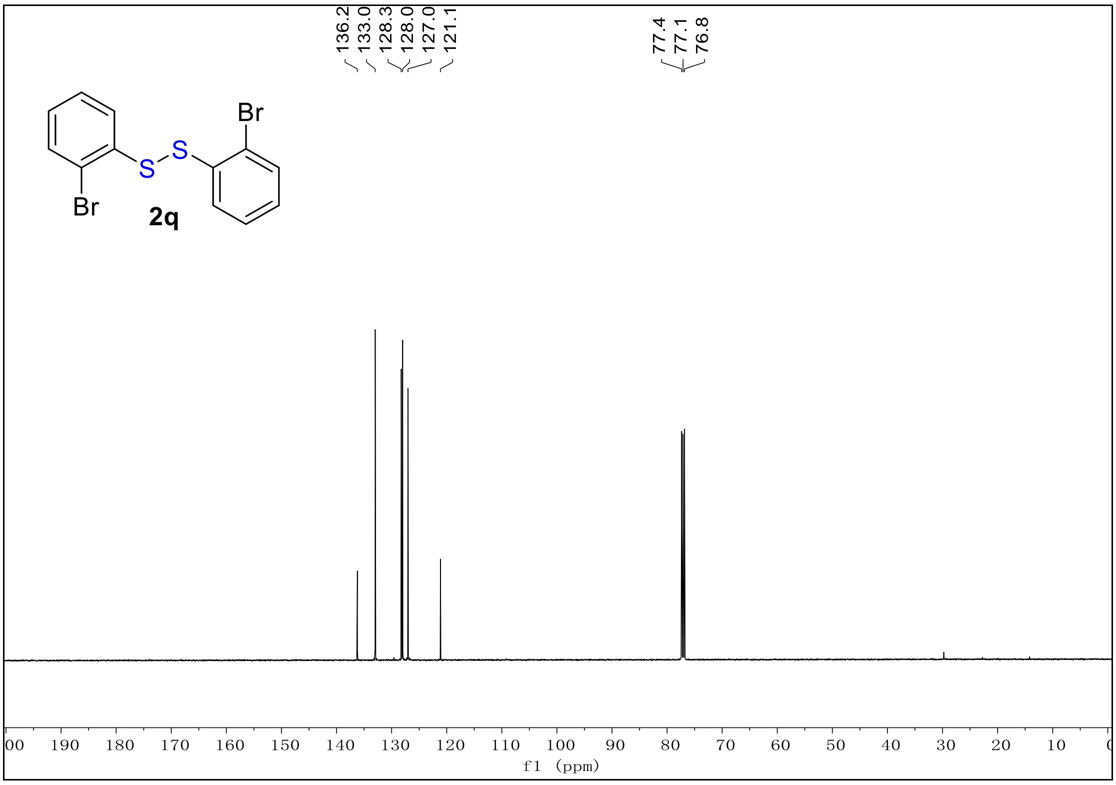
136.2
133.0
128.3
128.0
127.0
121.1
77.4
77.1
76.8
Br
S
S
Br
2q
190
180
170
160
150
140
130
120
110
100
90
80
70
60
50
40
30
20
10
f1 (ppm)

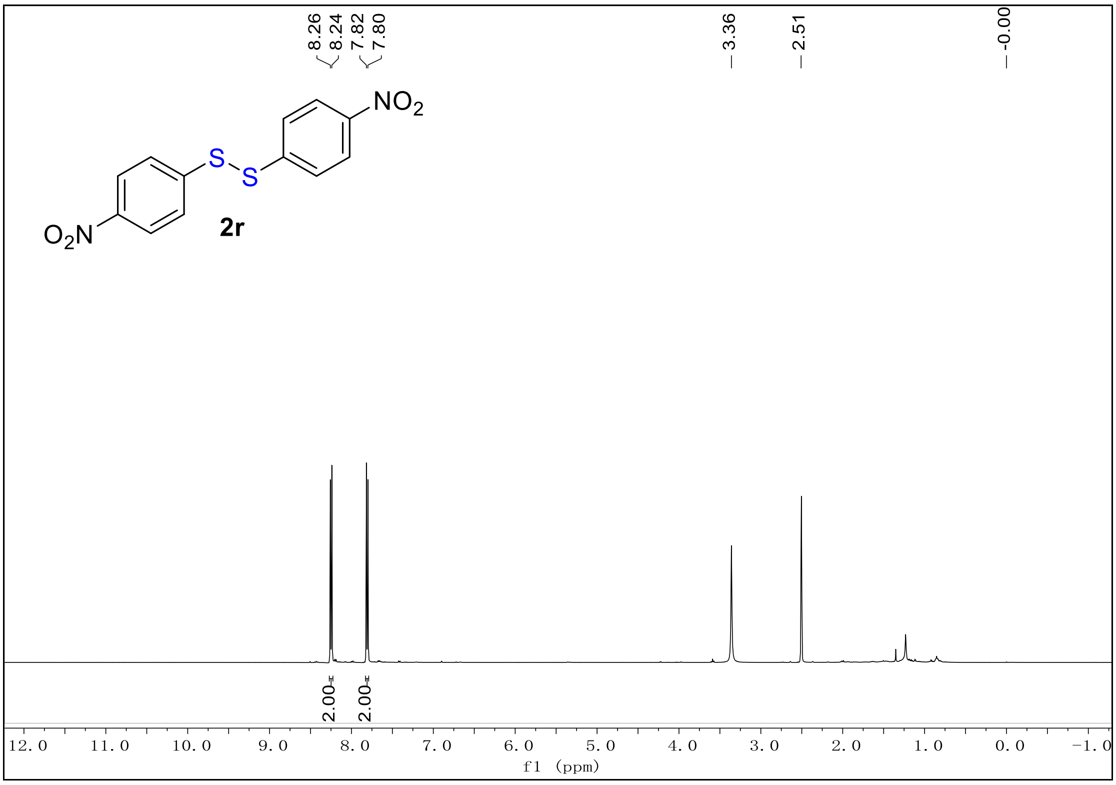
8.26
8.24
7.82
7.80
3.36
2.51
-0.00
NO2
O2N
S
S
2r
2.00
2.00
12.0
11.0
10.0
9.0
8.0
7.0
6.0
5.0
4.0
3.0
2.0
1.0
0.0
-1.0
f1 (ppm)

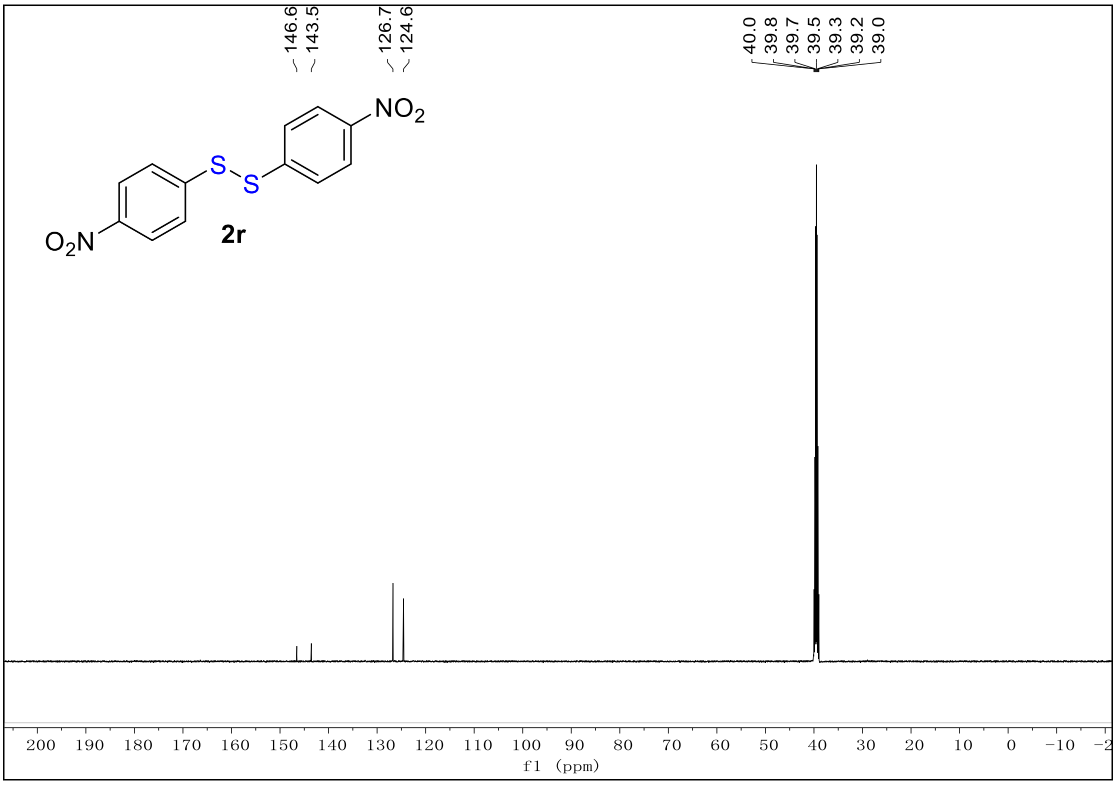
146.6
143.5
126.7
124.6
40.0
39.8
39.7
39.5
39.3
39.2
39.0
NO2
O2N
S
S
2r
200
190
180
170
160
150
140
130
120
110
100
90
80
70
60
50
40
30
20
10
0
-10
f1 (ppm)

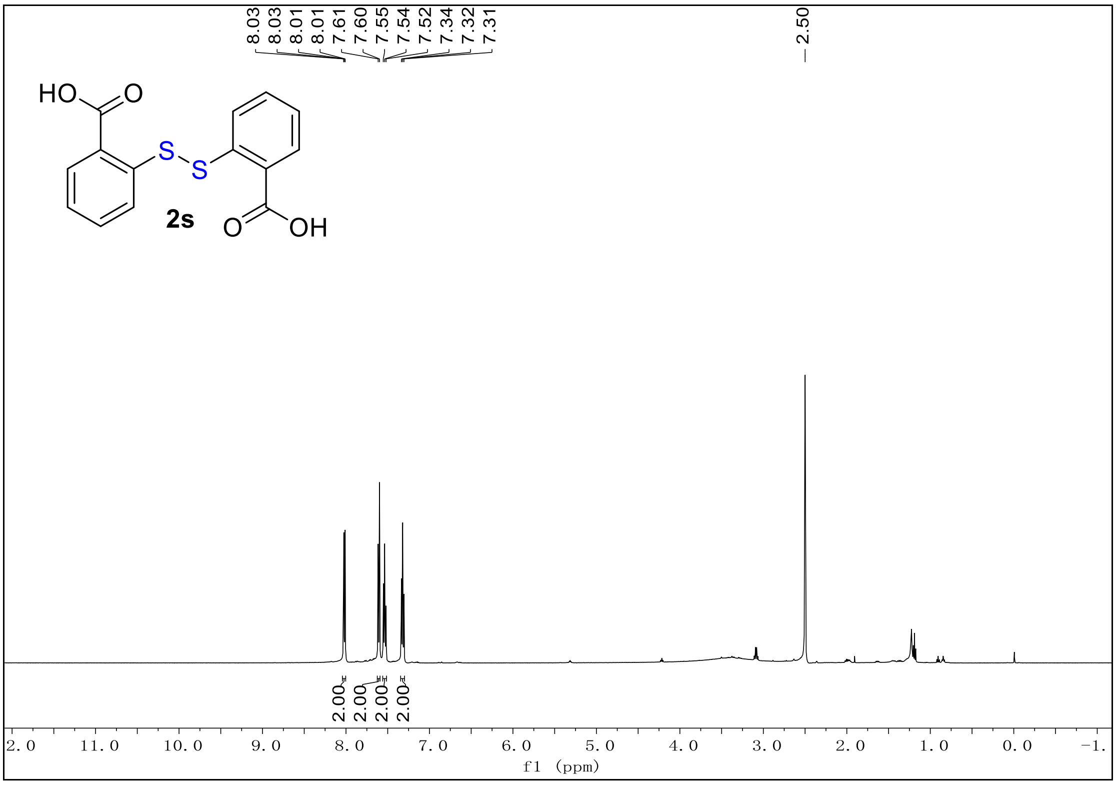
8.03
8.03
8.01
8.01
7.61
7.60
7.55
7.54
7.52
7.34
7.32
7.31
2.50
HO
O
S
S
2s
O
OH
2.00
2.00
2.00
2.00
f1 (ppm)

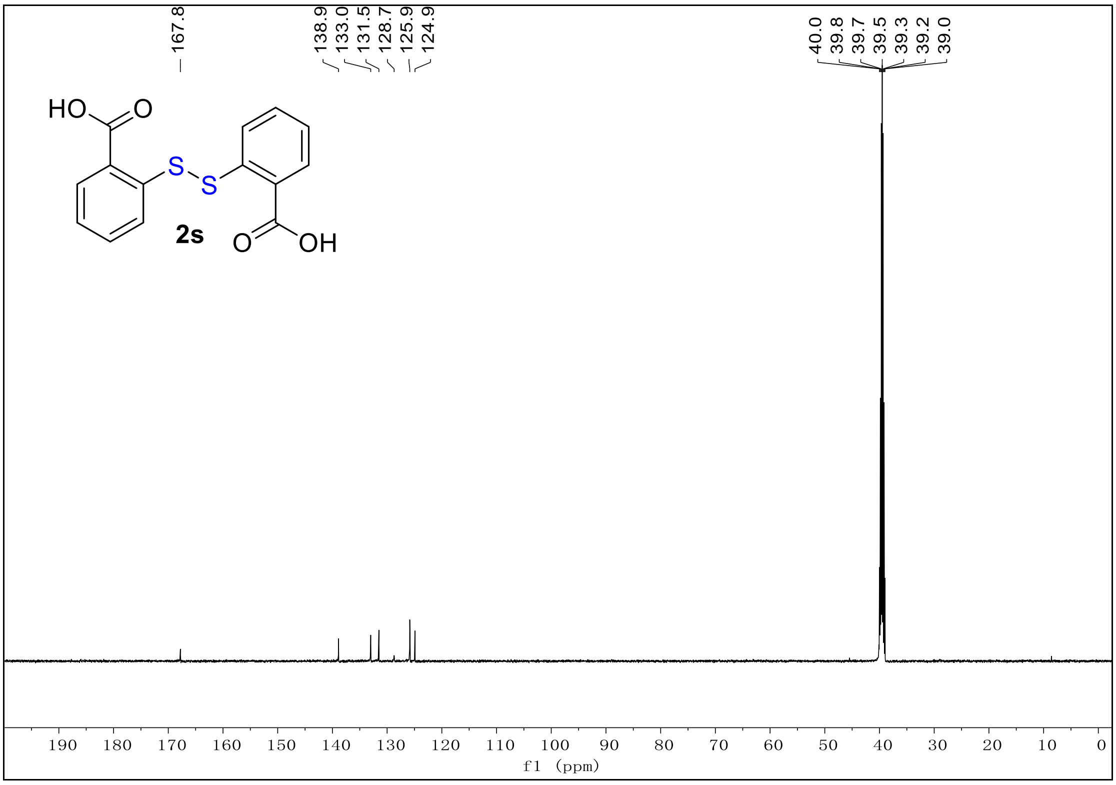
167.8
138.9
133.0
131.5
128.7
125.9
124.9
40.0
39.8
39.7
39.5
39.3
39.2
39.0
HO
O
S
S
2s
O
OH
f1 (ppm)

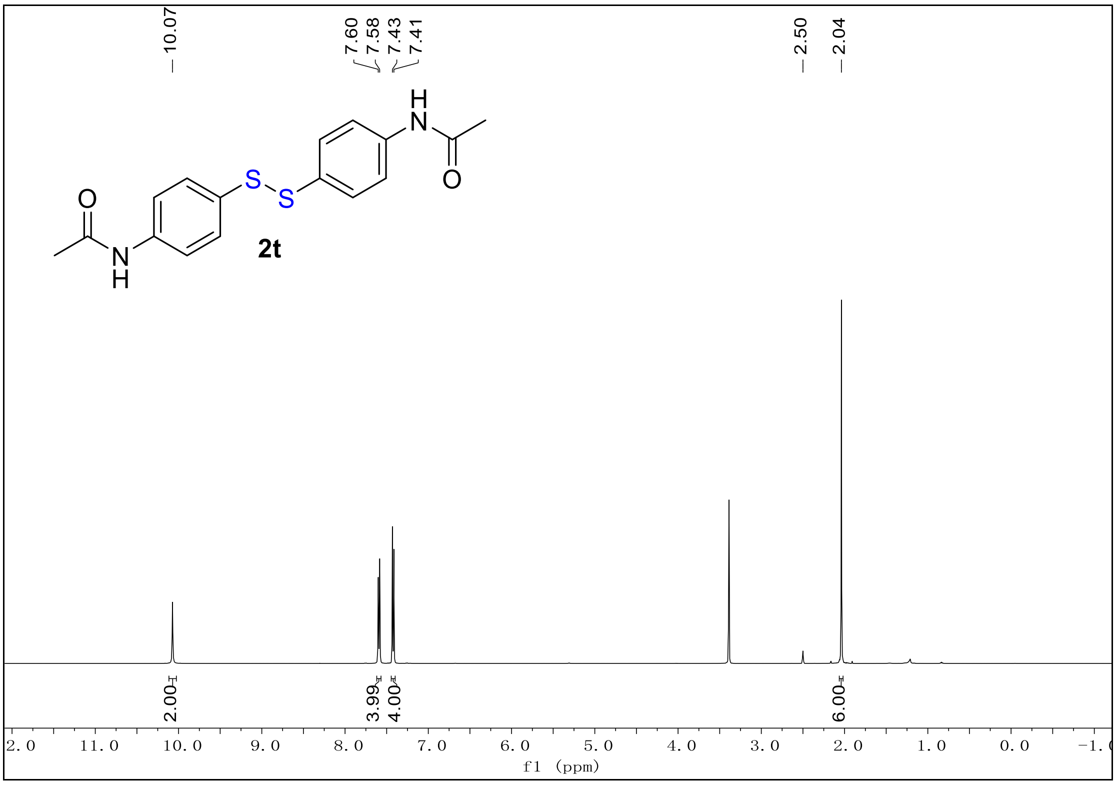
10.07
7.60
7.58
7.43
7.41
2.50
2.04
2t
2.00
3.99
4.00
6.00
f1 (ppm)

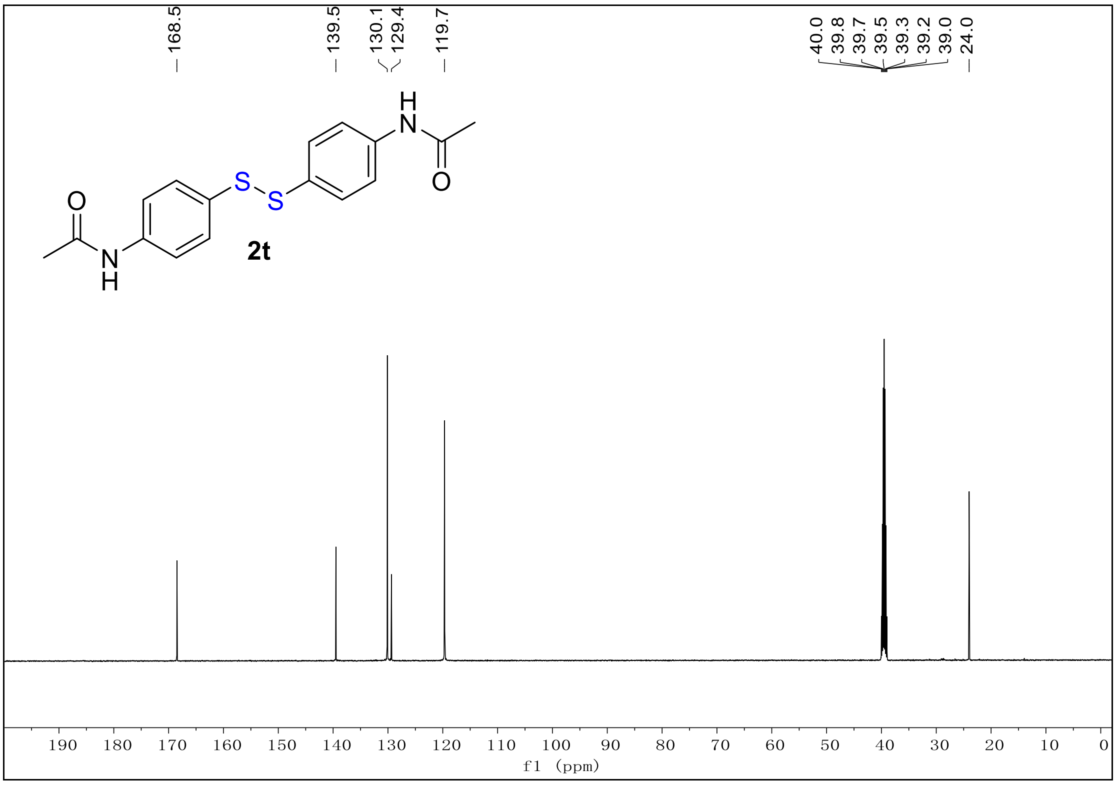
168.5
139.5
130.1
129.4
119.7
40.0
39.8
39.7
39.5
39.3
39.2
39.0
24.0
2t
f1 (ppm)

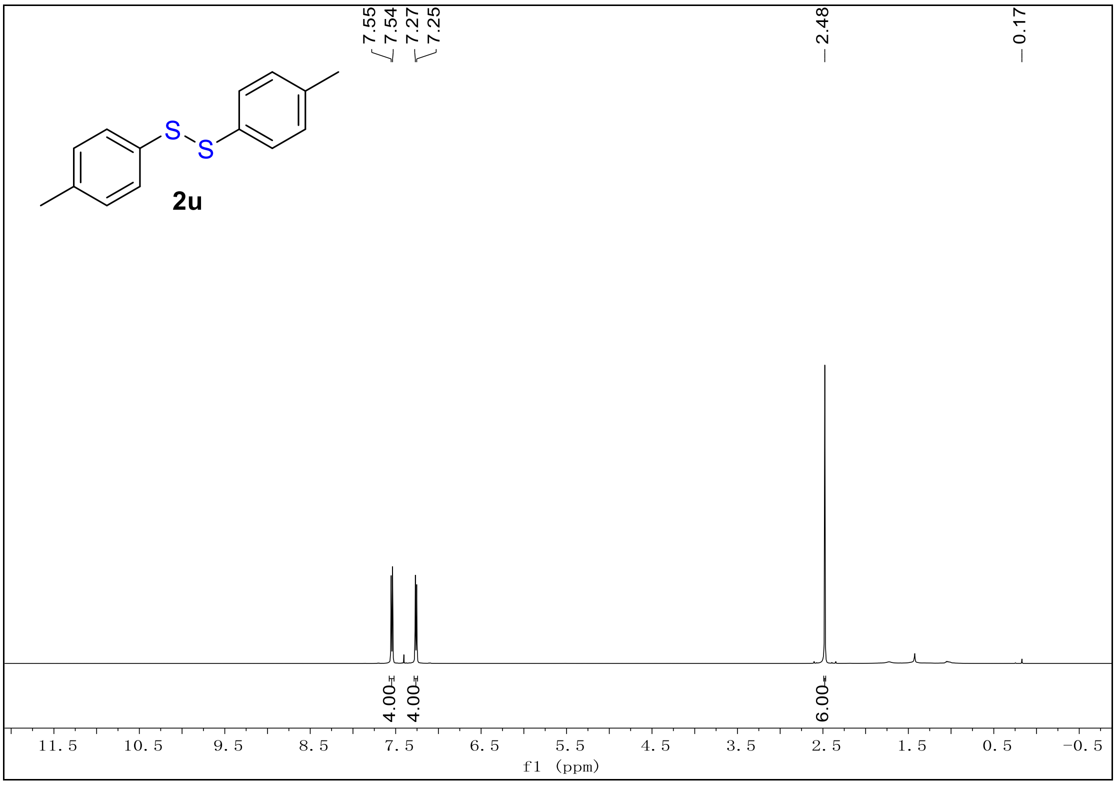
7.55
7.54
7.27
7.25
2.48
0.17
S
S
2u
4.00
4.00
6.00
11.5 10.5 9.5 8.5 7.5 6.5 5.5 4.5 3.5 2.5 1.5 0.5 -0.5
f1 (ppm)

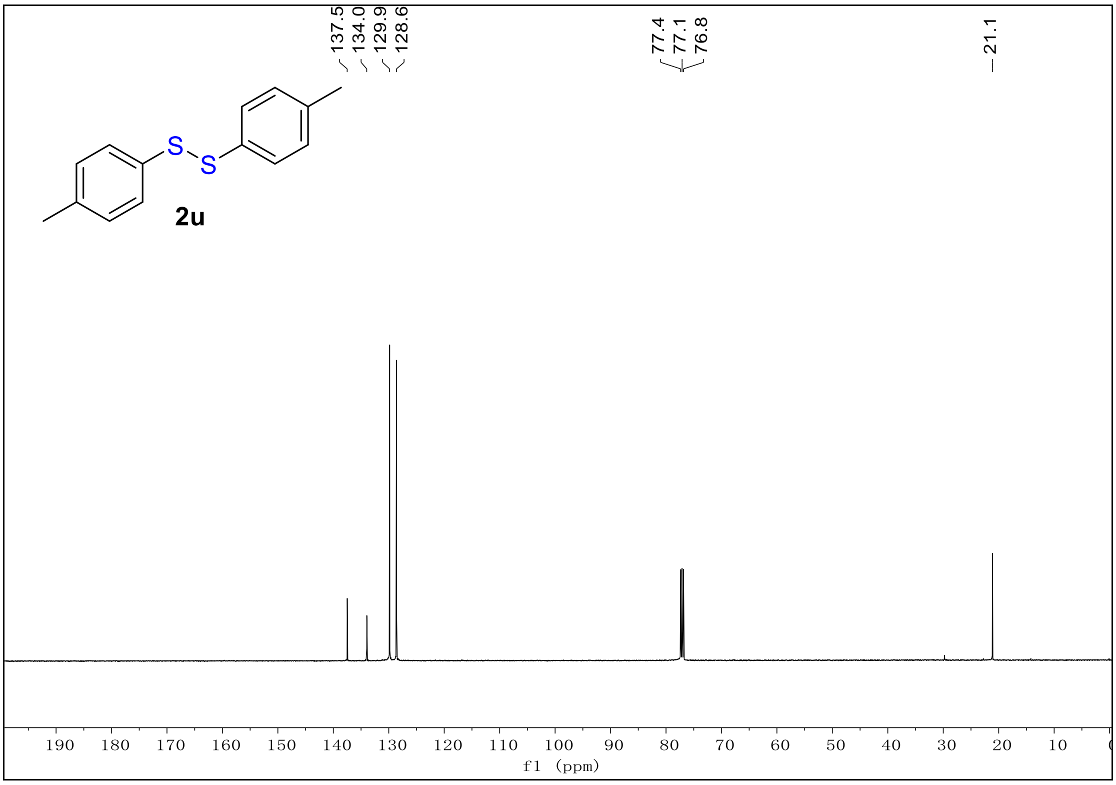
137.5
134.0
129.9
128.6
77.4
77.1
76.8
21.1
S
S
2u
190 180 170 160 150 140 130 120 110 100 90 80 70 60 50 40 30 20 10
f1 (ppm)

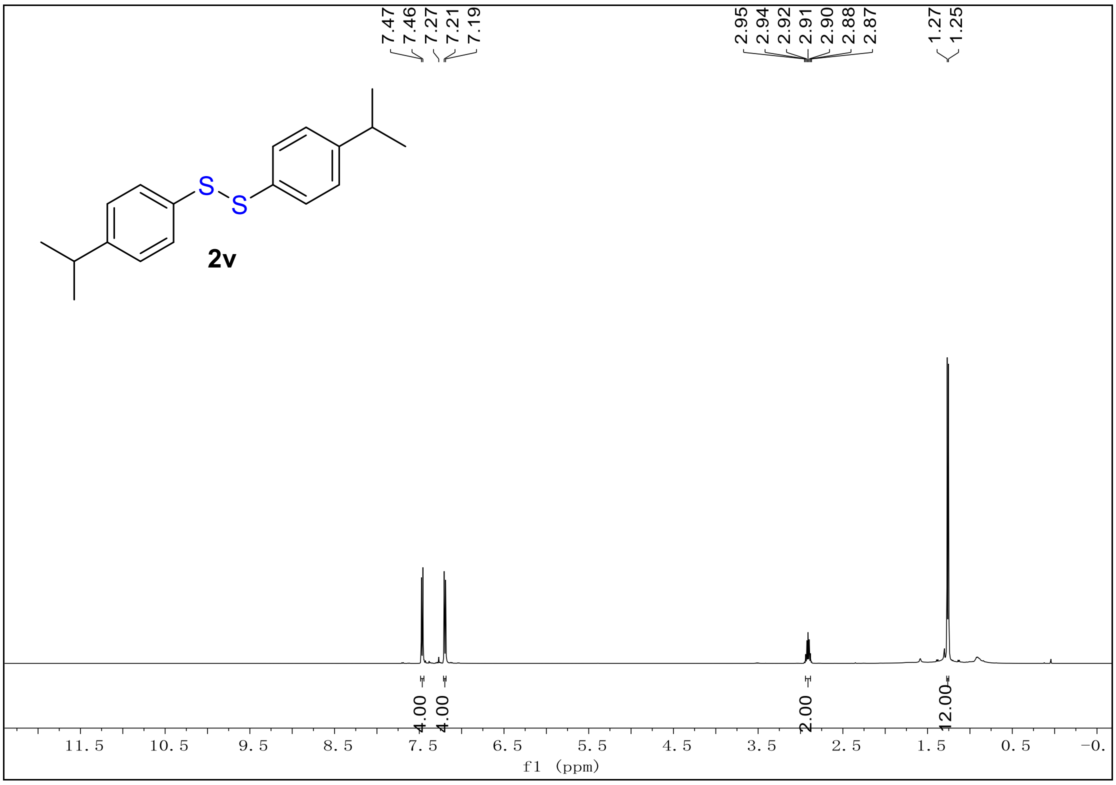
7.47
7.46
7.27
7.21
7.19
2.95
2.94
2.92
2.91
2.90
2.88
2.87
1.27
1.25
S
S
2v
4.00
4.00
2.00
12.00
11.5 10.5 9.5 8.5 7.5 6.5 5.5 4.5 3.5 2.5 1.5 0.5 -0.
f1 (ppm)

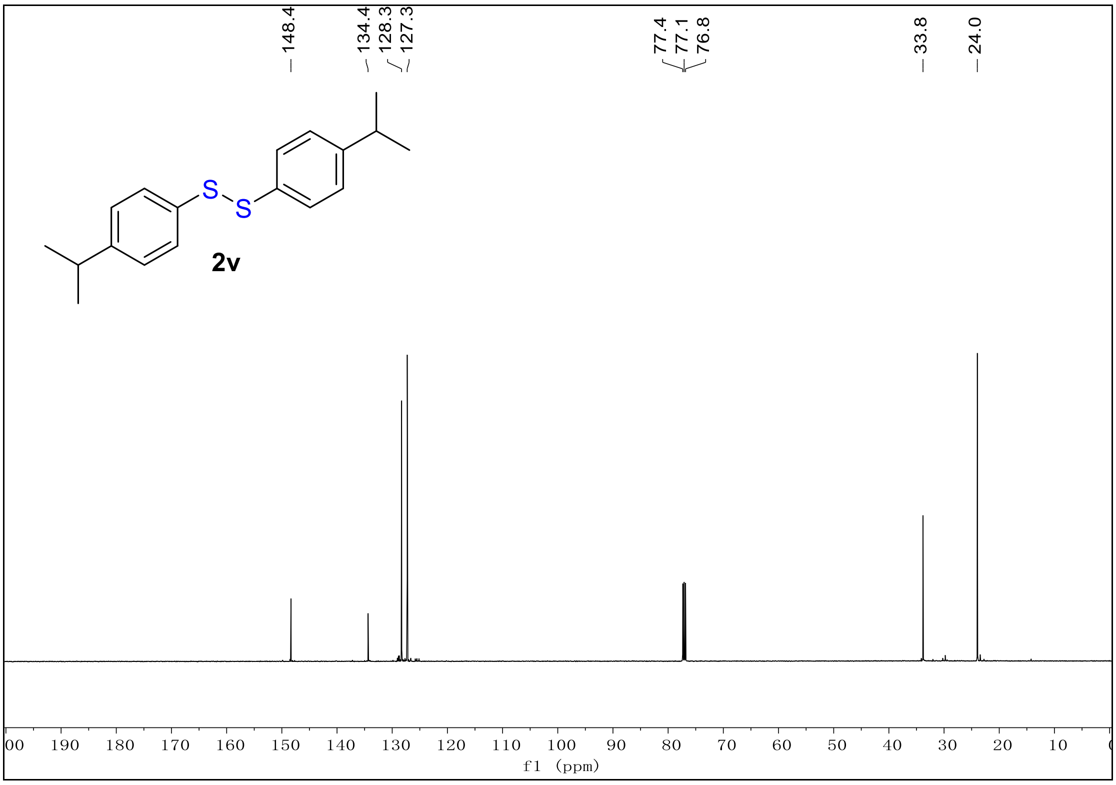
148.4
134.4
128.3
127.3
77.4
77.1
76.8
33.8
24.0
S
S
2v
00 190 180 170 160 150 140 130 120 110 100 90 80 70 60 50 40 30 20 10
f1 (ppm)

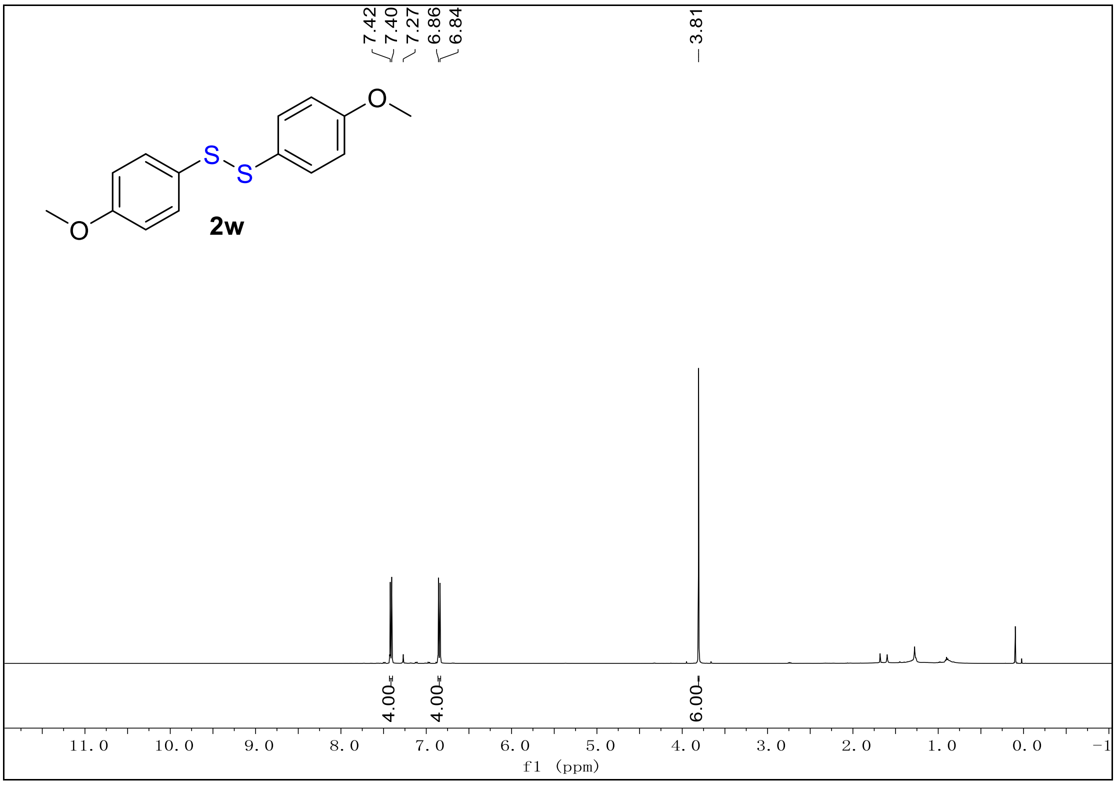
7.42
7.40
7.27
6.86
6.84
3.81
2w
4.00
4.00
6.00
11.0 10.0 9.0 8.0 7.0 6.0 5.0 4.0 3.0 2.0 1.0 0.0
f1 (ppm)

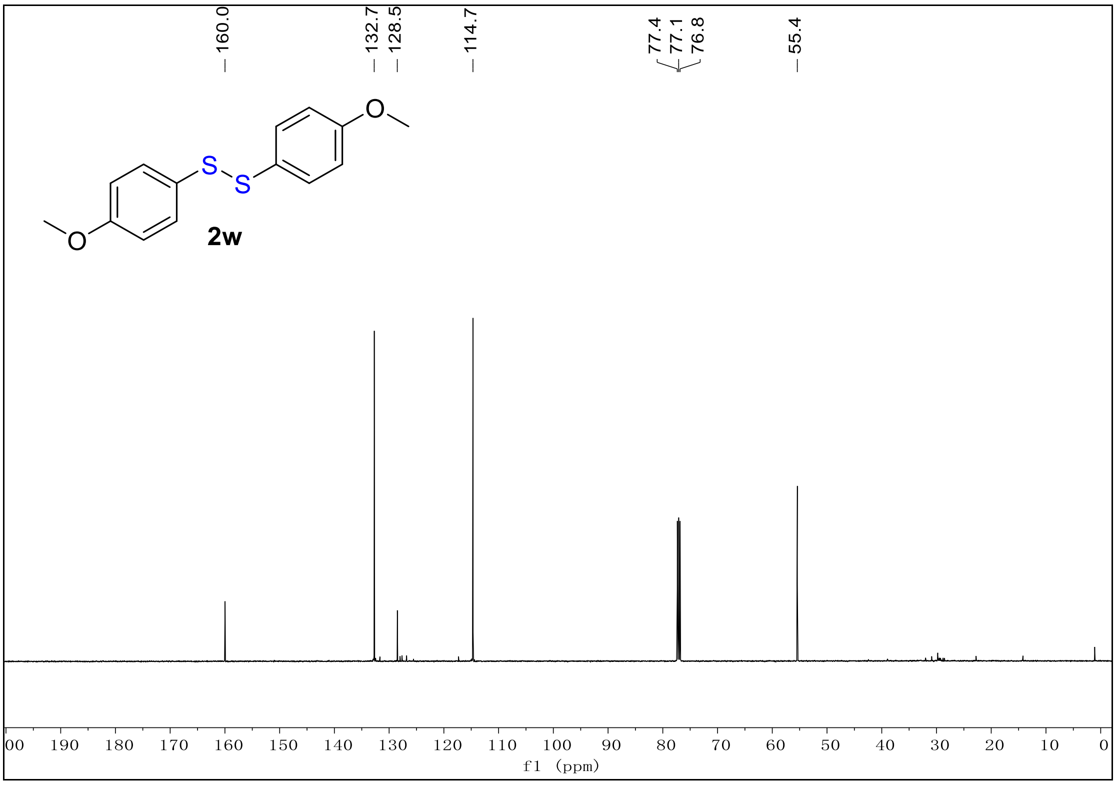
160.0
132.7
128.5
114.7
77.4
77.1
76.8
55.4
2w
190 180 170 160 150 140 130 120 110 100 90 80 70 60 50 40 30 20 10 0
f1 (ppm)

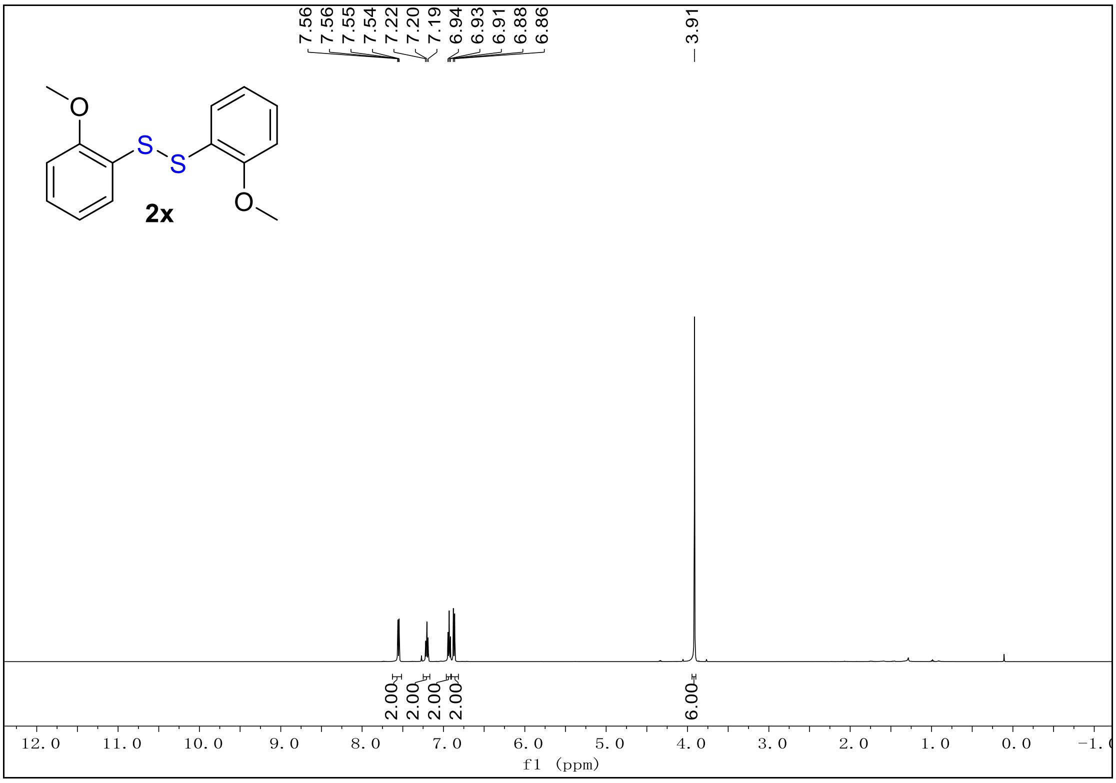
7.56
7.56
7.55
7.54
7.22
7.20
7.19
6.94
6.93
6.91
6.88
6.86
3.91
2x
2.00
2.00
2.00
2.00
6.00
f1 (ppm)

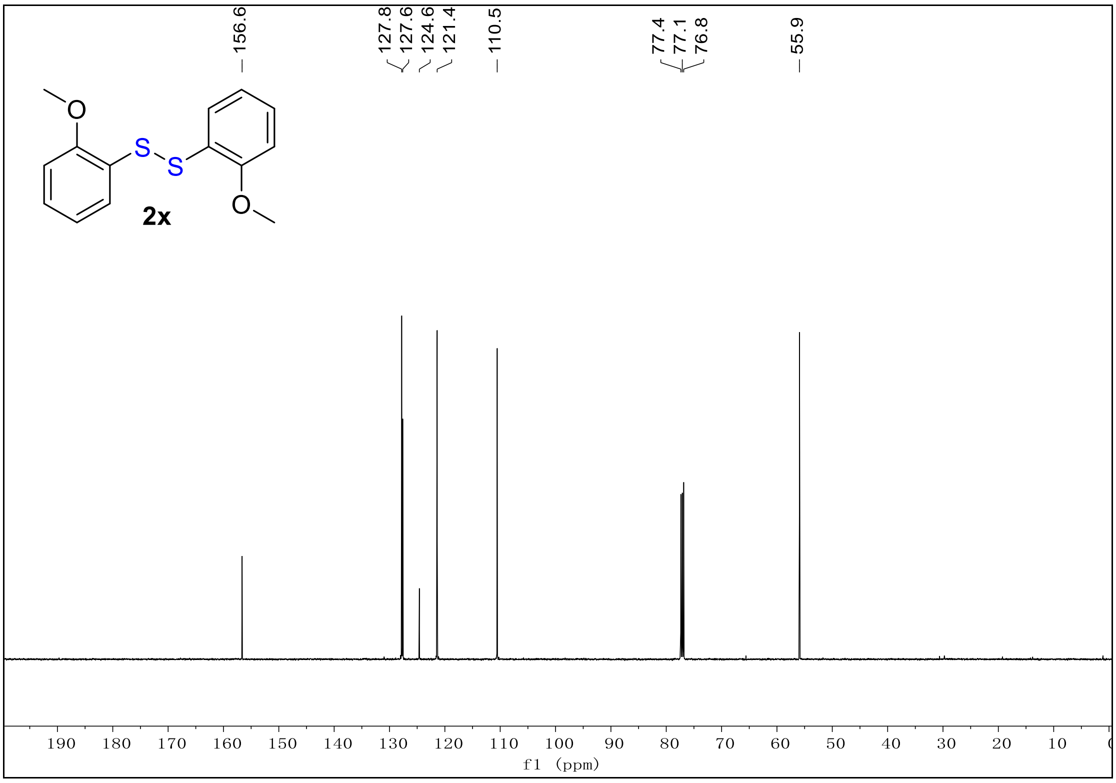
156.6
127.8
127.6
124.6
121.4
110.5
77.4
77.1
76.8
55.9
2x
f1 (ppm)

^{13}C

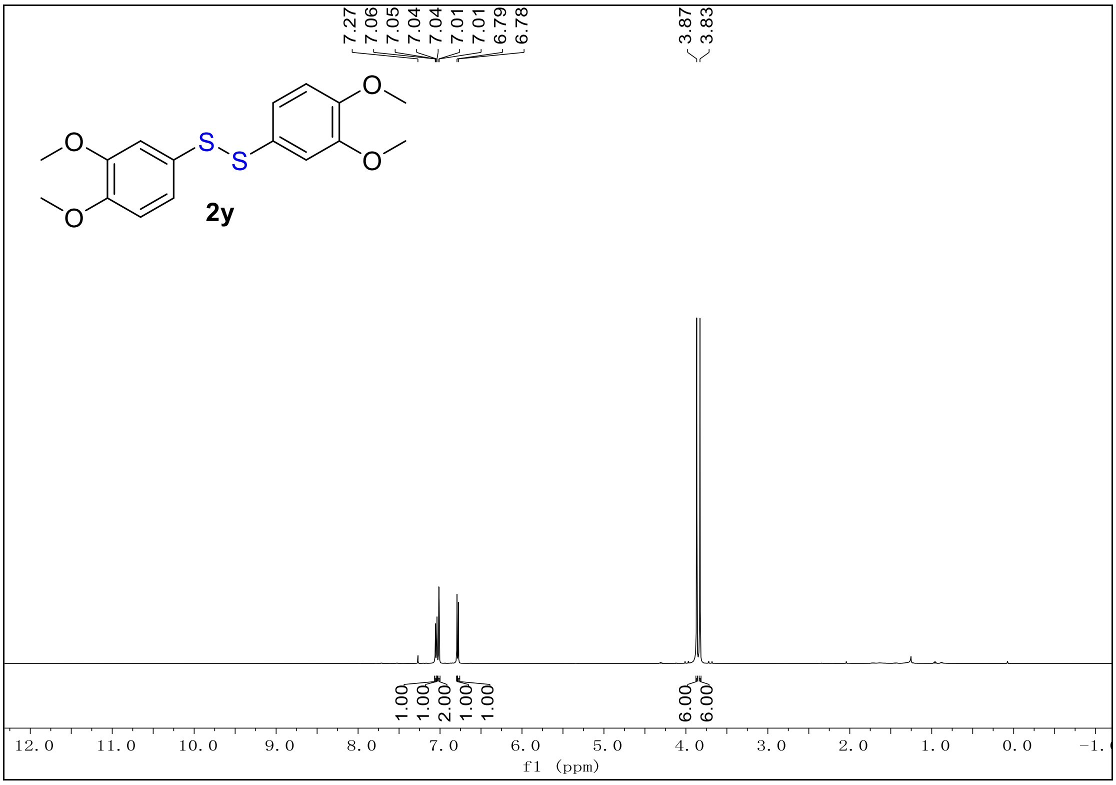
7.27
7.06
7.05
7.04
7.04
7.01
7.01
6.79
6.78
3.87
3.83
2y
1.00
1.00
2.00
1.00
1.00
6.00
6.00
12.0 11.0 10.0 9.0 8.0 7.0 6.0 5.0 4.0 3.0 2.0 1.0 0.0
f1 (ppm)

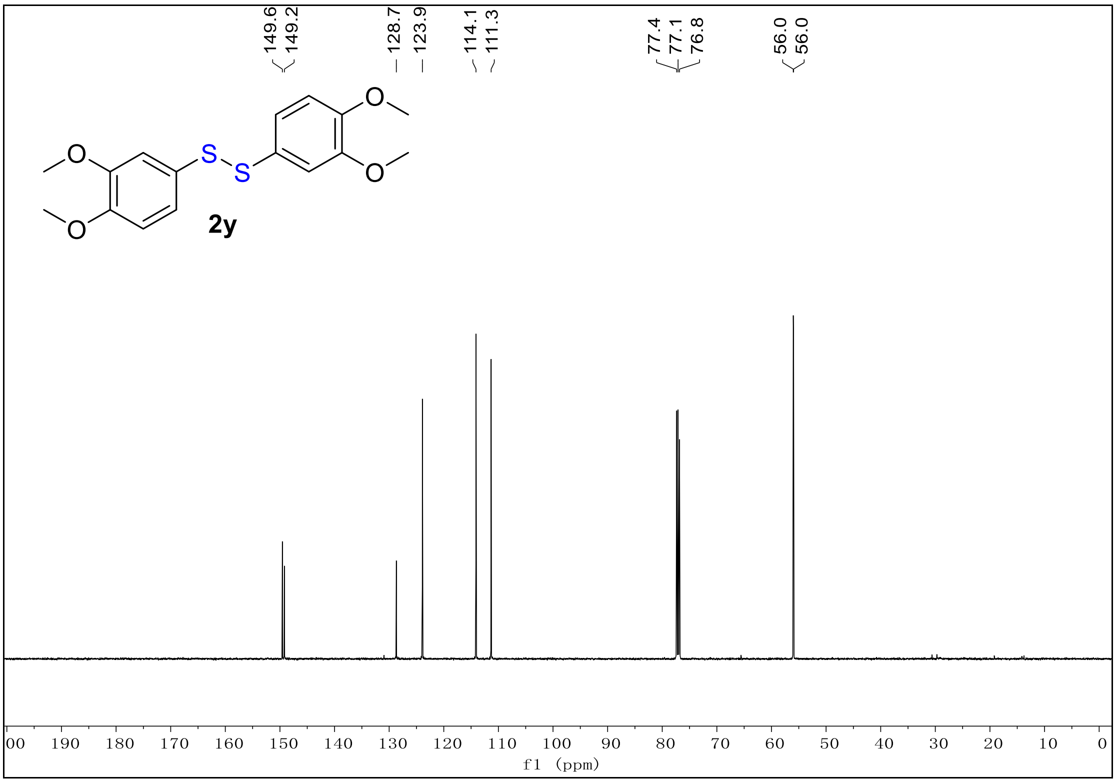
149.6
149.2
128.7
123.9
114.1
111.3
77.4
77.1
76.8
56.0
56.0
2y
190 180 170 160 150 140 130 120 110 100 90 80 70 60 50 40 30 20 10 0
f1 (ppm)

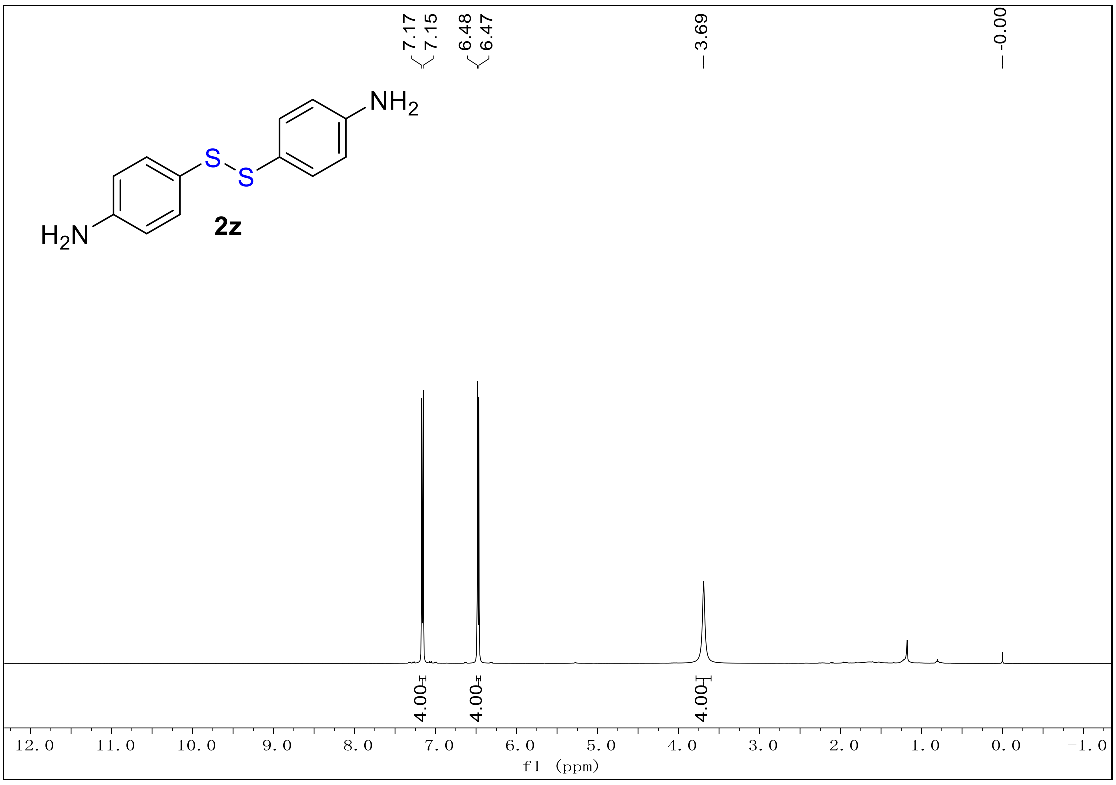
7.17
7.15
6.48
6.47
3.69
-0.00
NH2
S
S
H2N
2z
4.00
4.00
4.00
12.0 11.0 10.0 9.0 8.0 7.0 6.0 5.0 4.0 3.0 2.0 1.0 0.0 -1.0
f1 (ppm)

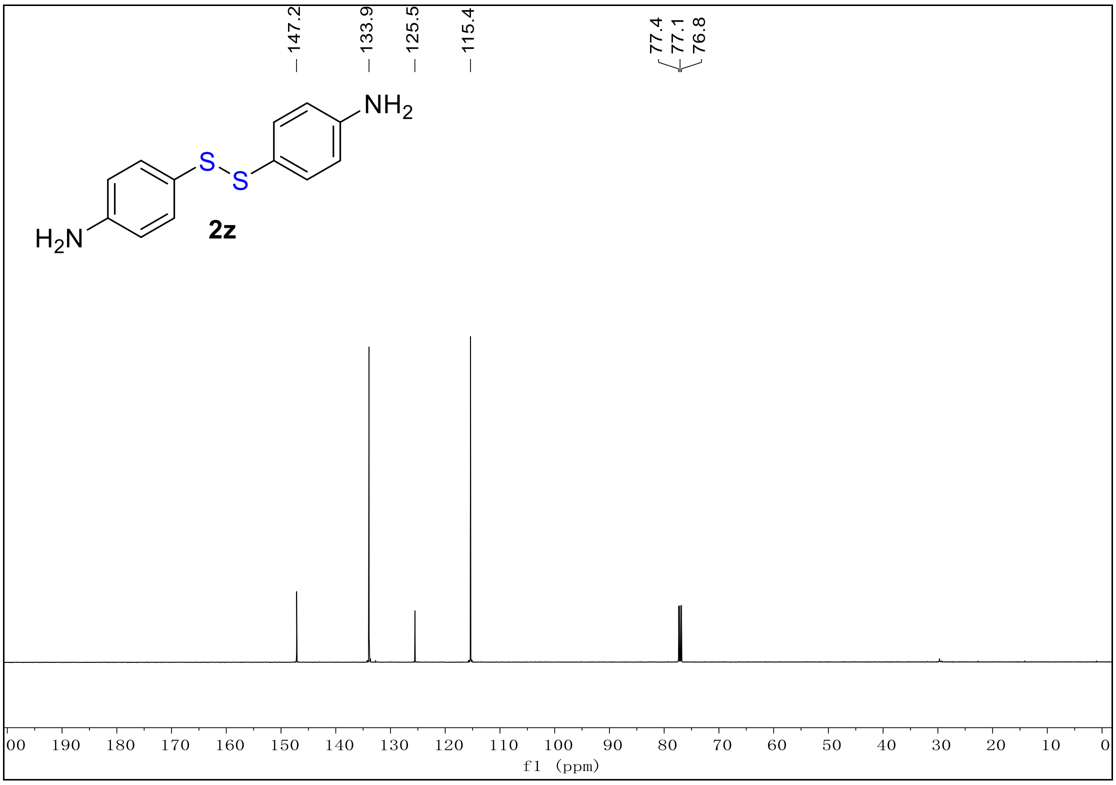
147.2
133.9
125.5
115.4
77.4
77.1
76.8
NH2
S
S
H2N
2z
00 190 180 170 160 150 140 130 120 110 100 90 80 70 60 50 40 30 20 10 0
f1 (ppm)

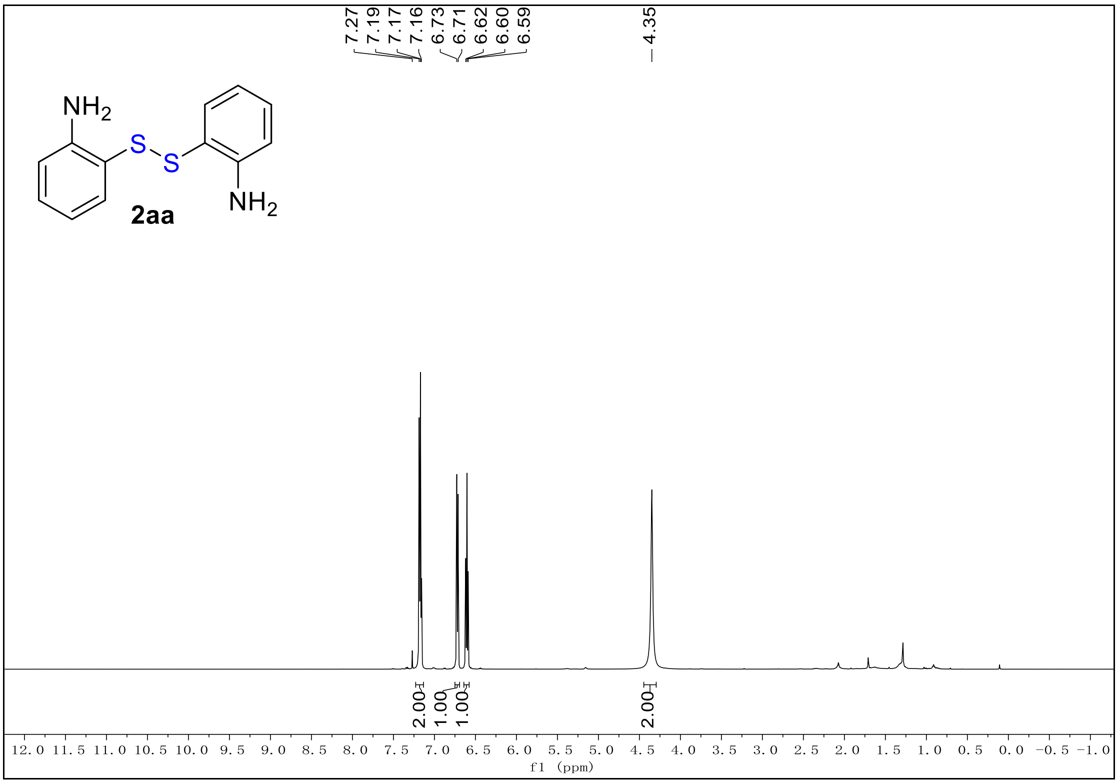
7.27
7.19
7.17
7.16
6.73
6.71
6.62
6.60
6.59
4.35
NH2
S
S
NH2
2aa
2.00
1.00
1.00
2.00
12.0 11.5 11.0 10.5 10.0 9.5 9.0 8.5 8.0 7.5 7.0 6.5 6.0 5.5 5.0 4.5 4.0 3.5 3.0 2.5 2.0 1.5 1.0 0.5 0.0 -0.5 -1.0
f1 (ppm)

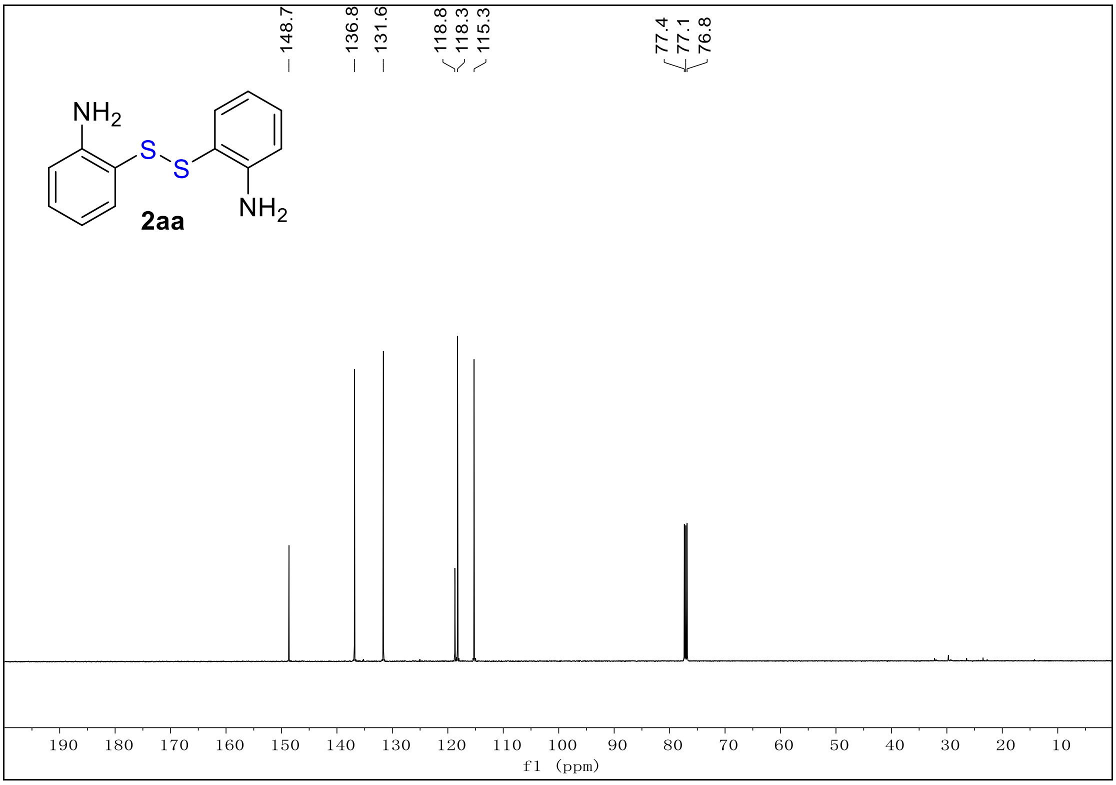
148.7
136.8
131.6
118.8
118.3
115.3
77.4
77.1
76.8
NH2
S
S
NH2
2aa
190 180 170 160 150 140 130 120 110 100 90 80 70 60 50 40 30 20 10
f1 (ppm)

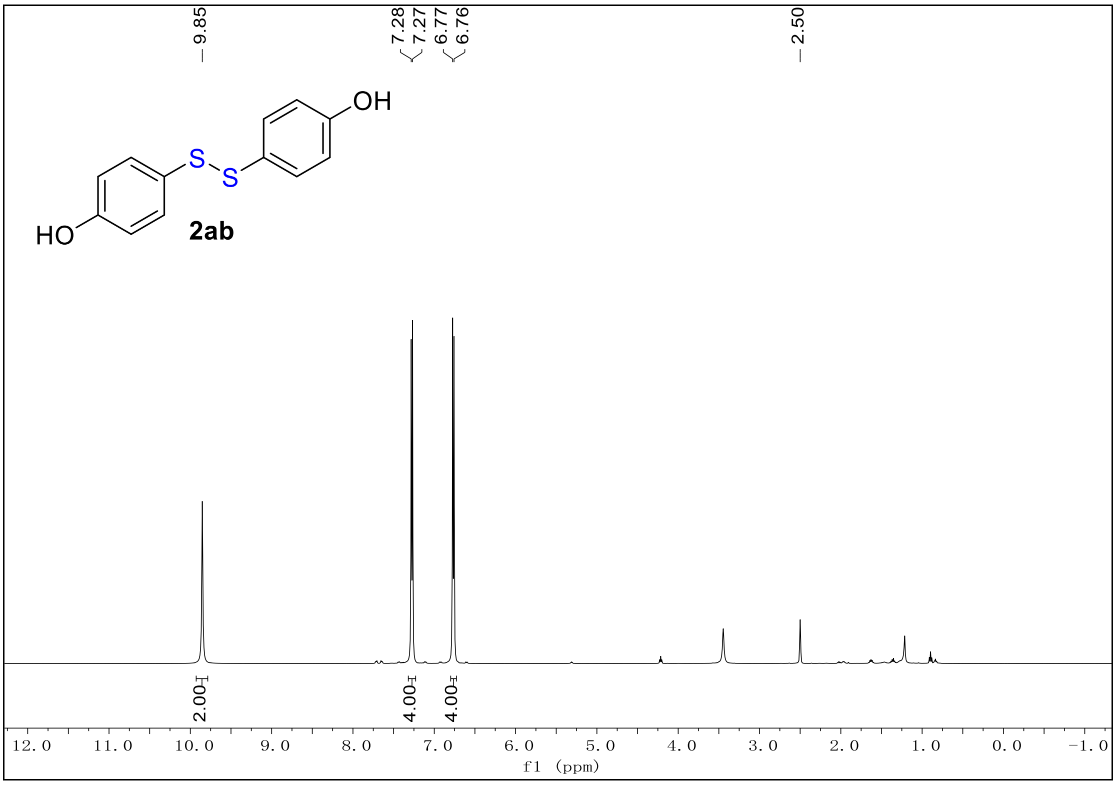
9.85
7.28
7.27
6.77
6.76
2.50
OH
S
S
HO
2ab
2.00
4.00
4.00
12.0 11.0 10.0 9.0 8.0 7.0 6.0 5.0 4.0 3.0 2.0 1.0 0.0 -1.0
f1 (ppm)

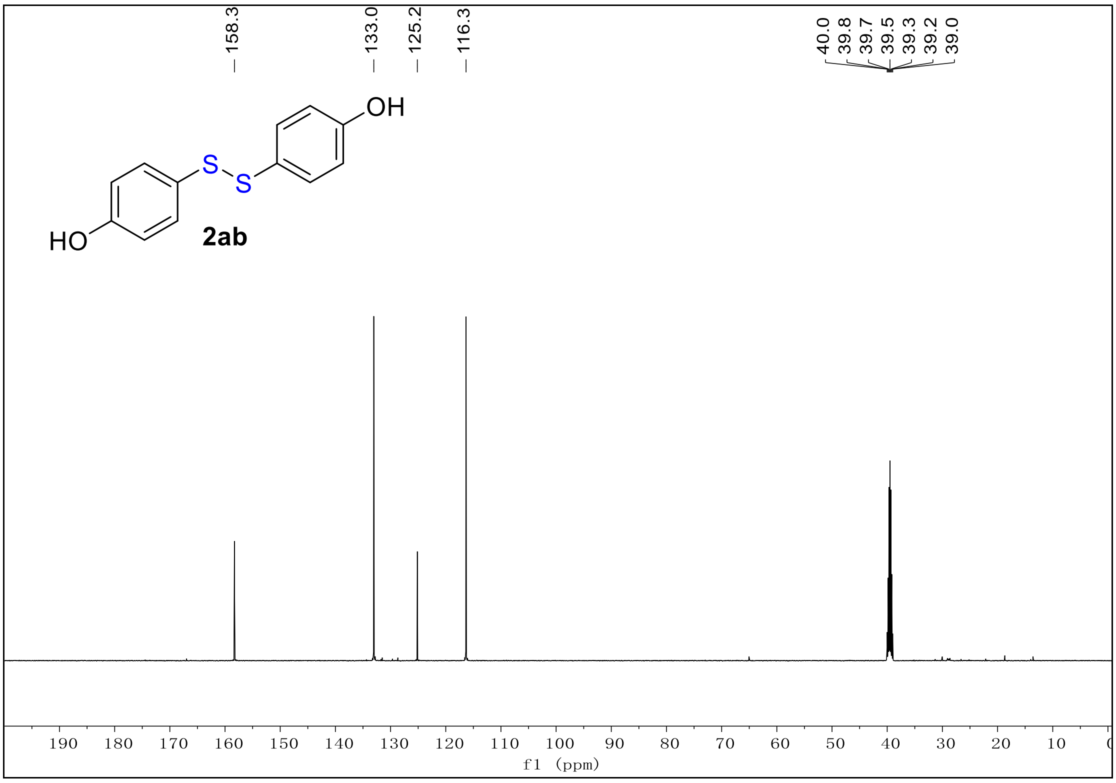
158.3
133.0
125.2
116.3
40.0
39.8
39.7
39.5
39.3
39.2
39.0
OH
S
S
HO
2ab
190 180 170 160 150 140 130 120 110 100 90 80 70 60 50 40 30 20 10
f1 (ppm)

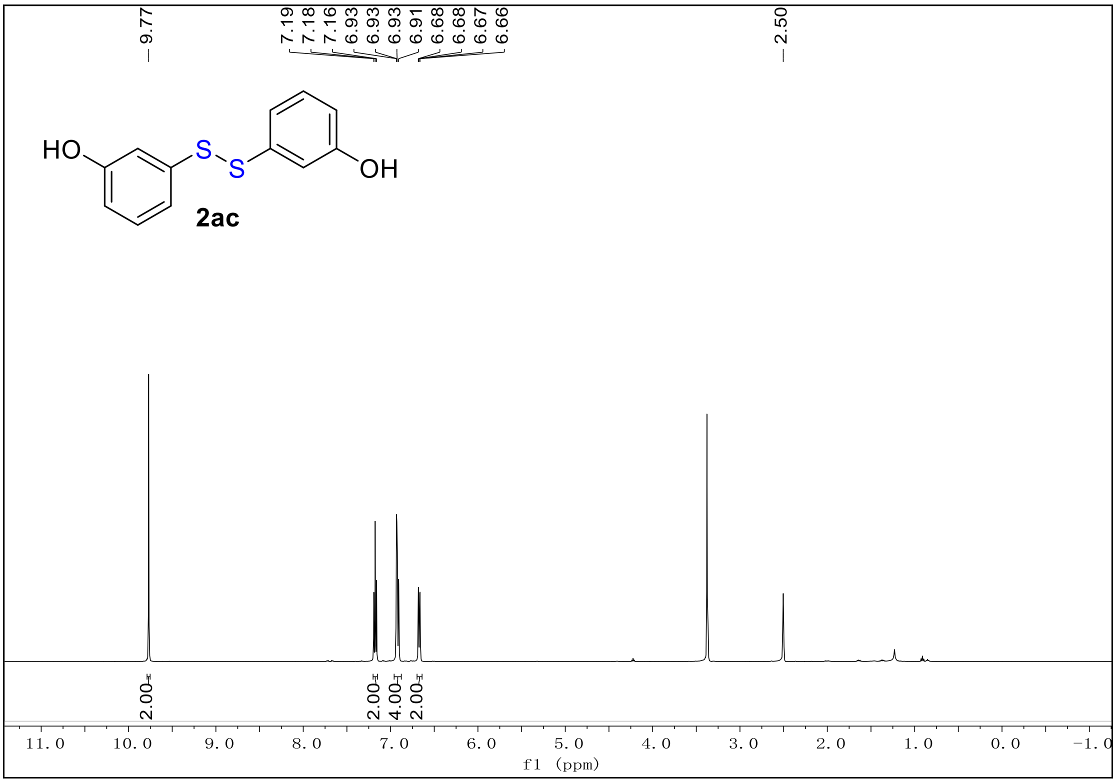

9.77
7.19
7.18
7.16
6.93
6.93
6.93
6.91
6.68
6.68
6.67
6.66
2.50
HO
S
S
OH
2ac
2.00
2.00
4.00
2.00
11.0 10.0 9.0 8.0 7.0 6.0 5.0 4.0 3.0 2.0 1.0 0.0 -1.0
f1 (ppm)

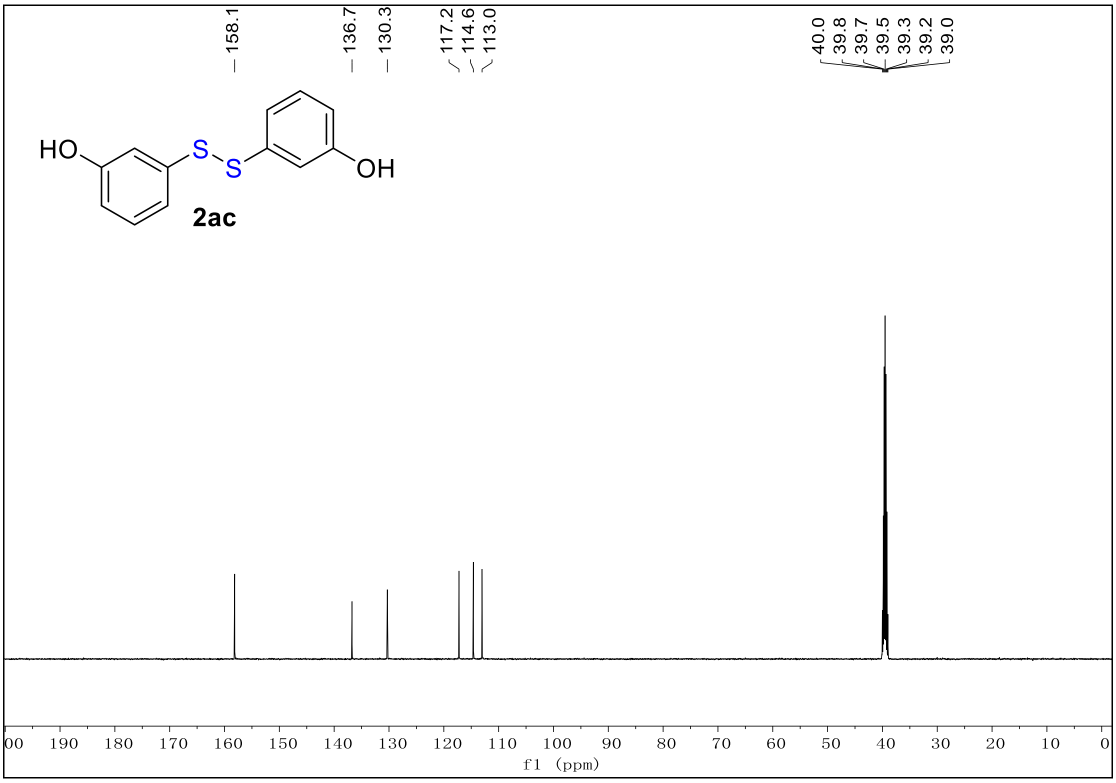

158.1
136.7
130.3
117.2
114.6
113.0
40.0
39.8
39.7
39.5
39.3
39.2
39.0
HO
S
S
OH
2ac
00 190 180 170 160 150 140 130 120 110 100 90 80 70 60 50 40 30 20 10 0
f1 (ppm)

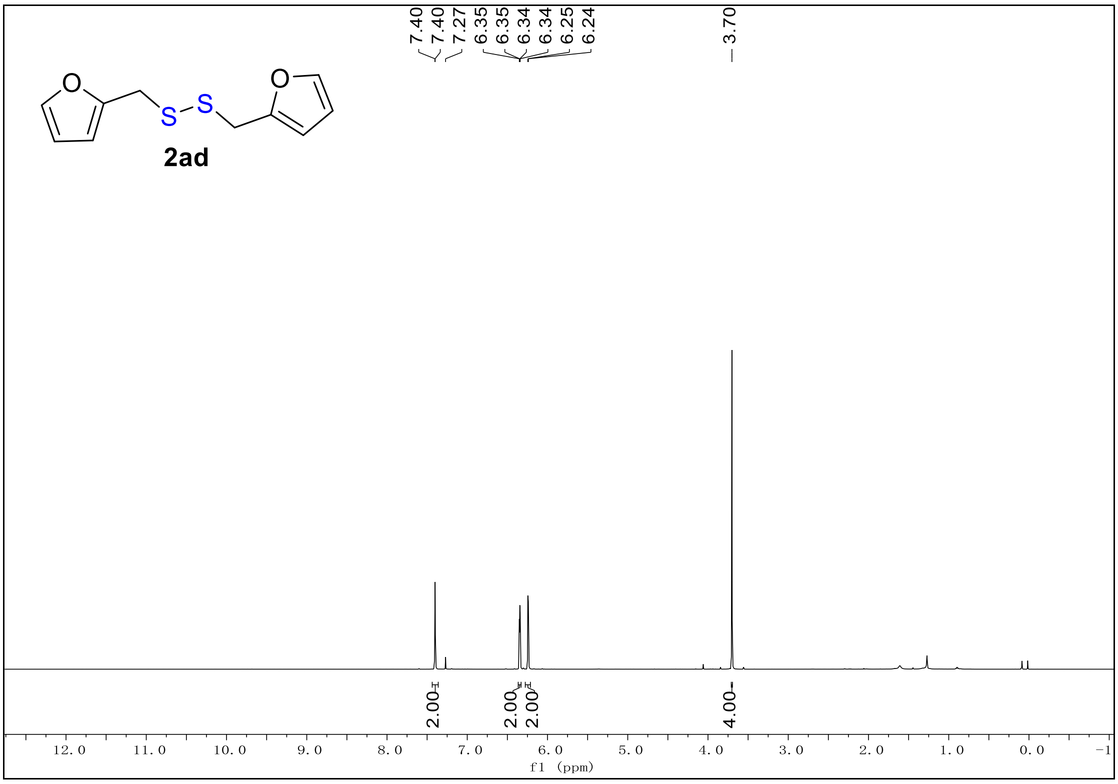
7.40
7.40
7.27
6.35
6.35
6.34
6.34
6.25
6.24
3.70
O
S
S
O
2ad
2.00
2.00
2.00
4.00
12.0 11.0 10.0 9.0 8.0 7.0 6.0 5.0 4.0 3.0 2.0 1.0 0.0
f1 (ppm)

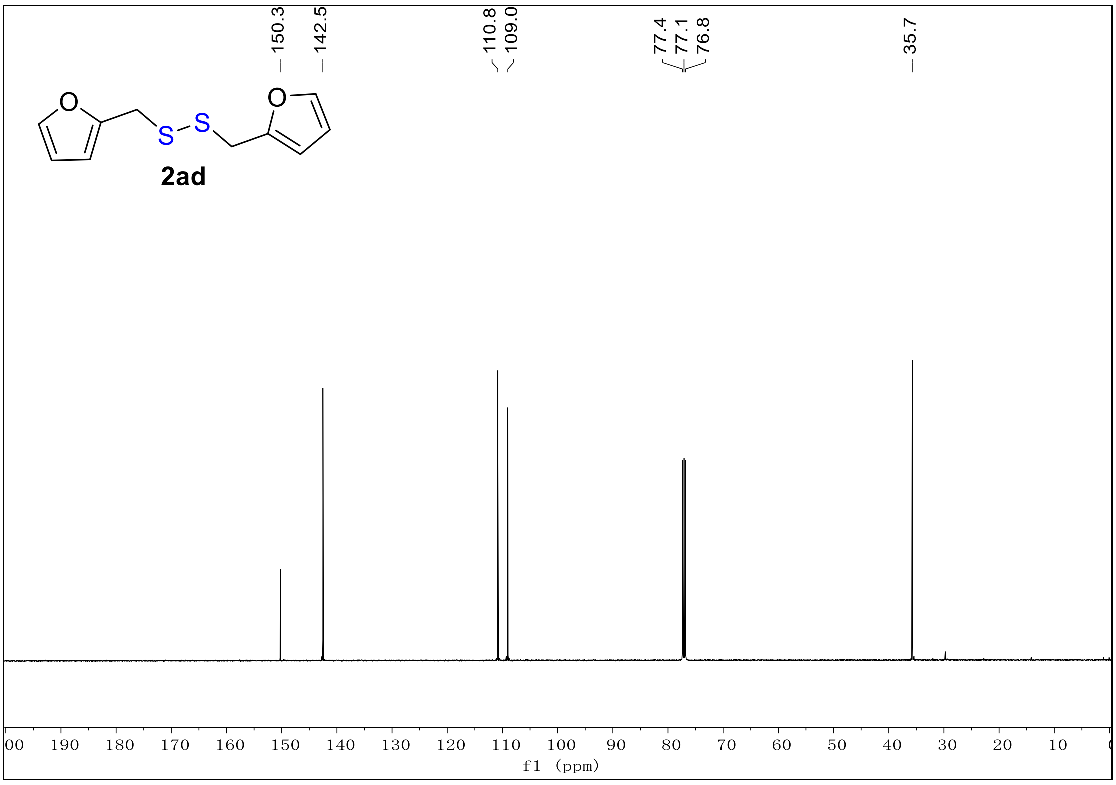
150.3
142.5
110.8
109.0
77.4
77.1
76.8
35.7
O
S
S
O
2ad
190 180 170 160 150 140 130 120 110 100 90 80 70 60 50 40 30 20 10
f1 (ppm)

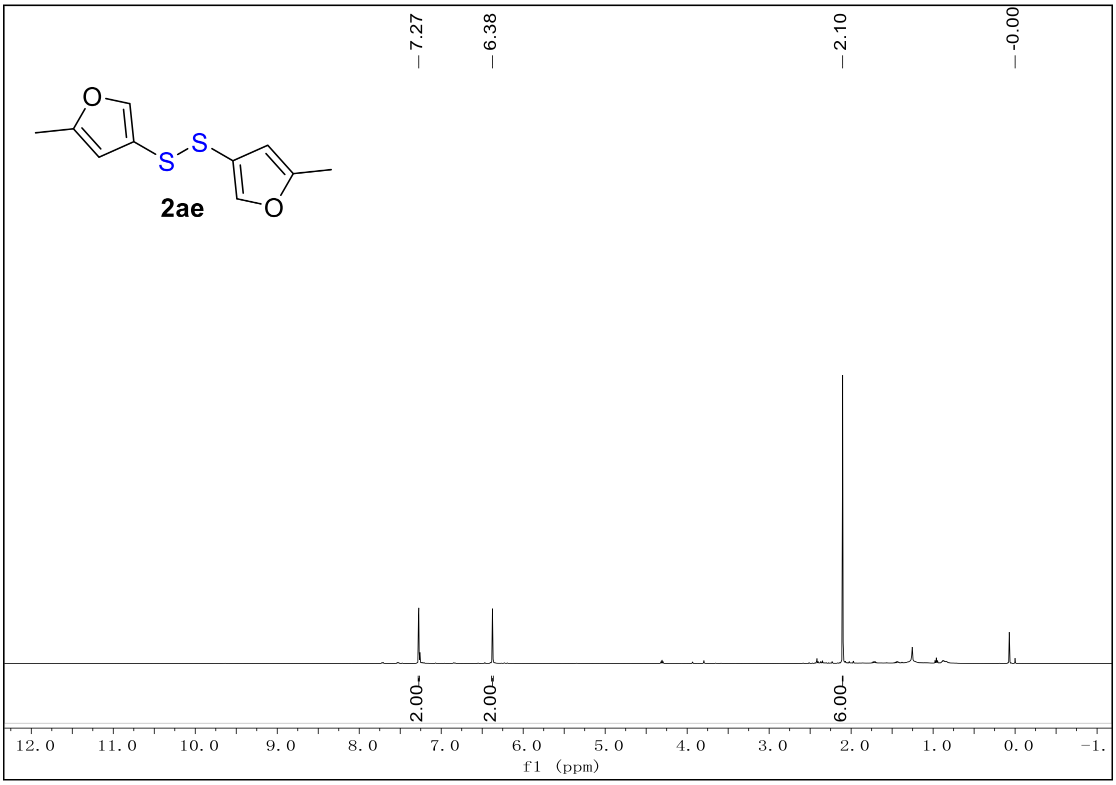
7.27
6.38
2.10
-0.00
2ae
2.00
2.00
6.00
12.0
11.0
10.0
9.0
8.0
7.0
6.0
5.0
4.0
3.0
2.0
1.0
0.0
-1.
f1 (ppm)

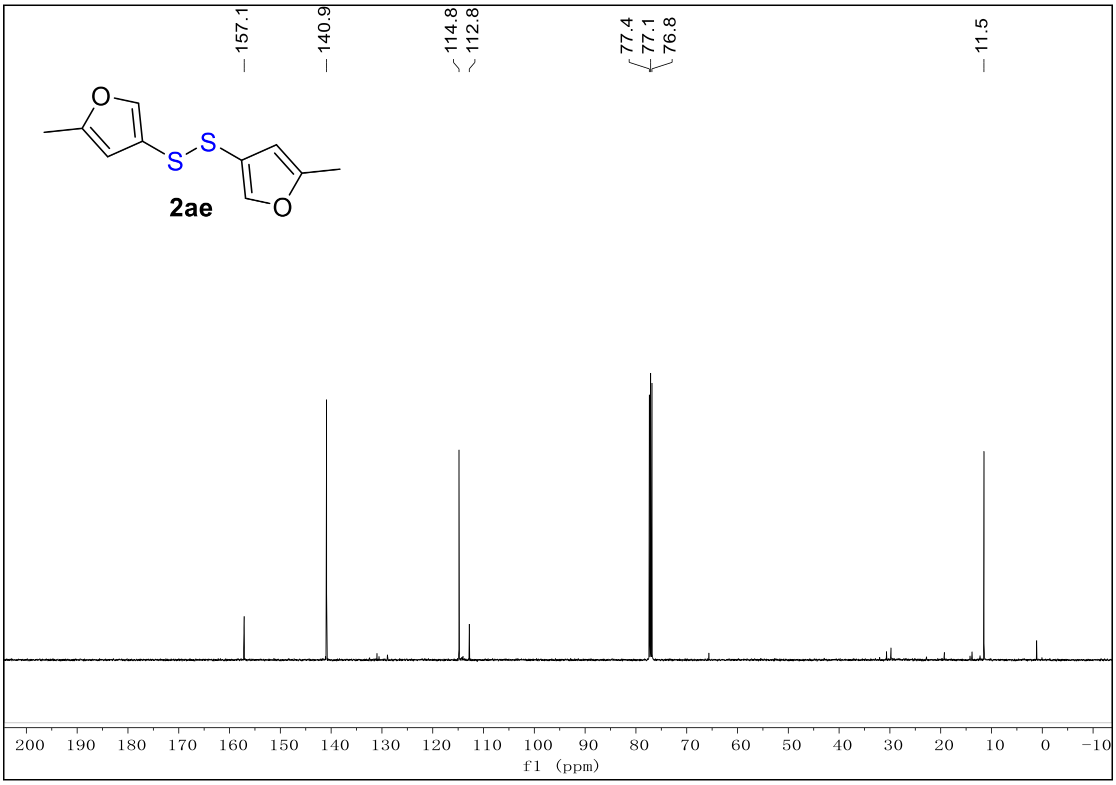
157.1
140.9
114.8
112.8
77.4
77.1
76.8
11.5
2ae
200
190
180
170
160
150
140
130
120
110
100
90
80
70
60
50
40
30
20
10
0
-10
f1 (ppm)

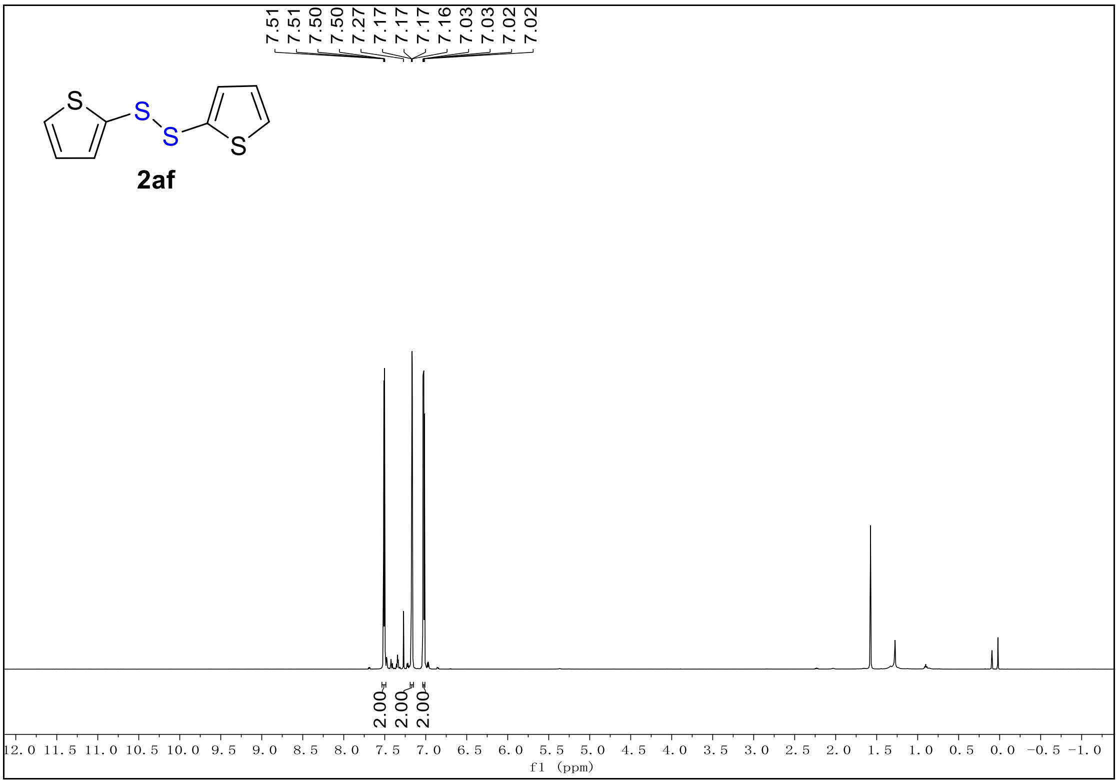
7.51
7.51
7.50
7.50
7.27
7.17
7.17
7.17
7.16
7.03
7.03
7.02
7.02
2af
2.00
2.00
2.00
12.0 11.5 11.0 10.5 10.0 9.5 9.0 8.5 8.0 7.5 7.0 6.5 6.0 5.5 5.0 4.5 4.0 3.5 3.0 2.5 2.0 1.5 1.0 0.5 0.0 -0.5 -1.0
f1 (ppm)

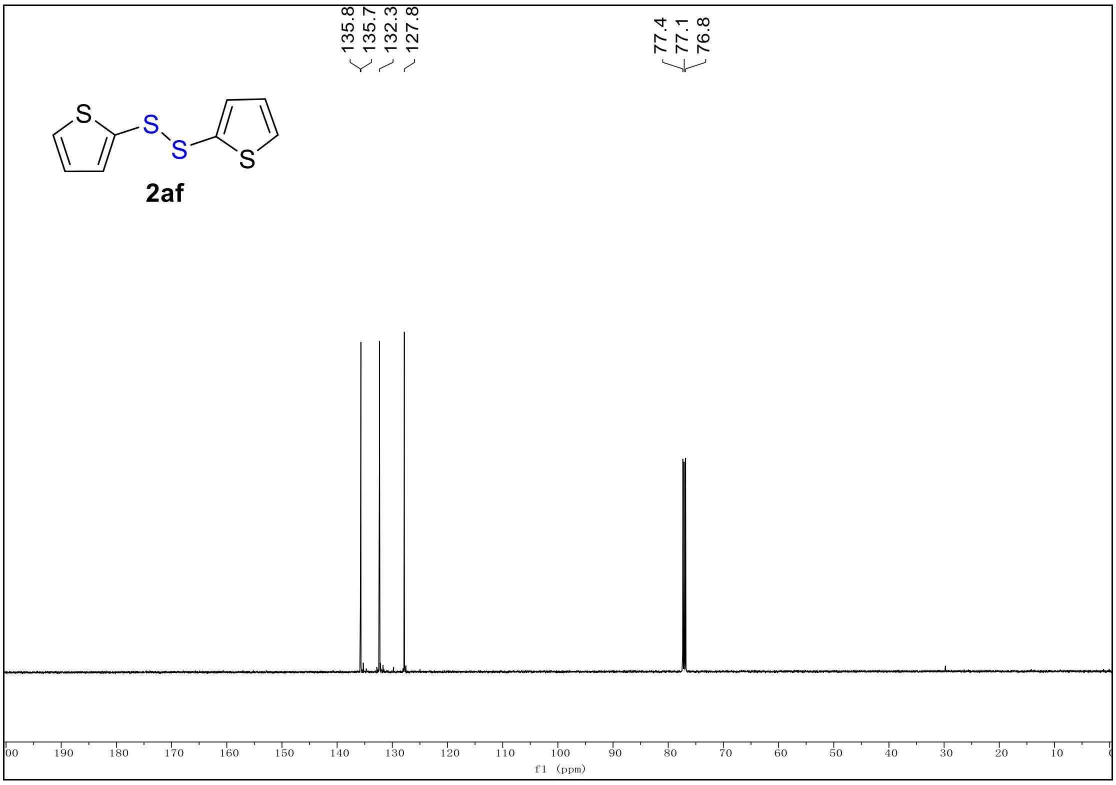
135.8
135.7
132.3
127.8
77.4
77.1
76.8
2af
190 180 170 160 150 140 130 120 110 100 90 80 70 60 50 40 30 20 10
f1 (ppm)

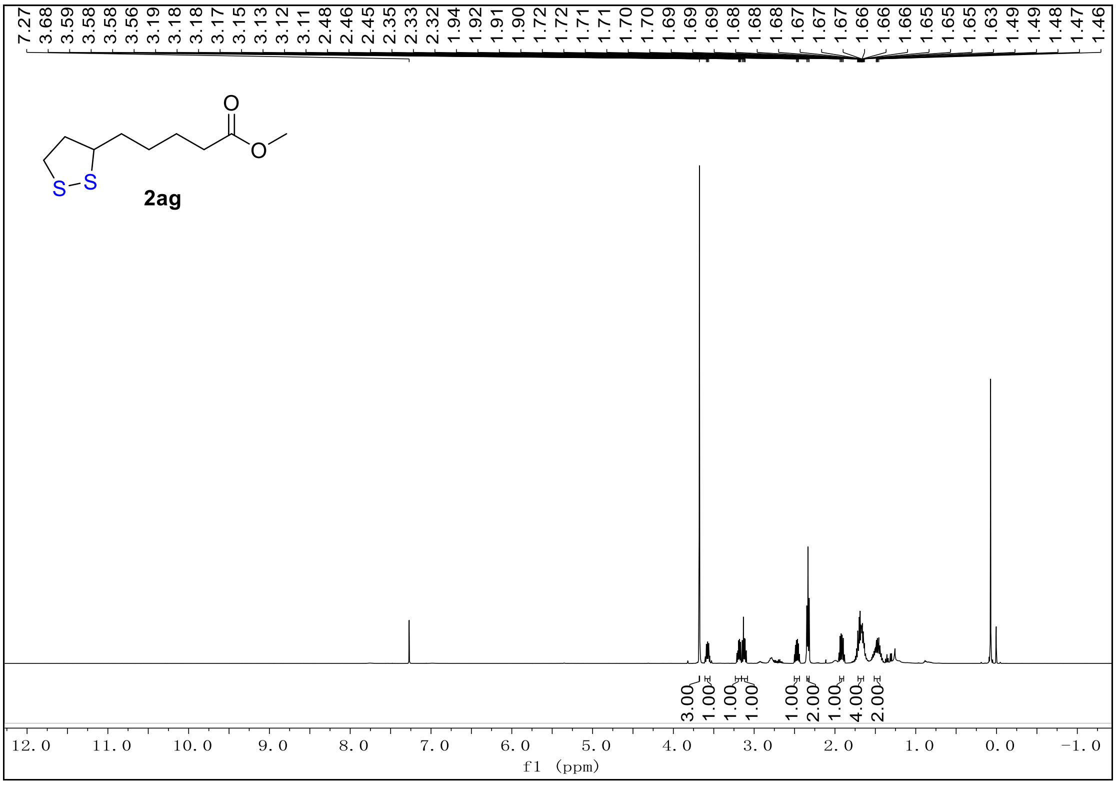
7.27 3.68 3.59 3.58 3.58 3.56 3.19 3.18 3.18 3.17 3.15 3.13 3.12 3.11 2.48 2.46 2.45 2.35 2.33 2.32 1.94 1.92 1.91 1.90 1.72 1.72 1.71 1.71 1.70 1.70 1.69 1.69 1.68 1.68 1.68 1.67 1.67 1.67 1.66 1.66 1.66 1.65 1.65 1.63 1.49 1.48 1.47 1.46
O
O
S
S
2ag
3.00 1.00 1.00 1.00 1.00 2.00 1.00 4.00 2.00
12.0 11.0 10.0 9.0 8.0 7.0 6.0 5.0 4.0 3.0 2.0 1.0 0.0 -1.0
f1 (ppm)

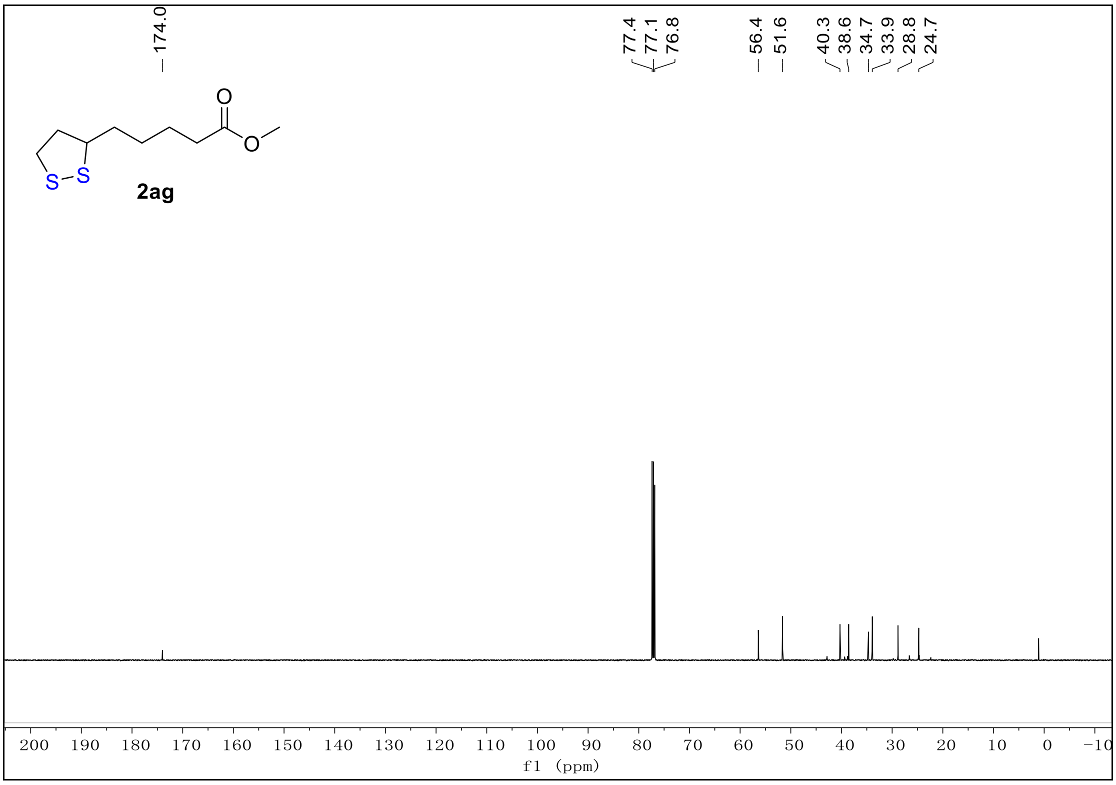
174.0
77.4 77.1 76.8
56.4 51.6
40.3 38.6 34.7 33.9 28.8 24.7
O
O
S
S
2ag
200 190 180 170 160 150 140 130 120 110 100 90 80 70 60 50 40 30 20 10 0 -10
f1 (ppm)

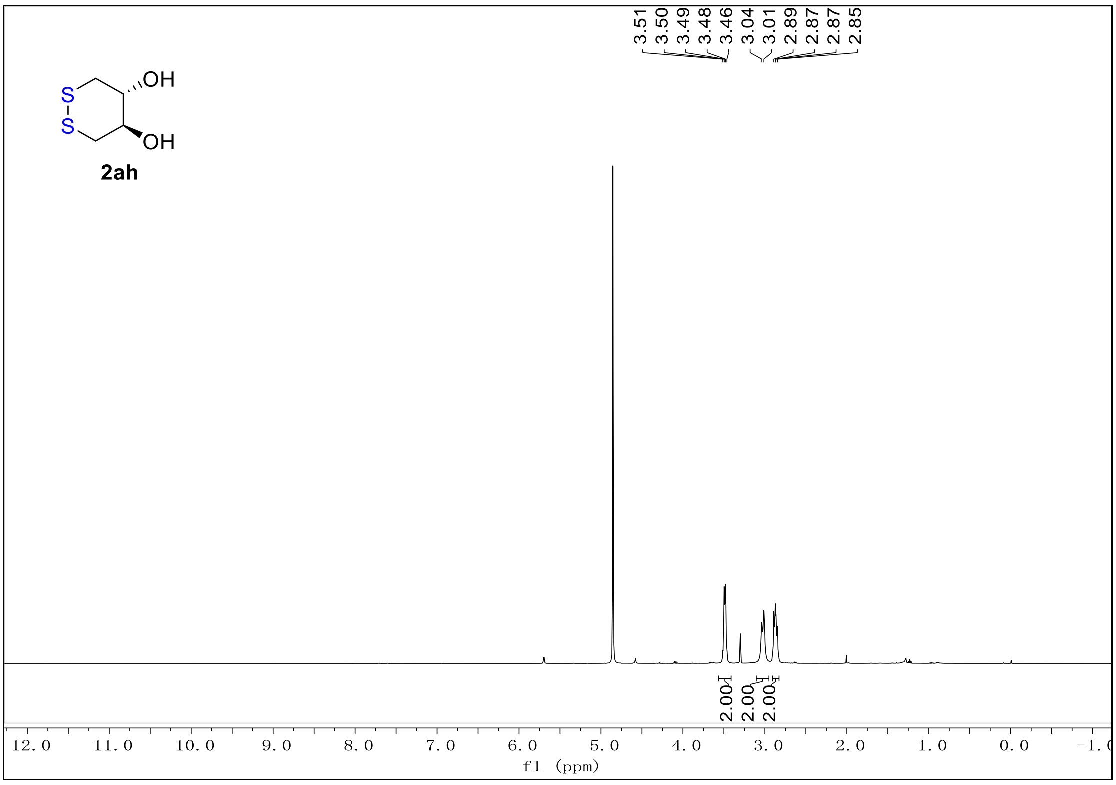

OH
S
S
OH
2ah
3.51
3.50
3.49
3.48
3.46
3.04
3.01
2.89
2.87
2.87
2.85
2.00
2.00
2.00
12.0 11.0 10.0 9.0 8.0 7.0 6.0 5.0 4.0 3.0 2.0 1.0 0.0 -1.0
f1 (ppm)

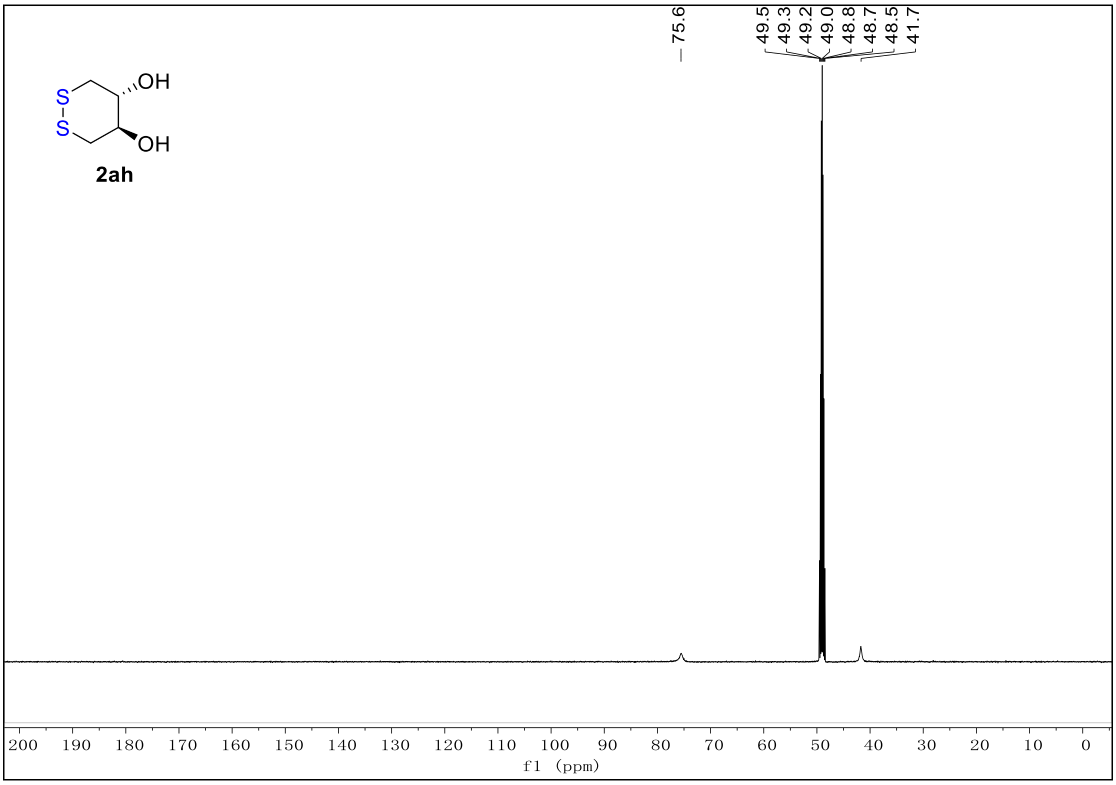

OH
S
S
OH
2ah
75.6
49.5
49.3
49.2
49.0
48.8
48.7
48.5
41.7
200 190 180 170 160 150 140 130 120 110 100 90 80 70 60 50 40 30 20 10 0
f1 (ppm)

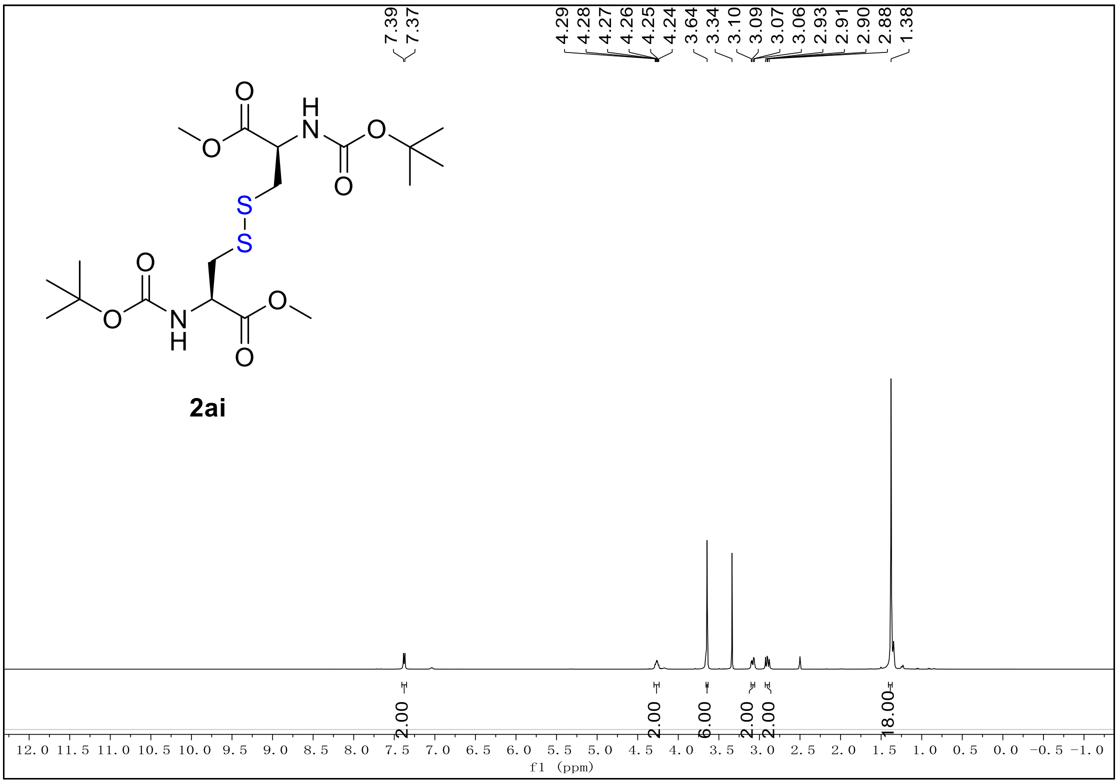

7.39
7.37
4.29
4.28
4.27
4.26
4.25
4.24
3.64
3.34
3.10
3.09
3.07
3.06
2.93
2.91
2.90
2.88
1.38
2ai
2.00
2.00
6.00
2.00
2.00
18.00
12.0 11.5 11.0 10.5 10.0 9.5 9.0 8.5 8.0 7.5 7.0 6.5 6.0 5.5 5.0 4.5 4.0 3.5 3.0 2.5 2.0 1.5 1.0 0.5 0.0 -0.5 -1.0
f1 (ppm)

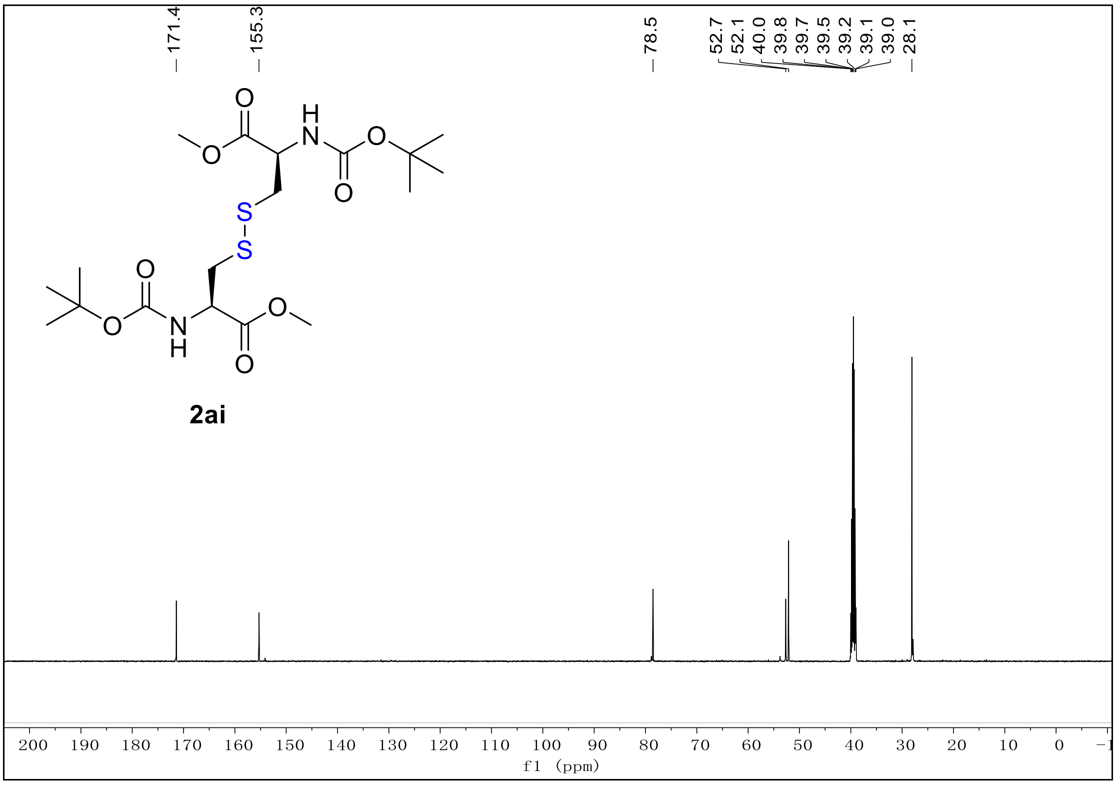

171.4
155.3
78.5
52.7
52.1
40.0
39.8
39.7
39.5
39.2
39.1
39.0
28.1
2ai
200 190 180 170 160 150 140 130 120 110 100 90 80 70 60 50 40 30 20 10 0
f1 (ppm)

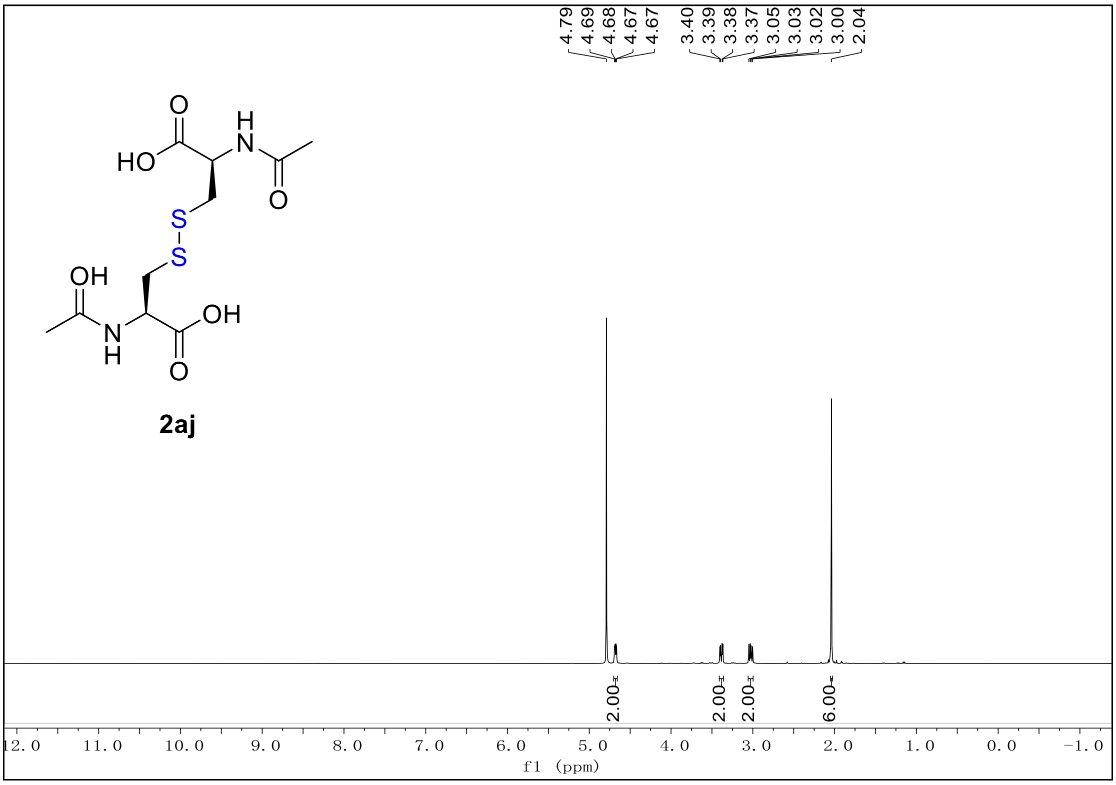
4.79
4.69
4.68
4.67
4.67
3.40
3.39
3.38
3.37
3.05
3.03
3.02
3.00
2.04
HO
O
H
N
O
S
S
OH
N
H
OH
O
2aj
2.00
2.00
2.00
6.00
12.0
11.0
10.0
9.0
8.0
7.0
6.0
5.0
4.0
3.0
2.0
1.0
0.0
-1.0
f1 (ppm)

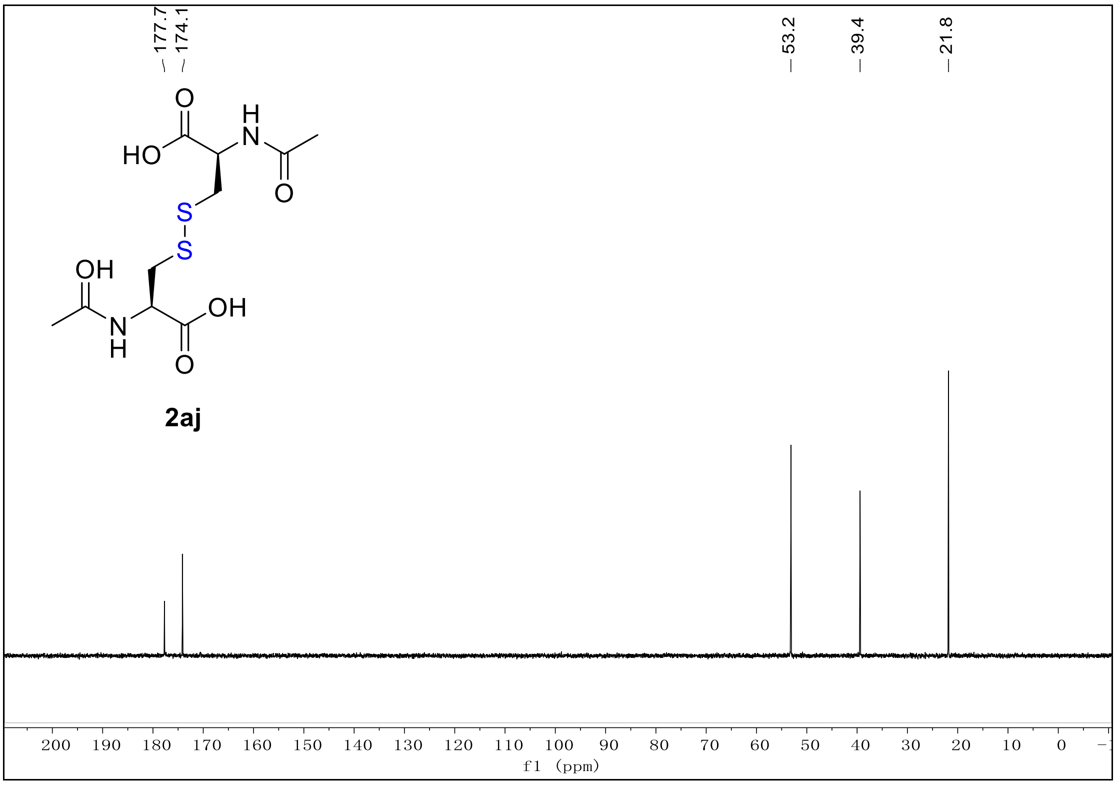
177.7
174.1
53.2
39.4
21.8
HO
O
H
N
O
S
S
OH
N
H
OH
O
2aj
200
190
180
170
160
150
140
130
120
110
100
90
80
70
60
50
40
30
20
10
0
f1 (ppm)

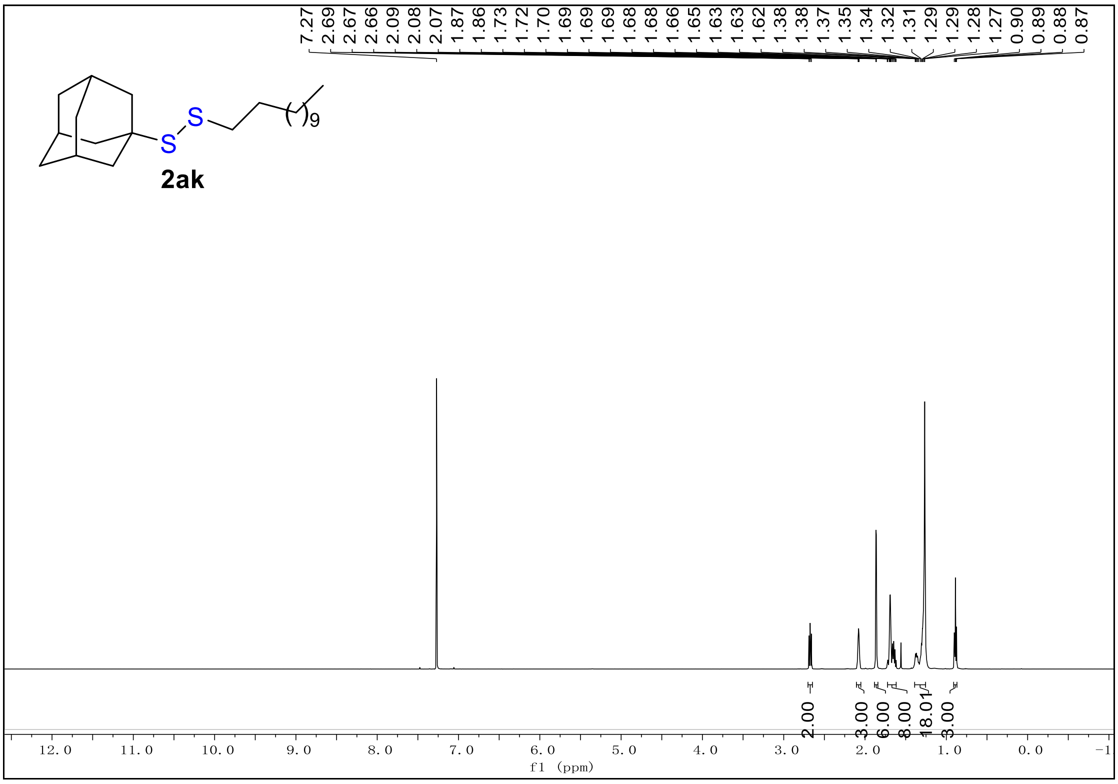
7.27 2.69 2.67 2.66 2.09 2.08 2.07 1.87 1.86 1.73 1.72 1.70 1.69 1.69 1.69 1.68 1.68 1.66 1.65 1.63 1.63 1.62 1.38 1.38 1.37 1.35 1.34 1.32 1.31 1.29 1.29 1.28 1.27 0.90 0.89 0.88 0.87
S
S
9
2ak
2.00
3.00
6.00
8.00
18.01
3.00
12.0 11.0 10.0 9.0 8.0 7.0 6.0 5.0 4.0 3.0 2.0 1.0 0.0 -1
f1 (ppm)

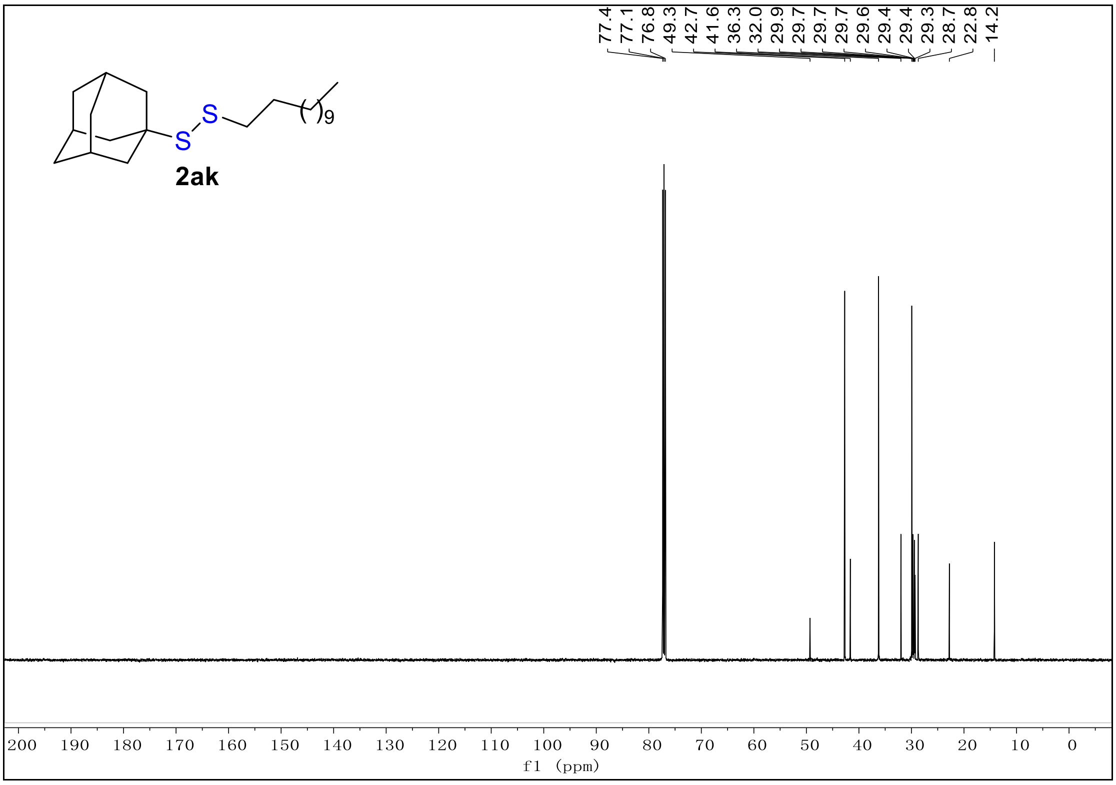
77.4 77.1 76.8 49.3 42.7 41.6 36.3 32.0 29.9 29.7 29.7 29.7 29.6 29.4 29.4 29.3 28.7 22.8 14.2
S
S
9
2ak
200 190 180 170 160 150 140 130 120 110 100 90 80 70 60 50 40 30 20 10 0
f1 (ppm)

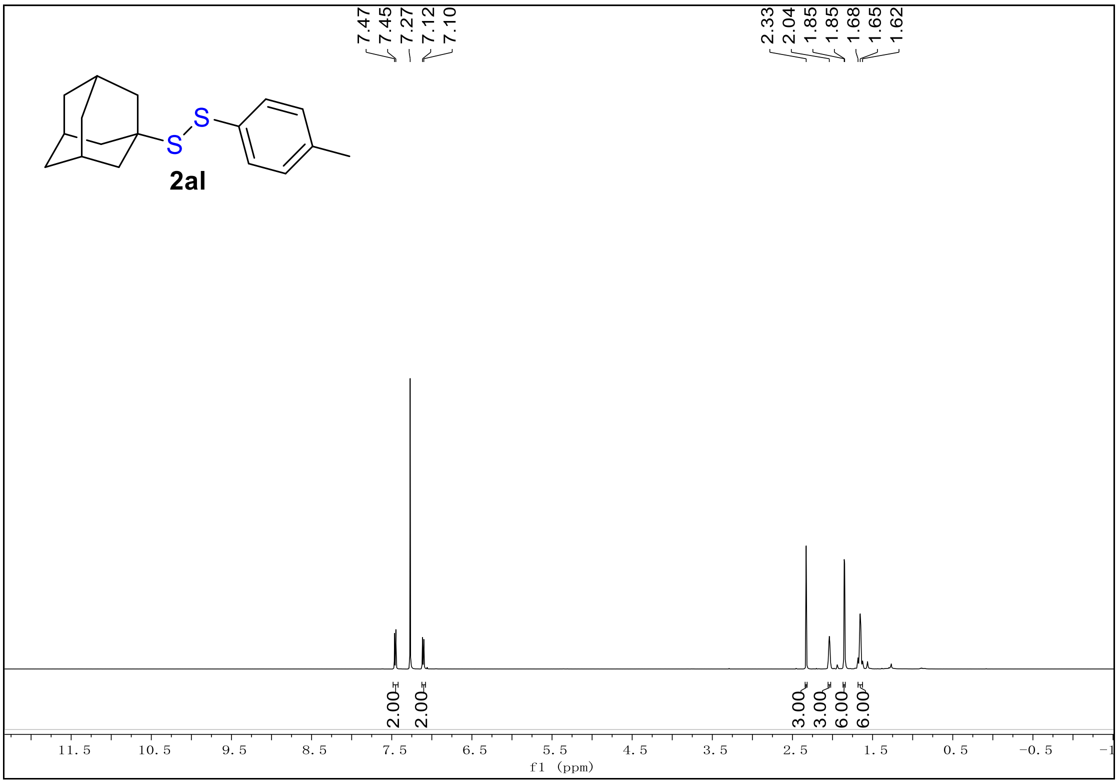

7.47
7.45
7.27
7.12
7.10
2.33
2.04
1.85
1.85
1.68
1.65
1.62
S
S
2al
2.00
2.00
3.00
3.00
6.00
6.00
11.5 10.5 9.5 8.5 7.5 6.5 5.5 4.5 3.5 2.5 1.5 0.5 -0.5 -1
f1 (ppm)

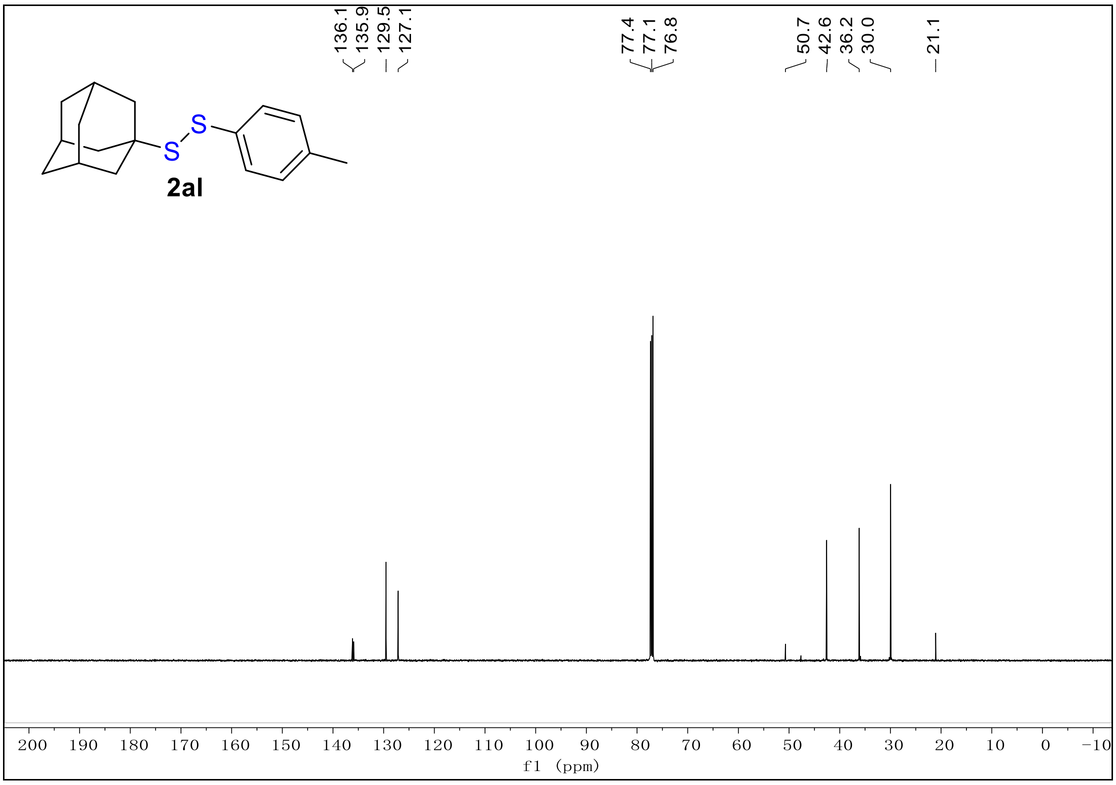

136.1
135.9
129.5
127.1
77.4
77.1
76.8
50.7
42.6
36.2
30.0
21.1
S
S
2al
200 190 180 170 160 150 140 130 120 110 100 90 80 70 60 50 40 30 20 10 0 -10
f1 (ppm)

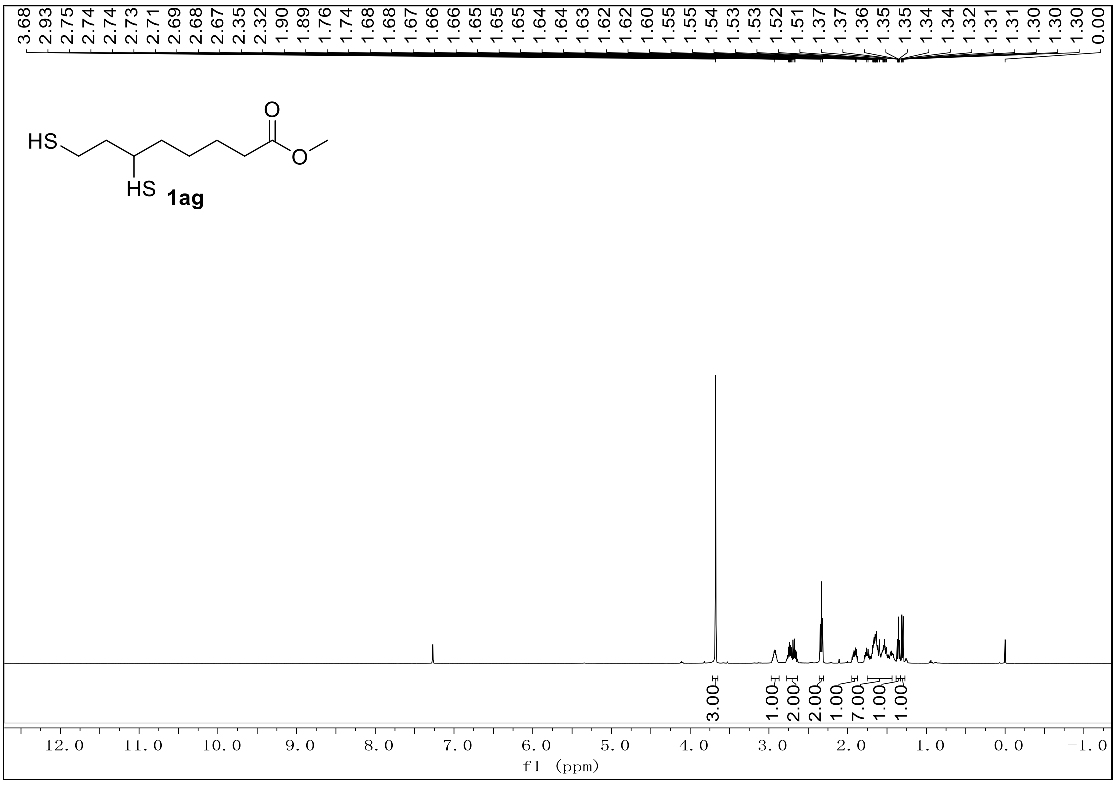
3.68 2.93 2.75 2.74 2.74 2.73 2.71 2.69 2.68 2.67 2.35 2.32 1.90 1.89 1.76 1.74 1.68 1.68 1.67 1.66 1.66 1.65 1.65 1.64 1.64 1.63 1.62 1.62 1.60 1.55 1.55 1.54 1.53 1.53 1.52 1.51 1.37 1.37 1.36 1.35 1.34 1.34 1.32 1.31 1.30 1.30 1.30 0.00
HS
O
O
HS
1ag
3.00
1.00
2.00
2.00
1.00
7.00
1.00
1.00
12.0 11.0 10.0 9.0 8.0 7.0 6.0 5.0 4.0 3.0 2.0 1.0 0.0 -1.0
f1 (ppm)

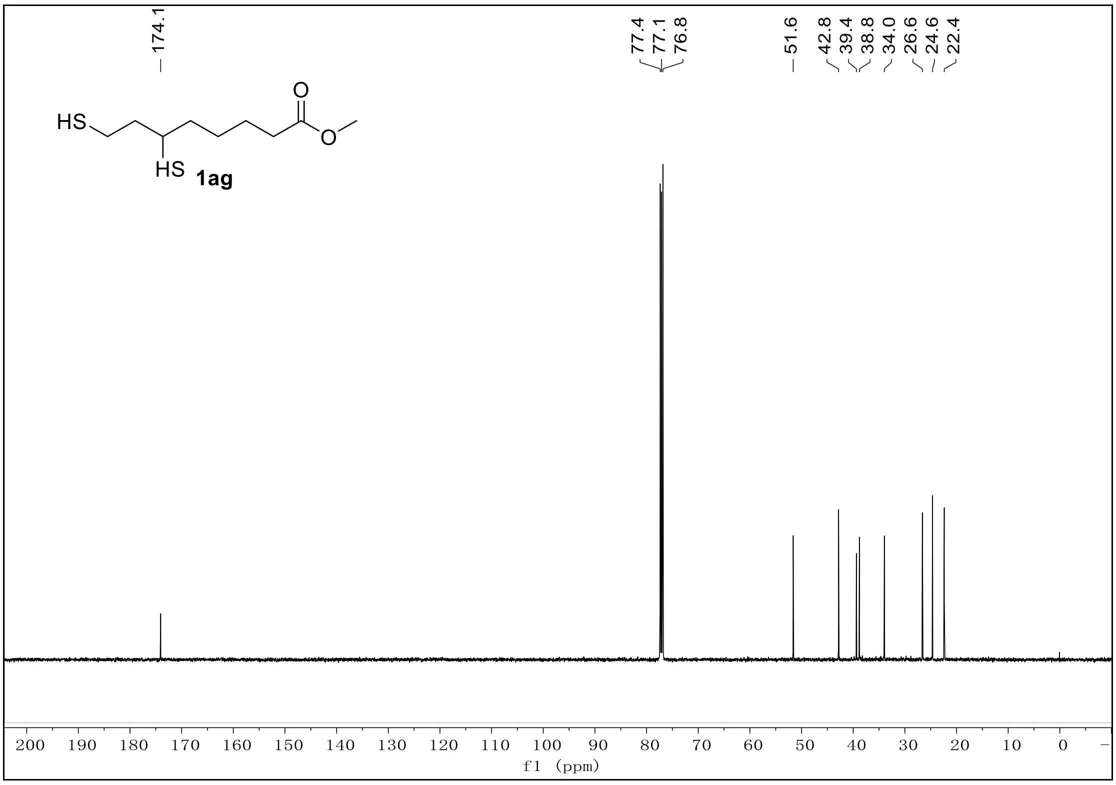
174.1
77.4 77.1 76.8
51.6
42.8 39.4 38.8 34.0 26.6 24.6 22.4
HS
O
O
HS
1ag
200 190 180 170 160 150 140 130 120 110 100 90 80 70 60 50 40 30 20 10 0
f1 (ppm)
